# The Influence of Chirality on the β-Amino-Acid Naphthalenediimides/G-Quadruplex DNA Interaction

**DOI:** 10.3390/molecules28217291

**Published:** 2023-10-27

**Authors:** Samuel R. Clowes, Yusuf Ali, Olivia R. Astley, Dora M. Răsădean, G. Dan Pantoş

**Affiliations:** Department of Chemistry, University of Bath, Claverton Down, Bath BA2 7AY, UK

**Keywords:** circular dichroism, enantioselectivity, G-quadruplex DNA, chiral recognition, docking

## Abstract

G-quadruplexes (G4s) have been identified as a potential alternative chemotherapy target. A series of eight β-amino acid derived naphthalenediimides (NDI) were screened against a series of oncogenic G4 sequences: c-KIT1, h-TELO, and TBA. Three sets of enantiomers were investigated to further our understanding of the effect of point chirality on G4 stabilisation. Enantioselective binding behaviour was observed with both c-KIT1 and h-TELO. Docking studies using GNINA and UV-vis titrations were employed to better understand this selective binding behaviour.

## 1. Introduction

**Figure 1:**
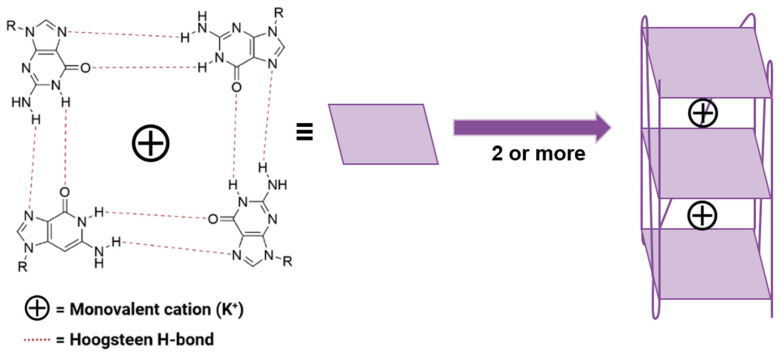
A cartoon showing the formation of G4s from four hydrogen bonded guanines, self-assembling via π–π stacking.

As a property ubiquitous in nature, the importance of chirality in drug design cannot be understated. Throughout the medical world, chirality plays an important role in drug design [1]. It is widely known that enantiomers can display vastly different biological properties with regard to metabolism, pharmacology, and toxicology [1,2,3,4,5]. An area where the influence point chirality is being explored more thoroughly is chemotherapeutics [6,7,8,9,10]. The identification of complementary targets as alternatives to classical chemotherapy has identified G-quadruplex DNA (G4 DNA) as a promising option as it is frequently overexpressed in promoter regions for numerous oncogenes and telomeres [11,12,13]. The structural motif is involved in several essential biological processes, including transcription and cell replication [10,14], making G4 DNA a vital target for anticancer therapy. G4 DNA sequences are defined by their guanine-rich (G-rich) composition that is capable of self-assembly into a four-stranded G-quadruplex (G4) via a quasi-square planar arrangement, bound together via Hoogsteen-type hydrogen bonding networks (Figure 1) [10,15]. The directionality of the backbone strands relative to the G-tetrads in the 5′-3′ direction defines the topology of the G4 as parallel, antiparallel, or hybrid.

As a scaffold for designing specific interaction with G4s, a compound class that has garnered significant interest for its G4 DNA-binding properties is the naphthalenediimide (NDI). An NDI is a mechanism of action with a G4 is two-fold; π–π stacking between the planar heteroaromatic surfaces, and an interaction with the DNA loops dictated by the NDI-sidechains. There are reports of di-, tri- and tetra-substituted NDI ligands with specific binding of G4 DNA [7,16,17,18,19,20].

Throughout the literature, the influence of chirality on the interaction of ligands with G4s is insufficiently explored [21,22,23,24]. Telomestatin^®^ and Quarfloxin^®^, the standards within G4 DNA-ligand chemistry, are chiral, yet little-to-no consideration of the implications of enantiomeric forms is given in the literature. Our group has previously reported the synthesis and use of NDI ligands for G4 binding with α-amino acids as the peripheral substituents [6,7]. The work showed that enantiomers of one of the ligands, *N*_ε_-Boc-Lys NDI, displayed opposing effects when interacting with G4 DNA.

In this study, we employ NDIs functionalised with β-amino acids in enantiomeric pairs and assess how the topology of the DNA affects the binding ability of the NDIs. Within this work, we highlight the effect of point chirality on the interaction with G4s within the human telomere (h-TELO), an aptamer for thrombin (TBA), and a promoter region for oncogenes: c-KIT1. TBA is an aptamer for the serine protease, thrombin, which is known to take part in the pathogenesis of several diseases including lung cancer and fibrosis [25,26]. c-KIT1 is commonly overexpressed in gastrointestinal stromal tumours and leukaemias [27,28,29]. In >85% of tumours, the telomerase enzyme, capable of adding TTAGGG repeats to the 3′ end of h-TELO DNA, is reactivated and leading to telomere regeneration and cell immortality [30]. To gauge the selectivity of our NDIs for G4s, we used a short double-stranded DNA (dsDNA) sequence as a control. Interaction with dsDNA should be minimal so as to ensure no adverse effects on healthy cells. The objective of this study is to develop G4-ligands that are selective for the quadruplex structure over the traditional Watson–Crick structure. We aim to add to the growing body of work, highlighting G4s as a viable alternative chemotherapy target.

## 2. Results and Discussion

### 2.1. Naphthalenediimide Design

**Figure 2:**
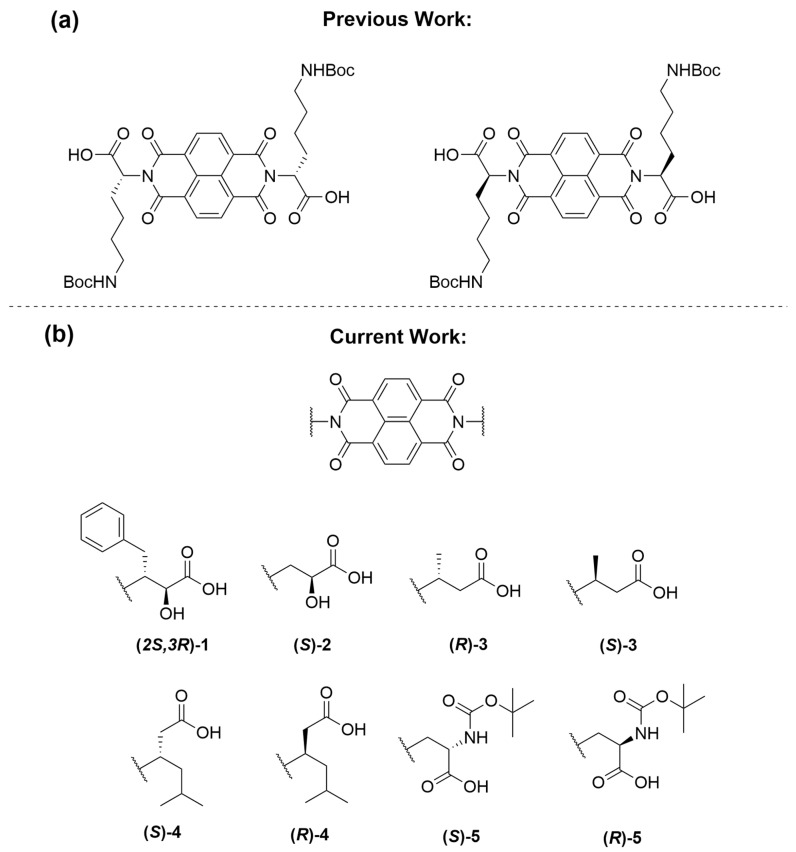
(**a**) The enantiomeric pair of *N*_ε_-Boc-Lys NDIs from the group’s previous work that showed opposing interaction with c-KIT2 [6]. (**b**) The scope of NDIs explored in this work. In each compound, the NDI is disubstituted with the same β-amino acid functionality. For brevity, **(*2S,3R*)-1** is referred to as **1** throughout the text.

NDIs are an attractive class of ligands partly because of their chemical accessibility as well as their fulfilment of the general criteria for G4 ligands having an electron-deficient polyaromatic core surface with potential for functionalization. In this study, we have selected several β-amino acids which are analogous to ones within the biologically relevant α-amino acid series. Amino acids provide water solubility and specificity with G4 DNA via backbone interaction. Previous work in our group has suggested that the length of the peripheral substituents dictates binding behaviour [31]. The NDIs used in this study are highlighted in Figure 2.

This study focused on both the interaction from the peripheral substituents, as well as the importance of chirality in the G4 DNA–NDI interaction. We synthesised three sets of enantiomers, **3**, **4**, **5**, along with two other chiral amino acid NDIs to probe this interaction. The synthetic route followed a one-step microwave-assisted reaction for all derivatives [7,32].

All experiments were performed in phosphate-buffered saline (PBS, 10 mM K^+^) comprising 100 mM potassium fluoride, pH 7.4, to resemble physical conditions. The K^+^ concentration provides the fundamental cation for G-quadruplex formation. The concentration of the NDI in solution was determined using the characteristic absorbance peaks at ca. 362 nm and 383 nm.

### 2.2. G-Quadruplex Topology Analysis

It is well-known that G4s form different conformations depending on the identity of the monovalent cation and the conditions of annealing [33]. Understanding this topology is essential to deciphering the NDI interaction with the G4s. Circular dichroism (CD) provides a reflection of the structural elements within the G4 in the form of the backbone [34]. The CD signatures of the sequences used in this study are in agreement with previously reported information [6,35,36]. h-TELO’s CD profile in potassium-PBS is typical of a hybrid folded sequence, with its fingerprint characterised by a positive Cotton effect at ca. 292 nm and a small negative Cotton effect at ca. 255 nm, with a shoulder at 273 nm which is indicative of a [3 + 1] hybrid arrangement. This result is corroborated by previously published work [37,38]. TBA is known to form an antiparallel sequence [36,39,40], with CD fingerprint positive peaks at ca. 290 nm, and a negative peak at ca. 267 nm. c-KIT1 possesses spectroscopic features characteristic of a parallel sequence with a strong positive peak at ca. 262 nm and a negative peak at ca. 240 nm; however, it also displayed a shoulder at ca. 290 nm. This indicated the presence of a small proportion of a hybrid quadruplex structure (ca. 3:1 parallel:hybrid) under the experimental conditions, which is in agreement with previously reported data [35]. The control sequence for this work was a duplex DNA (dsDNA), characterised by a small positive peak at ca. 275 nm and a large negative peak at ca. 250 nm. Whilst topology changing ligands have previously been observed [33,41,42], the NDIs employed in this study did not affect the topology of the quadruplexes upon addition, as indicated by identical CD spectra pre- and post-NDI addition but also in pre- and post-variable temperature studies scans.

### 2.3. Circular Dichroism Variable Temperature Studies

The effect of the NDIs on the stability of the G4s and dsDNA was assessed using VT-CD. Circular dichroism provides a fingerprint of the folded topology of DNA. As the temperature increases, the DNA denatures, or “melts”, leading to a loss of the CD signal. The shape of the “melting curve” provides information on the progress of DNA denaturation. Plotting the change in ellipticity as a function of temperature allows for calculation of the melting temperature (T_m_, reported here in °C), defined as the temperature at which 50% of the DNA is folded and 50% unfolded. The change in the melting temperature (ΔT_m_) reflects the stabilizing/destabilizing effects of the NDI on the G4. A positive ΔT_m_ indicates that the NDI stabilised the G4. The T_m_ of the G4-NDI complex is dependent on multiple factors: affinity for each NDI for a particular sequence topology, binding nature, side-chain functionality on the NDIs, and the number of available binding sites. It is known that DNA can unfold either via an isosbestic point through a single denaturation step, or via a concerted process with intermediates observed.

Each VT-CD experiment was conducted with five equivalents of NDI to G4 DNA. The CD was acquired sequentially across a temperature range of 15–95 °C. In order to prevent the NDI aggregation in aqueous solution, the NDI stock solutions were prepared at 150 μM. The concentration of NDI in the NDI/G4-DNA studies ranged between 10 and 25 μM. The melting profiles of the G4-DNAs were monitored at selected wavelengths of maximum ellipticity, rather than the full-spectrum, allowing for a greater accumulation of data points in a shorter timeframe compared to measuring across the entire wavelength range. The wavelengths were selected from a full-spectrum scan across 200–450 nm, with the wavelengths of maximum ellipticity (θ_max_) of the DNA were chosen.

The data were fitted using the Boltzmann equation, where the T_m_ is the midpoint of the sigmoidal plot. The results here are discussed in terms of ΔT_m_ for each respective G4-DNA. The ΔT_m_ comparison allows for evaluation of enantioselective behaviour between the NDIs and G4s, as well as the effect of changing from an α-amino acid to a β-amino acid. Previous work within the group has shown a dependence on chain length for the interaction and stabilization of G4s. This work further explores this dependence.

### 2.4. GNINA Modelling

To explore differences in stabilization between enantiomers, docking studies using empirical scoring functions through GNINA was performed [43]. GNINA optimises the shape, binding position, and orientation of the ligand (NDI) relative to a static DNA model. The results are based upon a default scoring function in GNINA’s convolutional neural network (CNN) system. The compilation and training of the CNN datasets is defined in Koes and co-workers’ other works [44]. The NDI models are then ranked based on their relative “score” defined by the CNN with regards to the root mean square deviation (RMSD) of known binding poses and their binding affinity.

The NDI structures were energy optimised in Avogadro using UFF with conjugate gradients, followed by further optimisation in MOPAC [45], (PM7 COSMO water model) before a final DFT (cam-B3Lyp/Def2TZVP/SMD(water)) [46,47,48,49] energy minimisation. The DNA sequence structures were selected from the RCSB Protein Databank (PDB) via sequence similarity to the sequences used in this study (see more in Section 3.1) [50]. GNINA was set up to output the top 18 structures for each NDI, each optimisation starting from a random seed. The models for each NDI/G4 pair were then compared in terms of ranking scores and structural characteristics of the interaction. Where multiple NDI cores coalesced into a similar position, the position was selected as being a favourable binding position for the NDI.

The different outputs for the NDI enantiomers when interacting with the same G4 provided a starting point for rationalizing the enantioselective behaviour observed experimentally. For each G4-DNA, the GNINA models are discussed where appropriate.

### 2.5. NDI-DNA Interactions

#### 2.5.1. c-KIT1

Work with c-KIT1 at 5 μM found that the DNA did not completely unfold during the VT experiment. Consequently, the c-KIT1 studies were performed at 2 μM with 5 equivalents of the NDI.

All the NDIs (except **(*R*)-4**) have a stabilizing effect on the c-KIT1 quadruplex, as indicated by increases in the T_m_ (Table 1). The greatest stabilizing interactions have been observed for the interaction with **(*S*)-2** and **(*S*)-5**. The increased stabilization with **(*S*)-2** and **(*S*)-5** point to a preferential binding of c-KIT1 with the (*S*)-NDIs. The three-dimensional conformation of the (*S*)-enantiomers appears to have a more favourable spatial arrangement, which is more conducive to binding with the parallel c-KIT1 conformation compared to the (*R*)-enantiomers.

**Figure 3:**
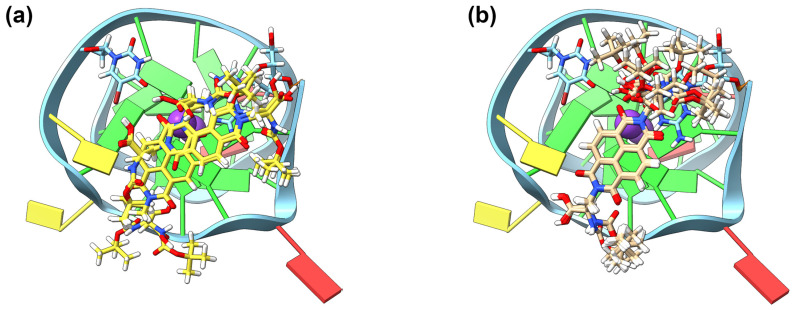
c-KIT1 modelled with GNINA showing binding with (**a**) eight overlaid models of **(*S*)-5** and (**b**) two different stacking positions **(*R*)-5** with two models each.

The difference in ΔT_m_ between the two enantiomers of the α-boc-aminoalanine NDI (**5**) is noteworthy: a ΔΔT_m_ of 3.7 °C. The GNINA modelling for this pair showed a reflection of these data in the number of models that converged at a similar location (see Figure 3). For the **(*S*)-5** NDI, eight of out the top eighteen NDI docking models were overlaid with almost exact retention of the core position and similar position of the side chains; whilst for the (*R*)-enantiomer, only two pairs out of the top eighteen docking models were in a similar geometry when interacting with the c-KIT1 model. We rationalise this as indicating that the point chirality of the α-boc-aminoalanine sidechains is driving the interaction with the G-qaudruplex backbone which, in turn, influences the overall interaction between the chiral DNA and the chiral ligand, with a marked energetic preference for the interaction with the (*S*)-enantiomer.

Additionally, the ΔT_m_ value for the interaction of c-KIT1 with **(*S*)-2** is of interest due to its increased value when compared with the rest of the ligands. This NDI contains a hydroxy group in the β-position with respect to the NDI core, which can act as a hydrogen bond donor and/or acceptor when interacting with the sugars and the phosphate groups of the backbone. These interactions seem to be important, as highlighted by the increased ΔT_m_.

#### 2.5.2. h-TELO

**Figure 4:**
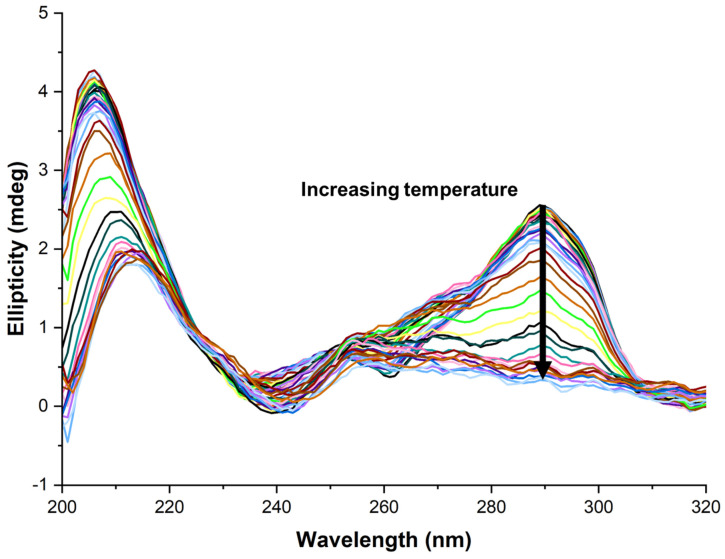
A full-spectrum VT-CD of h-TELO with **(*S*)-3** showing a decay of the characteristic hybrid peak at 290 nm with increasing temperature, whilst showing no growth in any other characteristic peak areas. Each different colour line represents a different temperature point, incrementing at 2.5 °C.

Previous work in the Pantos group [33], as well as others in the literature [41,42], have shown the ability of ligands to change the conformation of h-TELO during the course of a VT study. Therefore, a selection of NDIs was tested with h-TELO in a full-spectrum VT-CD scan to see whether the NDIs induced a conformational change in the G4. There was no apparent change in the CD, indicating that there was no change in the conformation of h-TELO during the VT study. This was further analysed using software developed by Chaires et al., which showed that there was no significant deviation from hybrid conformation [51]. An exemplar full-spectrum VT-CD is shown in Figure 4, showing no conformational change in the h-TELO.

Limited enantioselective binding behaviour was observed between the β-homoleucine (**4**) and α-boc-aminoalanine (**5**) NDIs and h-TELO (Table 2). Interestingly, the (*R*)-enantiomer for both **4** and **5**, i.e., both **(*R*)-4** and **(*R*)-5**, stabilise h-TELO quadruplex less than the (*S*)-enantiomers. This points to a reduced compatibility between the 3D-structure of the (*R*)-enantiomers with the hybrid h-TELO quadruplex structure compared to the (*S*)-enantiomers. This kind of selective chiral recognition of quadruplexes was previously reported by us in the case of NDI derivatives bearing lysine sidechains [6].

**Figure 5:**
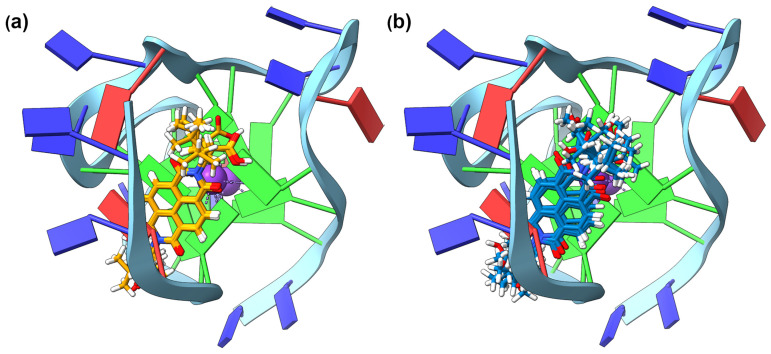
h-TELO modelled with GNINA showing binding with (**a**) seven overlaid models of **(*R*)-4** and (**b**) four overlaid models of **(*S*)-4**.

The chiral recognition with the β-homoleucine (**4**) derivatives was explored further using GNINA modelling. A favourable binding position with **(*S*)-4** was seen, with seven of the top eighteen models in a highly conserved core position above the top G-tetrad and intercalating below a thymine nucleobase (Figure 5a). The diimide nitrogen and carboxylic end of the side chain are retained above the central cation channel of the G4, potentially driven by the anionic nature of the carboxylic acid group at physiological pH. For **(*R*)-4**, there are only four models with a conserved NDI core position, albeit in the same binding position as the (*S*)-enantiomer (Figure 5b). The lack of disparity in the binding position can help to explain why the ΔΔT_m_ is only 1.2 °C, compared to 3.7 °C for **(*R*)-5** and **(*S*)-5** with c-KIT1.

#### 2.5.3. TBA

Thrombin binding aptamer (TBA) is an antiparallel sequence that is an aptamer for thrombin, which is known to take part in the pathogenesis of several lung cancer and fibrosis amongst other diseases [52,53]. To assess whether the antiparallel conformation would be retained during VT-CD, a full-spectrum VT-CD study was run with TBA alone and with the α-boc-aminoalanine derivatives. The data were analysed using the software developed by Chaires et al., which showed that the conformation did not change during these experiments [51].

**Figure 6:**
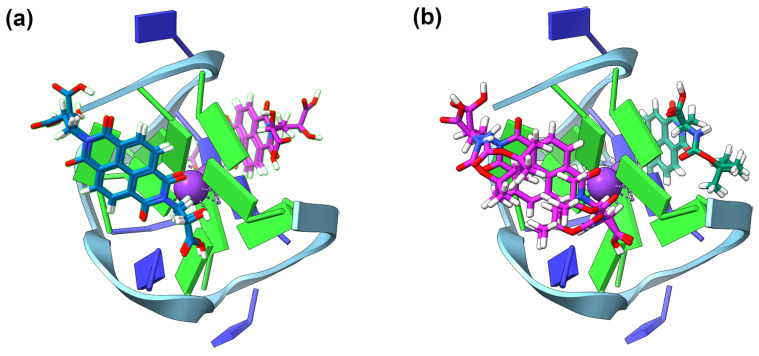
The output of GNINA modelling of TBA with **(*S*)-5** (**a**) and **(*S*)-2** (**b**), demonstrating the lack of coalescence of the NDI core binding position. In each instance, there are two models at each of the three binding positions.

TBA showed very limited interaction with the NDIs (Table 3). For the most part, the compounds neither stabilised nor destabilised the TBA. This would seem to suggest that the β-amino acid NDIs do not have structure conducive to interaction with the antiparallel sequence. The GNINA modelling reflects this lack of interaction. For all the G4-NDI complexes, there are several different binding positions which all have varying degrees of NDI core retention (Figure 6). For most of the NDIs, there are three different binding positions with four or fewer models which have a highly retained NDI core position. Poor convergence on a binding conformation highlights that there are no particularly favourable interactions between any of the NDIs and TBA.

#### 2.5.4. dsDNA

We employed a short sequence of dsDNA, which acts as a model for all duplex DNA, to study its interaction with our compounds. From the previous work, it was known that the NDIs did not induce a conformational change in the dsDNA.

The NDIs showed a tendency to slightly destabilise the dsDNA (Table 4). This is in contrast with the α-amino acid NDI series that showed little-to-no stabilising interaction with the dsDNA [6,7]. Whilst the interaction is destabilizing, it is not a significant destabilization when compared with the c-KIT1-NDI complex stabilization values.

**Figure 7:**
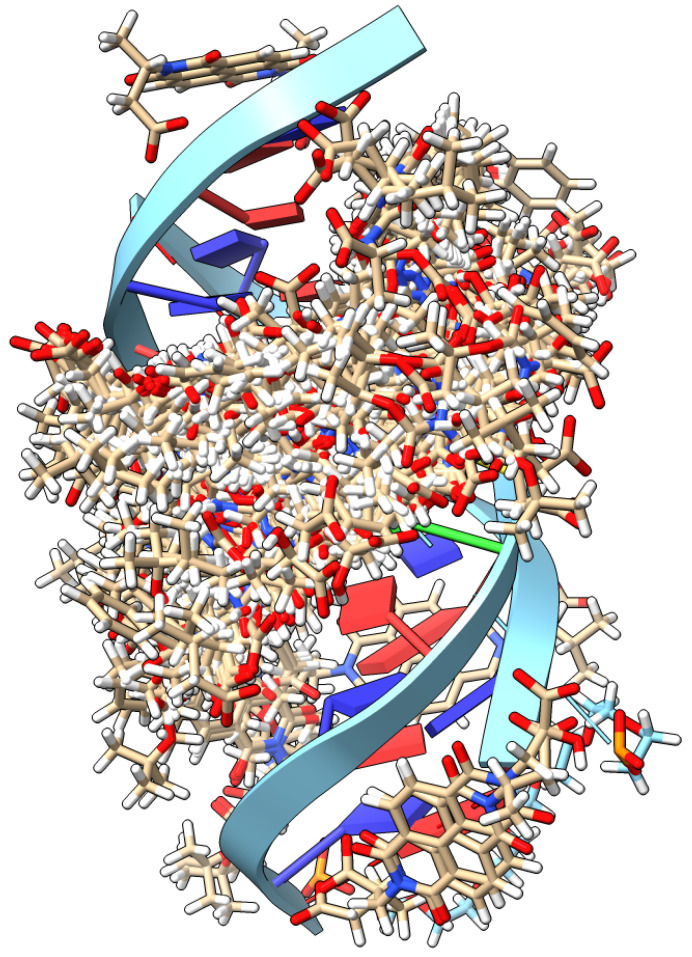
dsDNA modelling run with GNINA showing the array of binding positions for all the NDIs around the helical groove, with no convergence on a favoured binding site.

The GNINA docking studies used a dsDNA structure based on the purchased sequence followed by DFT energy optimization (for details, see Materials and Methods). The modelling with this sequence showed a lack of convergence for the NDI cores as they are bound in a general manner into the major helical groove (Figure 7), with a number of them binding onto the exposed bottom face. The modelling is in agreement with the VT-CD data that there are few-to-no favourable binding sites/poses for the NDIs with dsDNA. The lack of a defined binding site can also explain the low K_D_ values obtained for the interaction between dsDNA and the NDI **4** and **5** enantiomeric pairs (*vide infra*).

### 2.6. NDI-DNA Titration Studies

The binding affinities for the NDIs with the G4 DNAs were assessed via UV-Vis titrations. We decided to pick the NDI-G4 pairs which showed the largest influence on the DNA T_m_ and the biggest differences between enantiomers in binding (c-KIT1 with **(*R*)-5** and **(*S*)-5**; and h-TELO with **(*R*)-4** and **(*S*)-4**). Due to the weak interaction observed via VT-CD between TBA and the NDIs, these G4-NDI complexes were not probed in UV-Vis titrations. Binding was assessed by observing a bathochromic shift in UV-Vis absorbance upon increasing addition of the NDI to the DNA. The spectroscopic data were fitted with a one-to-one binding mathematical model that allowed us to estimate the strength of the interaction. We have monitored the CD spectrum during the titration to observe if the conformation of the G4 is maintained upon increasing equivalents of NDI. The K_D_ values are displayed in Table 5.

c-KIT1 titrations were performed using **(*R*)-5** and **(*S*)-5** using 2 μM c-KIT1 with a stock solution of ligand with DNA at 30 μM of ligand. This allowed for exploration of a wide range of equivalence ratios up to ca. 6.5 equivalents of ligand to DNA.

h-TELO titrations were performed using **(*R*)-4** and **(*S*)-4** using 5 μM h-TELO with a stock solution of ligand with DNA at 75 μM of ligand. Once again, this allowed for exploration up to ca. 6.5 equivalents of NDI to DNA.

As a general rule, the effective range for a G4-binder is generally considered to be K_D_ < 10 μM [54,55]. For both **(*R*)-5** and **(*S*)-5**, the K_D_ value is within this bracket; so, from this metric, each could be considered an effective G4-binder. The **(*S*)-5** derivative has an association constant ca. six times higher than its enantiomer **(*R*)-5**. This aligns with what the VT-CD data showed and helps to confirm that **(*S*)-5** is a much more effective G4-ligand. NDI **(*S*)-4** is just above the reasonable range but within the error for a G4-binder, whereas **(*R*)-4** is above this range, including the error range. When compared with literature data, the K_D_ values are about an order of magnitude larger than those of Telomestatin^®^ and BRACO-19 [56,57].

As with **5**, the titration data demonstrate enantioselective binding behaviour between h-TELO and **4**. This also aligns with the VT-CD data showing a destabilizing interaction between **(*R*)-4** and h-TELO, whilst **(*S*)-4** shows a stabilizing interaction. The titration data are thus further evidence of chiral recognition between the chiral NDIs and the G4s. As control, we have studied the interaction between these enantiomeric pairs and dsDNA. In each case, the K_D_ values were significantly larger than the ones measured for the interaction between the NDIs and G4-DNA, thus indicating a level of selectivity in favour of the interaction of NDIs **4** and **5** and G4-DNA vs. dsDNA.

## 3. Materials and Methods

The oligonucleotides used in this study were purchased from Invitrogen^®^ (Waltham, MA, USA), with the following sequences (from 5′ to 3′):

- c-KIT1: GGG AGG GCG CTG GGA GGA GGG;
- h-TELO: AGG GTT AGG GTT AGG GTT AGG GT;
- TBA: GGT TGG TGT GGT TGG;
- dsDNA: TAT AGC TAT A HEG T ATA GCT ATA.

The sequences were used as received without further purification.

The oligonucleotide solutions were annealed in pH 7.4 phosphate-buffered saline solution (PBS) containing KH_2_PO_4_/K_2_HPO_4_ (10 mM) and potassium fluoride (KF, 100 mM). The oligonucleotide solutions were annealed via placement in a dry-block heater at 95 °C for exactly 5 min, before being allowed to cool to room temperature and then stored at 4 °C for at least 24 h before use. The samples were annealed to 20 μM, and the exact concentration of each oligonucleotide after annealing (and corresponding diluted batches) was determined by measuring the absorbance for the peak corresponding to λ_max_. The extinction coefficients were provided by the manufacturer. The following values were used (given in L mol^−1^ cm^−1^): c-KIT1: 213,000, h-TELO: 237,000, TBA: 133,200, dsDNA: 6600. For each study, the solutions were diluted to the desired concentration (2 or 5 μM) and used without further purification. The concentration of the DNA sequence used in each experiment is given in the Supplementary Materials (Figures S18–S21).

Ultrapure water from a Milli-Q^®^ water system at 18.2 Ω cm^−1^ was used in all CD experiments. All other reagents and solvents were supplied by either Sigma-Aldrich (Gillingham, UK), VWR (Lutterworth, UK), Fluorochem (Glossop, UK), or TCI (Oxford, UK) and used as received. Microwave-assisted reactions were conducted in a CEM^®^ Focused Microwave^™^ Discover microwave reactor. Samples for NMR analyses were prepared used dimethylsulfoxide (DMSO-*d*_6_). The ^1^H and ^13^C NMR were acquired at 400 and 101 MHz, respectively, using a Bruker Neo spectrometer (Billerica, MA, USA). The data were recorded at 300 K, and the spectra referenced to the residual solvent peak. Coupling constants are reported in Hertz (Hz) and signal multiplicity is denoted as broad (br), singlet (s), doublet (d), doublet of doublets (dd), doublet of doublets of doublets (ddd), triplet (t), quartet (q), and multiplet (m). Chemical shifts are reported in parts per million (ppm). The electrospray ionisation quadrupole time-of-flight (QTOF-ESI) mass spectrometry (positive ion) were recorded on an Agilent Technologies 6545 QTOF LC-MS instrument (Santa Clara, CA, USA). HPLC data were acquired using an Agilent 1100 HPLC system equipped with a quaternary pump and a 1260 Diode Array Detector. Elemental analysis was acquired using the Elemental Analysis Service at London Metropolitan University.

The ^1^H and ^13^C NMR and MS spectra of all naphthalenediimide compounds are provided for completeness in the Supplementary Materials (Figures S2–S17).

### 3.1. Naphthalenediimide Synthesis

#### General Synthetic Procedure

1,4,5,8-Naphthalenetetracarboxylic acid dianhydride (NDA, 1 equiv) was dissolved in 6 mL dry DMF in a 10 mL oven-dried microwave tube and purged with N_2_. Triethylamine (>5 equiv, excess) and the β-amino acid (2 equiv) was added, and the suspension was homogenised and sonicated for 45 min. The reaction was heated at 120 °C under microwave irradiation in a CEM^®^ Focused Microwave™ Synthesis System, Model Discover for 10 min. The resulting solution was cooled, and the solvent was removed under reduced pressure. The crude product was resuspended in the minimum amount of acetone (ca. 5 mL) and added dropwise to 0.2 M HCl_(aq)_. The precipitate that formed was filtered, washed with water, and dried in vacuo, yielding an off-white/tan product.

Synthesis of **1**: The reaction was performed on 1 equivalent of NDA (200 mg, 0.746 mmol) and 2.3 equivalents of (2*S*,3*R*)-3-amino-2-hydroxy-4-phenylbutanoic acid (334.8 mg, 1.72 mmol) using the general procedure. Yield: 392.3 mg, 85%. ^1^H NMR (400 MHz, DMSO-*d*_6_): δ 12.97 (br s, 2H), 8.61 (m, 4H), 7.04 (s, 10 H), 5.79 (br s, 2H), 5.57 (td, *J*_(H,H)_ = 10, 5 Hz, 2H), 4.80 (d, *J*_(H,H)_ = 10 Hz, 2H), 3.48 (dd, *J*_(H,H)_ = 14, 11 Hz, 2H), 3.04 (dd, *J*_(H,H)_ = 14, 5 Hz, 2H. ^13^C NMR (101 MHz, DMSO-*d*_6_): δ 174.03, 163.50, 129.12, 128.66, 126.79, 126.29, 70.61, 57.30, 34.20. Q-TOF–ESI: *m/z* calcd for C_34_H_27_N_2_O_10_ (M + H)^+^: 623.1587, found: 623.1666. Melting point: decomposes at 272–278 °C. FTIR (ATR, cm^−1^): 3429 (hydrogen-bonded O-H), 3030 (carboxylic acid O-H), 2967, 2930 (sp^3^ C-H), 1702 (carboxylic acid C=O), 1653 (imide C=O), 1580 (aryl C=C). Anal. Calcd for C_34_H_26_N_2_O_10_∙1.33H_2_O: C, 62.76; H, 4.47; N, 4.33; found: C, 62.76; H, 4.06; N, 4.18.

Synthesis of **(*S*)-2**: The reaction was performed on 1 equivalent of NDA (200 mg, 0.746 mmol) and 2.3 equivalents of (*S*)-3-amino-2-hydroxypropanoic acid (180.3 mg, 1.72 mmol) using the general procedure. Yield: 245.5 mg, 74%. ^1^H NMR (400 MHz, DMSO-*d*_6_): δ 12.67 (br s, 2H), 8.69 (s, 4H), 5.60 (s, 2H), 4.32 (m, 6H). ^13^C NMR (101 MHz, DMSO-*d*_6_): δ172.52, 162.90, 130.46, 126.48, 126.05, 45.45, 37.87, 17.71. Q-TOF–ESI: *m/z* calcd for C_20_H_15_N_2_O_10_ (M + H)^+^: 443.0648, found: 443.0731. Melting point: decomposes at 285–290 °C. FTIR (ATR, cm^−1^): 3416 (O-H), 3061 (CH), 2923 (C-H), 1704 (carboxylic acid C=O), 1652 (imide C=O), 1578 (aryl C=C). Anal. Calcd for C_20_H_14_N_2_O_10_∙H_2_O: C, 52.18; H, 3.5; N, 6.09; found: C, 52.26; H, 3.14; N, 5.79.

Synthesis of **(*R*)-3**: The reaction was performed on 1 equivalent of NDA (200 mg, 0.746 mmol) and 2.2 equivalents of (*R*)-3-aminobutanoic acid (169.2 mg, 1.61 mmol) using the general procedure. Yield: 262.3 mg, 80%. ^1^H NMR (400 MHz, DMSO-*d*_6_): δ 12.24 (br s, 2H), 8.67 (s, 2H), 5.53 (td, *J*_(H,H)_ = 7, 7 Hz, 2H), 3.08 (dd, *J*_(H,H)_ = 16, 7 Hz, 2H), 2.98 (dd, *J*_(H,H)_ = 16, 7 Hz, 2H), 1.52 (d, *J*_(H,H)_ = 7 Hz, 6H). ^13^C NMR (101 MHz, DMSO-*d*_6_): δ 172.51, 162.92, 130.45, 126.50, 126.08, 45.42, 37.86, 17.70. Q-TOF ESI: *m/z* calcd for C_22_H_19_N_2_O_8_ (M + H)^+^: 439.1063, found: 439.1139. Melting point: decomposes at 280–285 °C. FTIR (ATR, cm^−1^): 3327 (O-H), 2975 (C-H), 1703 (carboxylic acid C=O), 1656 (imide C=O), 1578 (aryl C=C). Anal. Calcd for C_22_H_18_N_2_O_8_: C, 60.28; H, 4.14; N, 6.39; found: C, 60.36; H, 3.76; N, 6.04.

Synthesis of **(*S*)-3**: The reaction was performed on 1 equivalent of NDA (200 mg, 0.746 mmol) and 2.2 equivalents of (*S*)-3-aminobutanoic acid (169.2 mg, 1.61 mmol) using the general procedure. Yield: 237.3 mg, 73%. ^1^H NMR (400 MHz, DMSO-*d*_6_): δ 12.24 (br s, 2H), 8.67 (s, 2H), 5.53 (td, *J*_(H,H)_ = 7, 7 Hz, 2H), 3.08 (dd, *J*_(H,H)_ = 16, 7 Hz, 2H), 2.98 (dd, *J*_(H,H)_ = 16, 7 Hz, 2H), 1.52 (d, *J*_(H,H)_ = 7 Hz, 6H). ^13^C NMR (101 MHz, DMSO-*d*_6_): δ 172.51, 162.92, 130.45, 126.50, 126.08, 45.42, 37.86, 17.70. Q-TOF ESI: *m/z* calcd for C_22_H_19_N_2_O_8_ (M + H)^+^: 439.1063, found: 439.1144. Melting point: decomposes at 280–285 °C. FTIR (ATR, cm^−1^): 3327 (O-H), 2974 (C-H), 1699 (carboxylic acid C=O), 1649 (imide C=O), 1579 (aryl C=C). Anal. Calcd for C_22_H_18_N_2_O_8_∙0.33H_2_O: C, 59.59; H, 4.12; N, 6.24; found: C, 59.47; H, 4.23; N, 6.30.

Synthesis of **(*R*)-4**: The reaction was performed on 1 equivalent of NDA (200 mg, 0.746 mmol) and 2.2 equivalents (*R*)-3-amino-5-methylhexanoic acid (238.2 mg, 1.61 mmol) using the general procedure. Yield: 280.0 mg, 72%. ^1^H NMR (400 MHz, DMSO-*d*_6_): δ 12.22 (br s, 2H), 8.69 (s, 4H), 5.55 (m, 2H), 3.07 (dd, *J*_(H,H)_ = 16, 7 Hz, 2H), 2.91 (dd, *J*_(H,H)_ = 16, 7 Hz, 2H), 2.18 (ddd, *J*_(H,H)_ = 14, 9, 5 Hz, 2H), 1.59 (ddd, *J*_(H,H)_ = 14, 9, 5 Hz, 2H), 1.51 (d, *J*_(H,H)_ = 14 Hz, 2H), 0.92 (d, *J*_(H,H)_ = 6 Hz, 3H), 0.85 (d, *J*_(H,H)_ = 6 Hz, 3H). ^13^C NMR (101 MHz, DMSO-*d*_6_): δ 172.99, 126.68, 48.72, 46.06, 41.15, 37.81, 25.20, 23.54, 22.57. Q-TOF ESI: *m/z* calcd for C_28_H_31_N_2_O_8_ (M + H)^+^: 523.2002, found: 523.2081. Melting point: decomposes at 139–143 °C. FTIR (ATR, cm^−1^): 3219 (O-H), 2955 (C-H), 1703 (carboxylic acid C=O), 1659 (imide C=O), 1579 (aryl C=C). Anal. Calcd for C_28_H_30_N_2_O_8_∙0.33H_2_O: C, 63.59; H, 5.85; N, 5.3; found: C, 63.45; H, 5.7; N, 5.15.

Synthesis of **(*S*)-4**: The reaction was performed on 1 equivalent of NDA (200 mg, 0.746 mmol) and 2.2 equivalents of (*S*)-3-amino-5-methylhexanoic acid (238.2 mg, 1.61 mmol) using the general procedure. Yield: 285.0 mg, 73%. ^1^H NMR (400 MHz, DMSO-*d*_6_): δ 12.22 (br s, 2H), 8.69 (s, 4H), 5.55 (m, 2H), 3.07 (dd, *J*_(H,H)_ = 16, 7 Hz, 2H), 2.91 (dd, *J*_(H,H)_ = 16, 7 Hz, 2H), 2.18 (ddd, *J*_(H,H)_ = 14, 9, 5 Hz, 2H), 1.59 (ddd, *J*_(H,H)_ = 14, 9, 5 Hz, 2H), 1.51 (d, *J*_(H,H)_ = 14 Hz, 2H), 0.92 (d, *J*_(H,H)_ = 6 Hz, 3H), 0.85 (d, *J*_(H,H)_ = 6 Hz, 3H). ^13^C NMR (101 MHz, DMSO-*d*_6_): δ 172.99, 126.68, 48.72, 46.06, 41.15, 37.81, 25.20, 23.54, 22.57. Q-TOF ESI: *m/z* calcd for C_28_H_31_N_2_O_8_ (M + H)^+^: 523.2002, found: 523.2077. Melting point: decomposes at 140–144 °C. FTIR (ATR, cm^−1^): 3217 (O-H), 2955, 2872 (C-H), 1703 (carboxylic acid C=O), 1660 (imide C=O), 1579 (aryl C=C). Anal. Calcd for C_28_H_30_N_2_O_8_∙0.25H_2_O: C, 63.83; H, 5.83; N, 5.32; found: C, 63.66; H, 5.66; N, 5.30.

Synthesis of **(*R*)-5**: The reaction was performed on 1 equivalent of NDA (200 mg, 0.746 mmol) and 2.3 equivalents of (*R*)-3-amino-2-((*tert*-butoxycarbonyl)amino)propanoic acid (350.3 mg, 1.72 mmol) using the general procedure. Yield: 477.1 mg, 99%. ^1^H NMR (400 MHz, DMSO-*d*_6_): δ 12.91 (br s, 2H), 8.77 (s, 4H), 7.21 (t, *J*_(H,H)_ = 4 Hz, 2H), 4.41 (d, *J*_(H,H)_ = 3 Hz, 6H), 1.15 (m, 18H). ^13^C NMR (101 MHz, DMSO-*d*_6_): δ 172.06, 163.20, 155.82, 131.06, 126.72, 126.65, 78.66, 51.22, 28.34. Q-TOF ESI: *m/z* calcd for C_30_H_32_N_4_O_12_Na (M + Na)^+^: 663.2017; found 663.1911. Melting point: 218–225 °C. FTIR (ATR, cm^−1^): 3414 (N-H/O-H), 2981, 2932 (C-H), 1704 (carboxylic acid C=O), 1664 (imide C=O), 1581 (aryl C=C). Anal. Calcd for C_30_H_32_N_4_O_12_∙1.7H_2_O: C, 53.68; H, 5.32; N, 8.35; found: C, 53.43; H, 4.93; N, 8.24.

Synthesis of **(*S*)-5**: The reaction was performed on 1 equivalent of NDA (200 mg, 0.746 mmol) and 2.3 equivalents of (*S*)-3-amino-2-((*tert*-butoxycarbonyl)amino)propanoic acid (350.3 mg, 1.72 mmol) using the general procedure. Yield: 455.2 mg, 95%. ^1^H NMR (400 MHz, DMSO-*d*_6_): δ 12.91 (br s, 2H), 8.77 (s, 4H), 7.21 (t, *J*_(H,H)_ = 4 Hz, 2H), 4.41 (d, *J*_(H,H)_ = 3 Hz, 6H), 1.15 (m, 18H). ^13^C NMR (101 MHz, DMSO-*d*_6_): δ 172.06, 163.20, 155.82, 131.06, 126.72, 126.65, 78.66, 51.22, 28.34. Q-TOF ESI: *m/z* calcd for C_30_H_32_N_4_O_12_Na (M + Na)^+^: 663.2017; found 663.1912. Melting point: 220–225 °C. FTIR (ATR, cm^−1^): 3414 (N-H/O-H), 2973, 2881 (C-H), 1704 (carboxylic acid C=O), 1664 (imide C=O), 1581 (aryl C=C). Anal. Calcd for C_30_H_32_N_4_O_12_∙0.4Na_2_CO_3_: C, 53.46; H, 4.72; N, 8.20; found: C, 53.48; H, 4.62; N, 8.24.

### 3.2. Variable-Temperature CD Studies

CD and UV-Vis experiments were performed in an Applied Photophysics Chirascan Circular Dichroism Spectrophotometer (Leatherhead, UK) equipped with a Peltier temperature controller. The following parameters were used for full spectra measurements: wavelength scanning range: 200–450 nm; temperature: 15 °C; scanning increments: 1 nm; monochromator bandwidth: 2.0 nm; sampling time-per-point: 1.5 s; pathlength cuvette: 2 mm.

In all variable-temperature (VT) studies, the NDI (5 equivalents) was added to the DNA sequence solution previously annealed. For VT experiments, a multiple-wavelength spectrum was recorded using the wavelengths of maximum ellipticity (λ_max_) with the following parameters: temperature range: 15–95 °C (with 1 °C increments); temperature step: 1 °C; temperature equilibration time: 30 °C; monochromator bandwidth: 2.0 nm; sampling time-per-point: 1.5 s. The VT-CD data were processed using OriginPro^®^ 2021b (Northhampton, MA, USA) and fitted to the Boltzmann equation (the spectra are given in the Supplementary Materials (Figures S18–S21)).

### 3.3. Titration Studies

Titration studies were performed utilising a JASCO J-810 Spectropolarimeter (Tokyo, Japan) equipped with a Peltier temperature controller or a Perkin-Elmer Lambda 35 UV/Vis Spectrophotometer (Waltham, MA, USA). In all studies, the same DNA concentration was used as for the VT-CD studies (2 or 5 μM). A stock solution was generated with identical DNA concentration and 15 equivalents of the corresponding NDI. The following parameters were used for the full-spectrum scans: wavelength scanning range: 200–450 nm; temperature: ca. 20 °C; scanning increments: 1 nm; monochromator bandwidth: 2 nm; sampling time-per-point: ca. 0.5 s; pathlength cuvette: 10 mm. The titration was performed by increasing addition of the stock solution to the oligonucleotide solution, with a full-spectrum scan at each interval.

Titrations were fitted using a simple one-to-one binding mathematical model corresponding to the change in the absorbance at the DNA and NDI λ_max_ values. The fitting curves are outlined in the Supplementary Materials (Figures S22–S29).

### 3.4. GNINA Modelling

The structures of the NDI ligands were minimised in Avogadro v1.2.0 using Universal Force Field (UFF) with conjugate gradients. The UFF minimised geometries were then further optimised using MOPAC [45], PM7, followed by DFT cam-B3Lyp/Def2-TZVP/SMD(water) [46,47,48,49].

The NDI models were docked using GNINA with sequences corresponding the greatest similarity to those used empirically (for comparison, see Table 6) [43]: c-KIT1 (PDB ID: 3QXR), TBA (PDB ID: 1C35), hybrid (PDB ID: 5MVB). dsDNA were generated from the purchased sequence using the DNA Sequence to Structure tool provided by IIT Delhi [58], followed by energy optimisation with Orca [59] DFT HF-3C/SMD(water).

The grid box was pre-defined to encompass the whole quadruplex with an extra 8 Å added to avoid any ligand clipping. The best convolution neural network (CNN) pose scores with significantly different binding positions were used, hydrogen atoms were added in a standard geometry Avogadro v.1.2.0, and the combined docked structures visualised in Chimera X v.1.6.1.

### 3.5. HPLC Methodology

Column: Phenomenex Prodigy C_8_, 150 × 4.6 mm, 5 µM; injection volume: 2 or 5 µL; temperature: 30 °C; run time: 30 min; elution profile: see Table S2.

## 4. Conclusions

**Figure 8:**
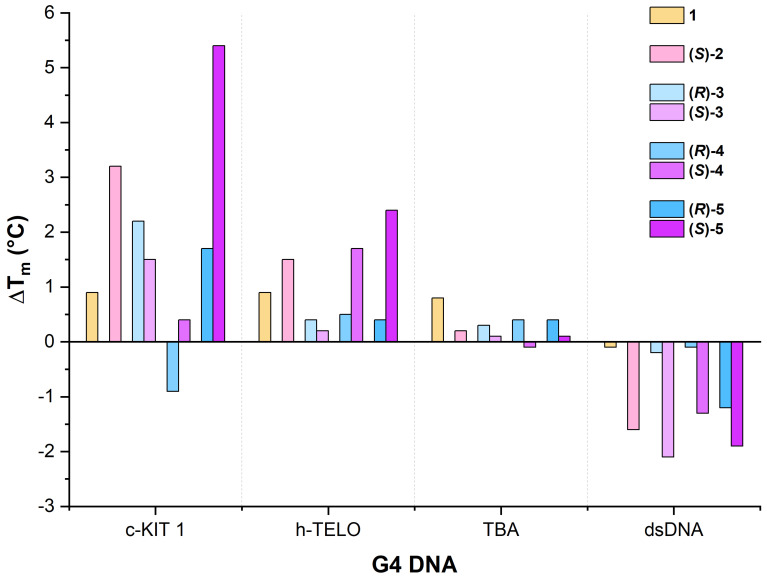
The T_m_ comparison for all the G4 DNAs with each of the ligands. The values represent the ΔT_m_ values for each NDI-G4 mixture relative to the DNA by itself. In every experiment, the NDI was run at five equivalents relative to the DNA concentration. Each DNA concentration is listed in Section 3. The individual T_m_ plots with standard errors are detailed in the Supplementary Materials (Figures S22–S25).

For this study we have synthesised eight β-amino acid-derived NDIs, including three enantiomeric pairs. The screening studies with three different G4-forming sequences (c-KIT1, h-TELO, and TBA), along with the control dsDNA, demonstrated the general preference for the stabilization of the parallel (c-KIT1) over the hybrid (h-TELO) and antiparallel (TBA) G-quadruplexes. A full comparison of the data is highlighted in Figure 8.

Enantioselective behaviour was observed in the binding between the α-boc-aminoalanine NDI derivatives (**5**) with c-KIT1, along with the β-homoleucine (**4**) and α-boc-aminoalanine NDI derivatives (**5**) with h-TELO. Due to the planar NDI core being identical between enantiomers, the selective binding behaviour must be due to the point chirality in the amino acid side chains. The differences in interaction between the NDIs and the different G4 conformations, along with the observed enantioselective binding, imply that that the enantioselectivity is defined by the interaction between the chiral amino acid side chain and the sugar–phosphate backbone. The results indicate a preference for parallel G4 sequences with **5** and hybrid sequences with **4**, driven by the different β-amino acid chains and the point chirality within them.

The titration data and GNINA modelling support the enantioselective interaction between some NDIs and G4. Both (*S*)-enantiomers of **4** and **5** show a stronger binding affinity with the G4-DNA versus the (*R*)-enantiomers. The data also indicate that the α-boc-aminoalanine NDI derivatives (**5**) are within the 10 µM effective range for G4 ligands.

This work helps to solidify di-substituted NDIs as a potent scaffold for selective binding with G4 DNA and paves the way for future investigation into the effect of point chirality on the binding interaction with different G4 conformations.

## Figures and Tables

**Table 1:** The T_m_ and ΔT_m_ values for c-KIT1 and each of the c-KIT1-NDI assemblies, respectively, determined from the VT-CD studies at 2 μM c-KIT1 with five equivalents of NDI. The T_m_ values are averaged from three or more repeats and presented with standard error (SE).

Sequence/Assembly	Average T_m_ (°C)	ΔT_m_ (°C) ^1^
c-KIT1 only	61.2 ± 0.3	N/A
**1**	62.1 ± 0.2	0.9
**(*S*)-2**	64.4 ± 0.2	3.2
**(*R*)-3**	63.4 ± 0.2	2.2
**(*S*)-3**	62.7 ± 0.6	1.5
**(*R*)-4**	60.3 ± 0.9	−0.9
**(*S*)-4**	61.6 ± 0.6	0.4
**(*R*)-5**	62.9 ± 1.0	1.7
**(*S*)-5**	66.6 ± 1.3	5.4

^1^ The colours represent a heat map of the differential stabilisation of c-KIT1 by the different NDIs. Here, red is the least stabilising/most destabilising, whilst green is the most stabilising.

**Table 2:** The T_m_ and ΔT_m_ values for h-TELO and each of the h-TELO-NDI assemblies, respectively, determined from the VT-CD studies at 5 μM h-TELO with five equivalents of NDI. The T_m_ values are averaged from three or more repeats and presented with standard error (SE).

Sequence/Assembly	Average T_m_ (°C)	ΔT_m_ (°C) ^1^
h-TELO only	63.7 ± 0.8	N/A
**1**	64.6 ± 0.4	0.9
**(*S*)-2**	65.2 ± 0.7	1.5
**(*R*)-3**	64.1 ± 0.5	0.4
**(*S*)-3**	63.9 ± 0.2	0.2
**(*R*)-4**	64.3 ± 0.4	0.5
**(*S*)-4**	65.4 ± 1.7	1.7
**(*R*)-5**	64.2 ± 0.5	0.4
**(*S*)-5**	66.2 ± 2.0	2.4

^1^ The colours represent a heat map of the differential stabilisation of h-TELO by the different NDIs. Here, red is the least stabilising/most destabilising, whilst green is the most stabilising.

**Table 3:** The T_m_ and ΔT_m_ values for TBA and each of the TBA-NDI assemblies, respectively, determined from the VT-CD studies at 5 μM TBA with five equivalents of NDI. The Tm values are averaged from three or more repeats and presented with standard error (SE).

Sequence/Assembly	Average T_m_ (°C)	ΔT_m_ (°C) ^1^
TBA only	50.2 ± 0.4	N/A
**1**	51.0 ± 0.1	0.8
**(*S*)-2**	50.4 ± 0.1	0.2
**(*R*)-3**	50.5 ± 0.2	0.3
**(*S*)-3**	50.3 ± 0.1	0.1
**(*R*)-4**	50.6 ± 0.3	0.4
**(*S*)-4**	50.1 ± 0.1	−0.1
**(*R*)-5**	50.6 ± 0.2	0.4
**(*S*)-5**	50.3 ± 0.1	0.1

^1^ The colours represent a heat map of the differential stabilisation of TBA by the different NDIs. Here, red is the least stabilising/most destabilising, whilst green is the most stabilising.

**Table 4:** The T_m_ and ΔT_m_ values for dsDNA and each of the dsDNA–NDI assemblies, respectively, determined from the VT-CD studies at 5 μM dsDNA with five equivalents of NDI. The Tm values are averaged from three or more repeats and presented with standard error (SE).

Sequence/Assembly	Average T_m_ (°C)	ΔT_m_ (°C) ^1^
dsDNA only	61.9 ± 1.5	N/A
**1**	61.8 ± 0.5	−0.1
**(*S*)-2**	60.2 ± 0.8	−1.6
**(*R*)-3**	61.7 ± 1.2	−0.2
**(*S*)-3**	59.8 ± 0.1	−2.1
**(*R*)-4**	61.8 ± 0.4	−0.1
**(*S*)-4**	60.6 ± 0.4	−1.3
**(*R*)-5**	60.7 ± 1.0	−1.2
**(*S*)-5**	60.0 ± 1.1	−1.9

^1^ The colours represent a heat map of the differential stabilisation of dsDNA by the different NDIs. Here, red is the least stabilising/most destabilising, whilst green is the most stabilising.

**Table 5:** The dissociation coefficients (K_D_) values for **4** and **5** with h-TELO and c-KIT1 DNA, respectively. All experiments were performed in 100 mM K^+^ PBS. The K_D_ values were calculated from the change in absorbance at 238/383 nm with G4-NDI and 383 nm for dsDNA–NDI.

Ligand + DNA	K_D_ (μM)	R^2^
**(*R*)-5** + c-KIT1	8.0 ± 2.5	0.974
**(*S*)-5** + c-KIT1	2.6 ± 0.4	0.953
**(*R*)-4** + h-TELO	13.6 ± 3.4	0.992
**(*S*)-4** + h-TELO	11.0 ± 3.1	0.971
**(*R*)-5** + dsDNA	30.7 ± 6.9	0.997
**(*S*)-5** + dsDNA	65.2 ± 35.9	0.995
**(*R*)-4** + dsDNA	50.2 ± 15.4	0.992
**(*S*)-4** + dsDNA	23.6 ± 8.4	0.992

**Table 6:** A sequence comparison between the G4 sequences utilised experimentally and those used in modelling from the PDB. In some instances, the sequence from the PDB could be “trimmed” to be more in-line with the experimental sequence; these are labelled as amended.

Sequence Name	Sequence Composition (5′-3′)
c-KIT1 (used experimentally)	GGG AGG GCG CTG GGA GGA GGG
Amended PDB ID: 3QXR (c-KIT1)	GGG AGG GCG CUG GGA GGA GGG
h-TELO (used experimentally)	AGG GTT AGG GTT AGG GTT AGG GT
Amended PDB ID: 6CCW (h-TELO hybrid)	AGG GTT AGG GTT AGG GTT AGG GT
TBA (used experimentally)	GGT TGG TGT GGT TGG
PDB ID: 5MJX (TBA)	GGT TGG TGT GGT TGG

## Data Availability

All data underlying the findings of this work are available from the corresponding author upon reasonable request.

## Supplementary Materials

The following supporting information can be downloaded at https://www.mdpi.com/article/10.3390/molecules28217291/s1: Table S1: The change in absorbance between 20 °C and 80 °C for each NDI at 150 μM in PBS; Figure S1: UV-Vis absorption trace for **(*R*)-5** showing the three major peaks at 383 nm, 362 nm, and a broader shoulder at 333 nm. This general trace is true for all of the NDIs; Figure S2: ^1^H NMR of **1** in *d*^6^-DMSO; Figure S3: ^13^C NMR of **1** in *d*^6^-DMSO; Figure S4: ^1^H NMR of **(*S*)-2** in *d*^6^-DMSO; Figure S5: ^13^C NMR of **(*S*)-2** in *d*^6^-DMSO; Figure S6: ^1^H NMR of **(*R*)-3** in *d*^6^-DMSO; Figure S7: ^13^C NMR of **(*R*)-3** in *d*^6^-DMSO; Figure S8: ^1^H NMR of **(*S*)-3** in *d*^6^-DMSO; Figure S9: ^13^C NMR of **(*S*)-3** in *d*^6^-DMSO; Figure S10: ^1^H NMR of **(*R*)-4** in *d*^6^-DMSO; Figure S11: ^13^C NMR of **(*R*)-4** in *d*^6^-DMSO; Figure S12: ^1^H NMR of **(*S*)-4** in *d^6^*-DMSO; Figure S13: ^13^C NMR of **(*S*)-4** in *d^6^*-DMSO; Figure S14: ^1^H NMR of **(*R*)-5** in *d^6^*-DMSO; Figure S15: ^13^C NMR of **(*R*)-5** in *d^6^*-DMSO; Figure S16: ^1^H NMR of **(*S*)-5** in *d^6^*-DMSO; Figure S17: ^13^C NMR of **(*S*)-5** in *d^6^*-DMSO; Figure S18: Each of the c-KIT1-NDI melting plots (2 µM DNA with 5 equivalents of NDI): (a) c-KIT1 only; (b) c-KIT1 + **1**; (c) c-KIT1 + **(*S*)-2**; (d) c-KIT1 + **(*R*)-3**; (e) c-KIT1 + **(*S*)-3**; (f) c-KIT1 + **(*R*)-4**; (g) c-KIT1 + **(*S*)-4**; (h) c-KIT1 + **(*R*)-5**; and (i) c-KIT1 + **(*S*)-5**. These plots are averaged of at least three experimental runs.; Figure S19: Each of the h-TELO-NDI melting plots (5 µM DNA with 5 equivalents of NDI): (a) h-TELO only; (b) h-TELO + **1**; (c) h-TELO + **(*S*)-2**; (d) h-TELO + **(*R*)-3**; (e) h-TELO + **(*S*)-3**; (f) h-TELO + **(*R*)-4**; (g) h-TELO + **(*S*)-4**; (h) h-TELO + **(*R*)-5**; and (i) h-TELO + **(*S*)-5**. These plots are averaged of at least three experimental runs.; Figure S20: Each of the TBA-NDI melting plots (2 µM DNA with 5 equivalents of NDI): (a) TBA only; (b) TBA + **1**; (c) TBA + **(*S*)-2**; (d) TBA + **(*R*)-3**; (e) TBA + **(*S*)-3**; (f) TBA + **(*R*)-4**; (g) TBA + **(*S*)-4**; (h) TBA + **(*R*)-5**; and (i) TBA + **(*S*)-5**. These plots are averaged of at least three experimental runs.; Figure S21: Each of the dsDNA–NDI melting plots (2 µM DNA with 5 equivalents of NDI): (a) dsDNA only; (b) dsDNA + **1**; (c) dsDNA + **(*S*)-2**; (d) dsDNA + **(*R*)-3**; (e) dsDNA + **(*S*)-3**; (f) dsDNA + **(*R*)-4**; (g) dsDNA + **(*S*)-4**; (h) dsDNA + **(*R*)-5**; and (i) dsDNA + **(*S*)-5**. These plots are averaged of at least three experimental runs.; Figure S22: Plot of the average T_m_ of c-KIT1 with each of the NDIs from two or more repeats, showing the standard error; Figure S23: Plot of the average T_m_ of h-TELO with each of the NDIs from two or more repeats, showing the standard error; Figure S24: Plot of the average T_m_ of TBA with each of the NDIs from two or more repeats, showing the standard error; Figure S25: Plot of the average T_m_ of dsDNA with each of the NDIs from two or more repeats, showing the standard error; Figure S26: Dissociation fit from a titration of 2 µM c-KIT1 with **(*R*)-5** at 238 nm; Figure S27: Dissociation fit from a titration of 2 µM c-KIT1 with **(*S*)-5** at 238 nm; Figure S28: Dissociation fit from a titration of 5 µM h-TELO with **(*R*)-4** at 383 nm; Figure S29: Dissociation fit from a titration of 5 µM h-TELO with **(*S*)-4** at 383 nm; Figure S30: Dissociation fit from a titration of 5 µM dsDNA with **(*R*)-5** at 383 nm; Figure S31: Dissociation fit from a titration of 5 µM dsDNA with **(*S*)-5** at 383 nm.; Figure S32: Dissociation fit from a titration of 5 µM dsDNA with **(*R*)-4** at 383 nm; Figure S33: Dissociation fit from a titration of 5 µM dsDNA with **(*S*)-4** at 383 nm; Figure S34: High-resolution mass spectrometry (HRMS) data for **1** with the (M + H)^+^ peak found at 623.1666; Figure S35: HRMS data for **(*S*)-2** with the (M + H)^+^ peak found at 443.0731; Figure S36: HRMS data for **(*R*)-3** with the (M + H)^+^ peak found at 439.1139; Figure S37: HRMS data for **(*S*)-3** with the (M + H)^+^ peak found at 439.1144; Figure S38: HRMS data for **(*R*)-4** with the (M + H)^+^ peak found at 523.2081; Figure S39: HRMS data for **(*S*)-4** with the (M + H)^+^ peak found at 523.2077; Figure S40: HRMS data for **(*R*)-5** with the (M + Na)^+^ peak found at 663.1911; Figure S41: HRMS data for **(*S*)-5** with the (M + Na)^+^ peak found at 663.1912; Table S2: Normal-phase HPLC gradient method for all NDIs; Figure S42: Normal-phase HPLC analysis at 383 nm of **1**; Table S3: The elution areas and times of the normal-phase HPLC analysis at 383 nm of **1**; Figure S43: Normal-phase HPLC analysis of **(*S*)-2**; Table S4: The elution areas and times of the normal-phase HPLC analysis at 383 nm of **(*S*)-2**; Figure S44: Normal-phase HPLC analysis of **(*R*)-3**; Table S5: The elution areas and times of the normal-phase HPLC analysis at 383 nm of **(*R*)-3**; Figure S45: Normal-phase HPLC analysis of **(*S*)-3**; Table S6: The elution areas and times of the normal-phase HPLC analysis at 383 nm of **(*S*)-3**; Figure S46: Normal-phase HPLC analysis of **(*R*)-4**; Table S7: The elution areas and times of the normal-phase HPLC analysis at 383 nm of **(*R*)-4**; Figure S47: Normal-phase HPLC analysis of **(*S*)-4**; Table S8: The elution areas and times of the normal-phase HPLC analysis at 383 nm of **(*S*)-4**; Figure S48: Normal-phase HPLC analysis of **(*R*)-5**; Table S9: The elution areas and times of the normal-phase HPLC analysis at 383 nm of **(*R*)-5**; Figure S49: Normal-phase HPLC analysis of **(*S*)-5**; Table S10: The elution areas and times of the normal-phase HPLC analysis at 383 nm of **(*S*)-5**.

## Sample Availability

Reasonable amounts of samples of the NDI derivatives are available from the authors.
