# Supplementary material for: The Influence of Chirality on the β-Amino-Acid Naphthalenediimides/G-Quadruplex DNA Interaction
Source: Molecules. 2023 Oct 27;28(21):7291. doi: 10.3390/molecules28217291 (PMC10647805; doi:10.3390/molecules28217291)
Supplement: Supplementary file 1 [file molecules-28-07291-s001.zip › molecules-2622217-supplementary.pdf]

**Supplementary Materials for**

# The Influence of Chirality on the β-Amino-Acid Naphthalenediimides / G-Quadruplex DNA Interaction

Samuel R. Clowes [1], Yusuf Ali [1], Olivia Astley [1], Dora M. Răsădean [1], and G. Dan Pantoş[1*]

[1] Department of Chemistry, University of Bath, Claverton Down, Bath, BA2 7AY, UK

*Correspondence: g.d.pantos@bath.ac.uk

**Contents**

1. NDI AGGREGATION STUDIES .......... 2

2. $^{1}$H NMR AND $^{13}$C NMR OF COMPOUNDS 1, (*S*)-2, (*R*)-3, (*S*)-3, (*R*)-4, (*S*)-4, (*R*)-5, AND (*S*)-5. .......... 4

3. G4 DNA-NDI VARIABLE-TEMPERATURE CIRCULAR DICHROISM (VT-CD) BOLTZMANN PLOTS .......... 12

3.1 C-KIT1 .......... 12
3.2 H-TELO .......... 13
3.3 TBA .......... 14
3.4 DSDNA .......... 15

4. PLOTS OF THE AVERAGE $T_M$ WITH STANDARD ERRORS .......... 16

5. DNA-NDI TITRATION FITS .......... 18

6. MASS SPECTROMETRY DATA .......... 22

7. HPLC DATA .......... 25

## 1. NDI Aggregation Studies

All the NDIs were designed to be soluble in aqueous solution however, due to the hydrophobic core, NDIs are prone to self-aggregation in PBS solution. Therefore, the NDIs were made up at various concentrations and tested using variable temperature (VT) UV-vis studies. The aggregation of the NDIs could be studied by monitoring absorbance as a function of temperature.

The NDI aggregation experiments were setup at 300, 150 and 75 μM in PBS. The variable temperature studies were setup from 20 – 75 °C picking the characteristic NDI absorbance wavelengths of 383, 362, and 333 nm (**Figure S1**). The change in absorbance as a function of temperature was plotted for each of them to determine whether self-aggregation was present. The change in absorbance ($\Delta_{abs}$) at 150 μM is highlighted in **table S1**.

**Table S1**: The change in absorbance between 20 °C and 80 °C for each NDI at 150 μM in PBS.

| NDI | $\Delta_{abs}$ (AU) |
|---|---|
| **1** | 0.10 |
| **(*S*)-2** | 0.08 |
| **(*R*)-3** | 0.15 |
| **(*S*)-3** | 0.11 |
| **(*R*)-4** | 0.05 |
| **(*S*)-4** | 0.05 |
| **(*R*)-5** | 0.12 |
| **(*S*)-5** | 0.10 |

From the absorption data for NDI at 150 μM in PBS, **(*S*)-2**, **(*R*)-3**, **(*S*)-3**, **(*R*)-4,** and **(*S*)-4** show a linear decrease as a function of temperature, indicating that there is no deaggregation process occurring. For **1**, **(*R*)-5**, and **(*S*)-5**, there was a linear decrease that ended with a plateau at higher temperatures, implying that a deaggregation event occurred as a result of the increased temperature. Consequently, to overcome the aggregation potential of the NDIs, all solutions were sonicated for 30 mins before preparation of DNA-NDI solutions for VT-CD.

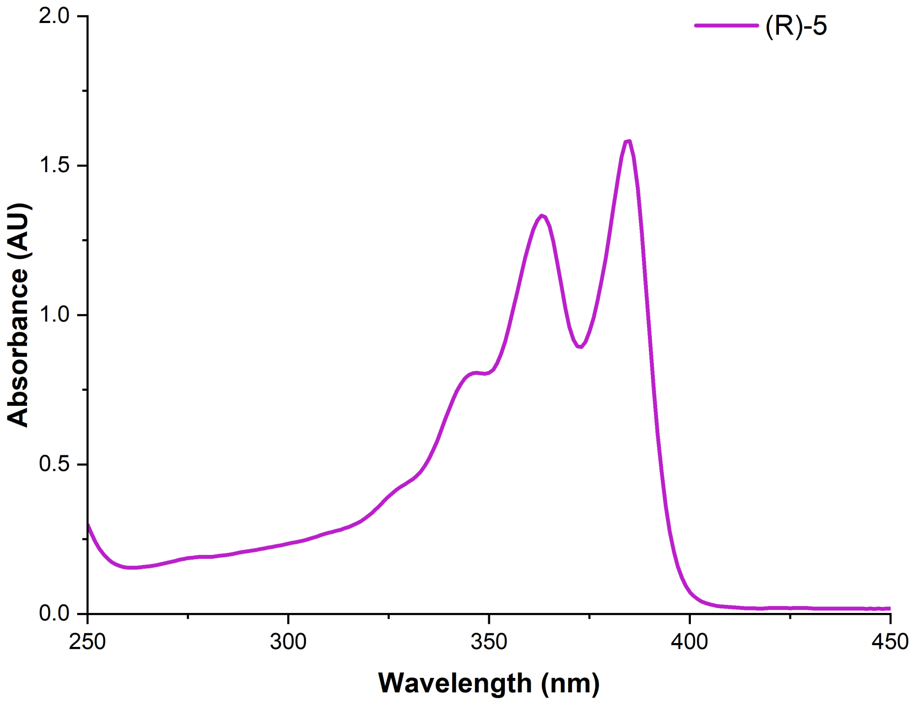


**Figure S1**: UV-Vis absorption trace for **(*R*)-5** showing the three major peaks at 383 nm, 362 nm, and a broader shoulder at 333 nm. This general trace is true for all of the NDIs.

## 2. $^1$H NMR and $^{13}$C NMR of compounds 1, (*S*)-2, (*R*)-3, (*S*)-3, (*R*)-4, (*S*)-4, (*R*)-5, and (*S*)-5.

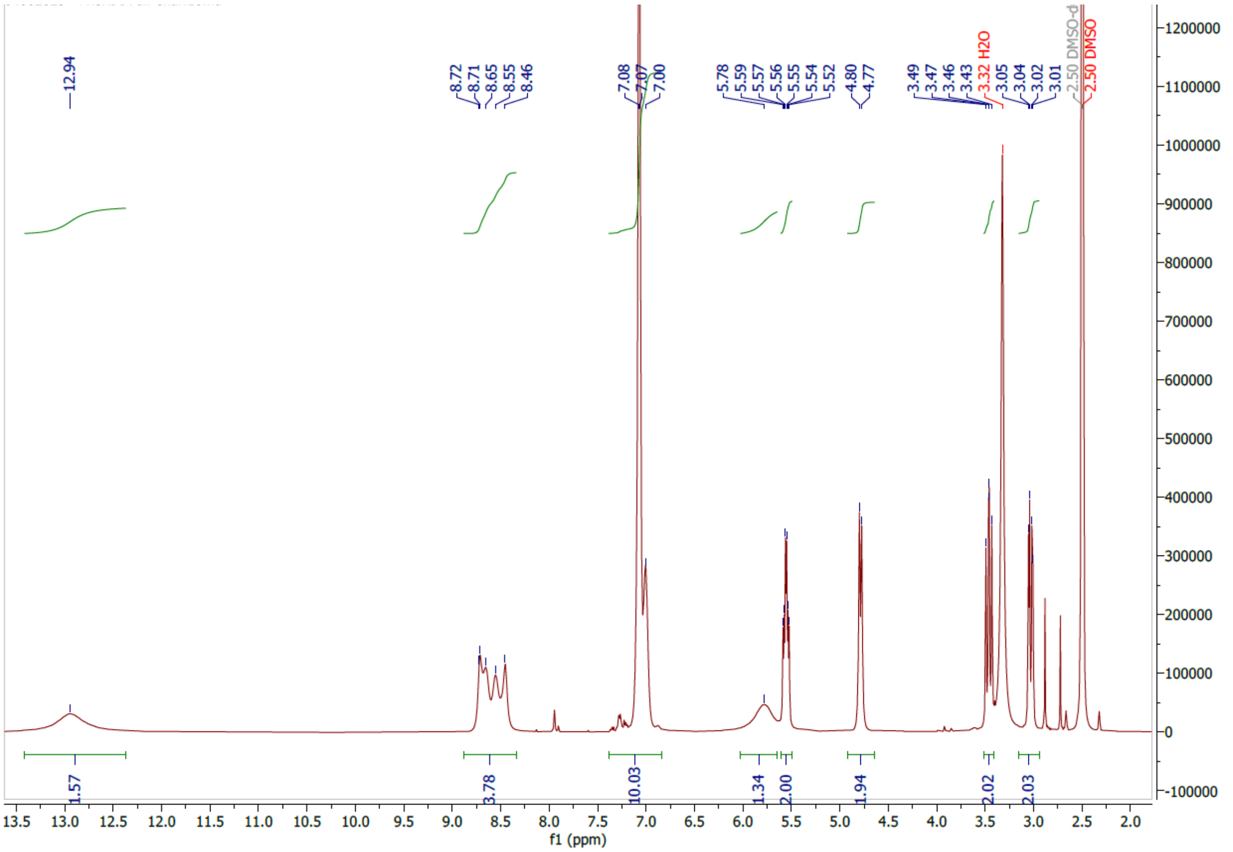


**Figure S2**: $^1$H NMR of **1** in $d^6$-DMSO. The observed lack of symmetry is due to the slow rotation of the phenyl group with respect to the NDI core. This is a result of the intra-molecular π-π aromatic interactions between these two functionalities.

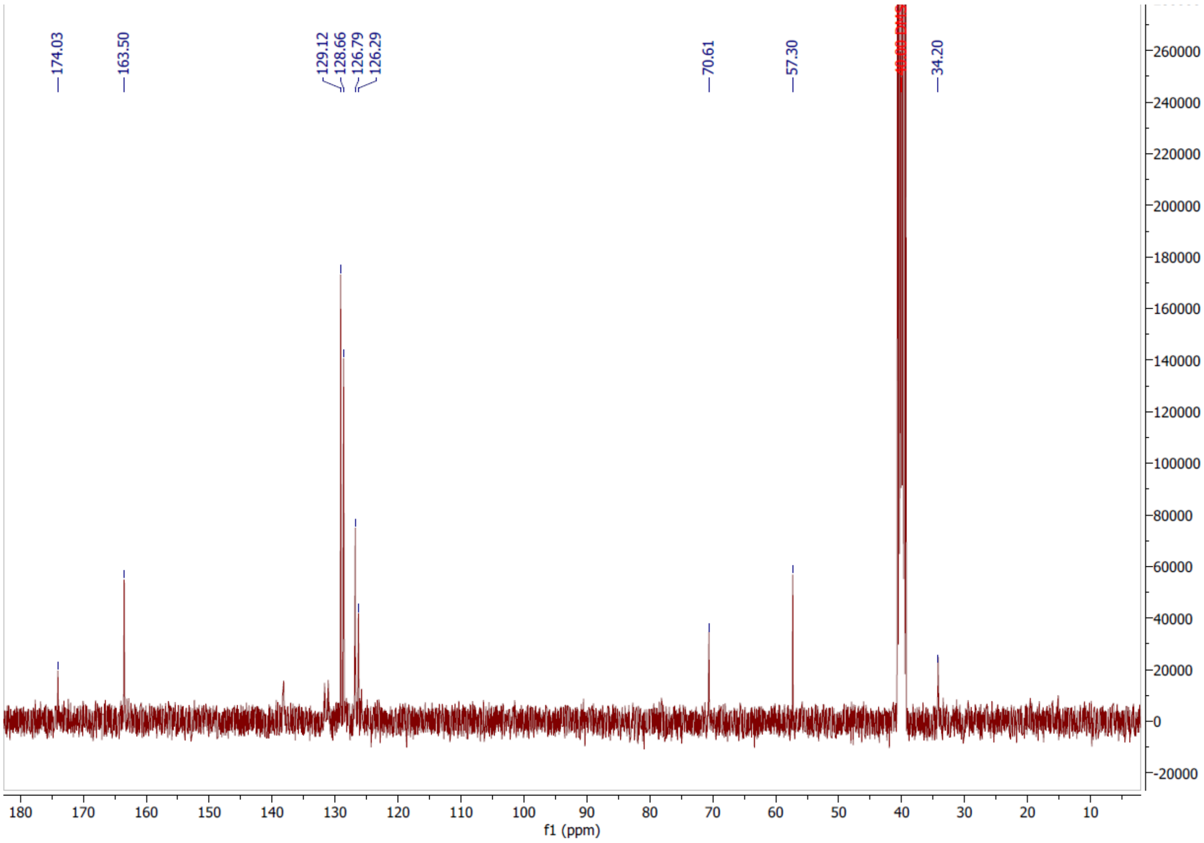


**Figure S3**: $^{13}$C NMR of **1** in $d^6$-DMSO.

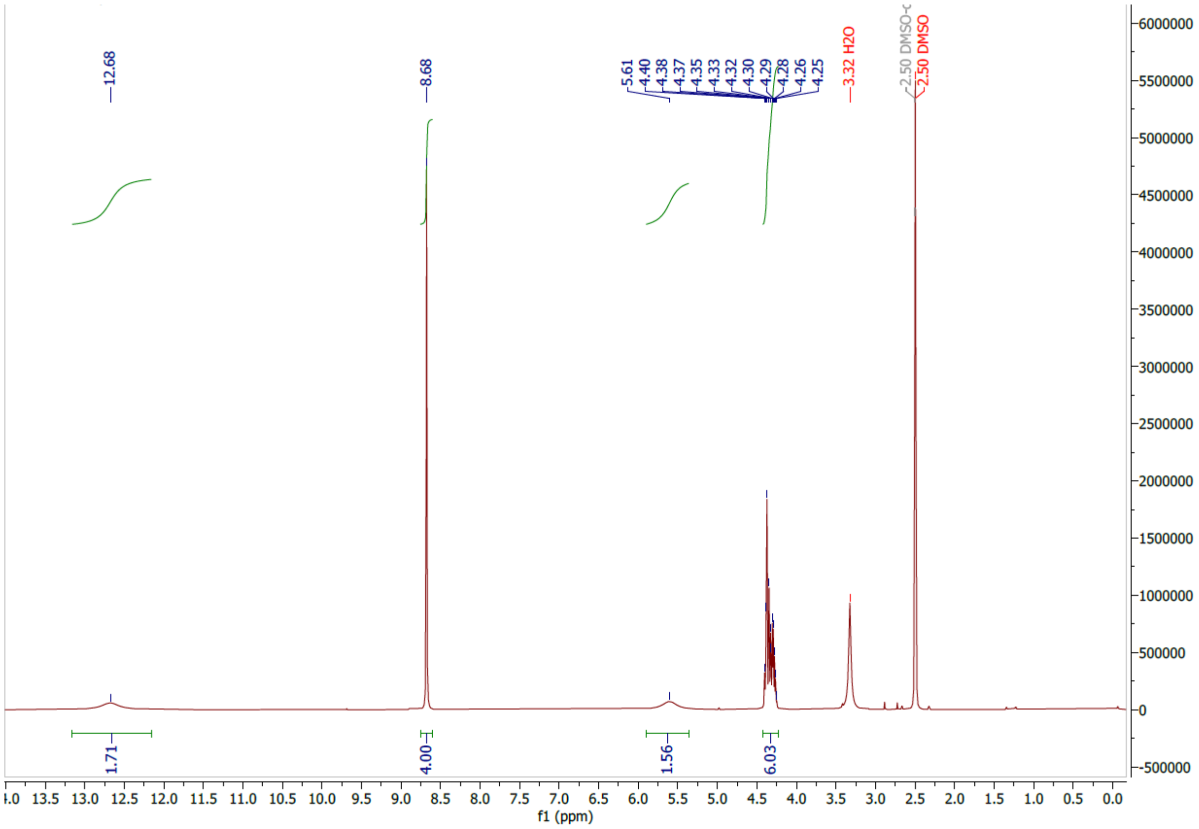


**Figure S4**: $^{1}$H NMR of **(*S*)-2** in *d*[6]-DMSO.

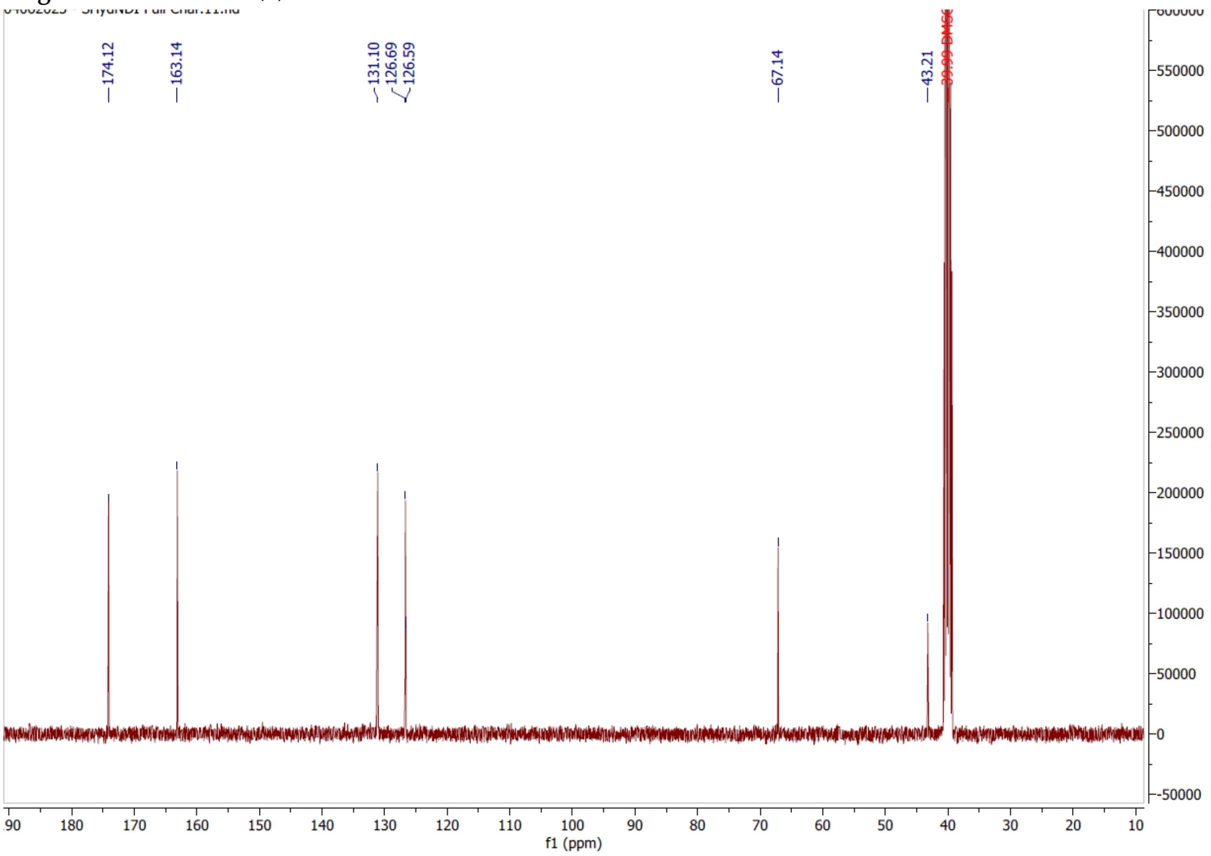


**Figure S5**: $^{13}$C NMR of **(*S*)-2** in *d*[6]-DMSO.

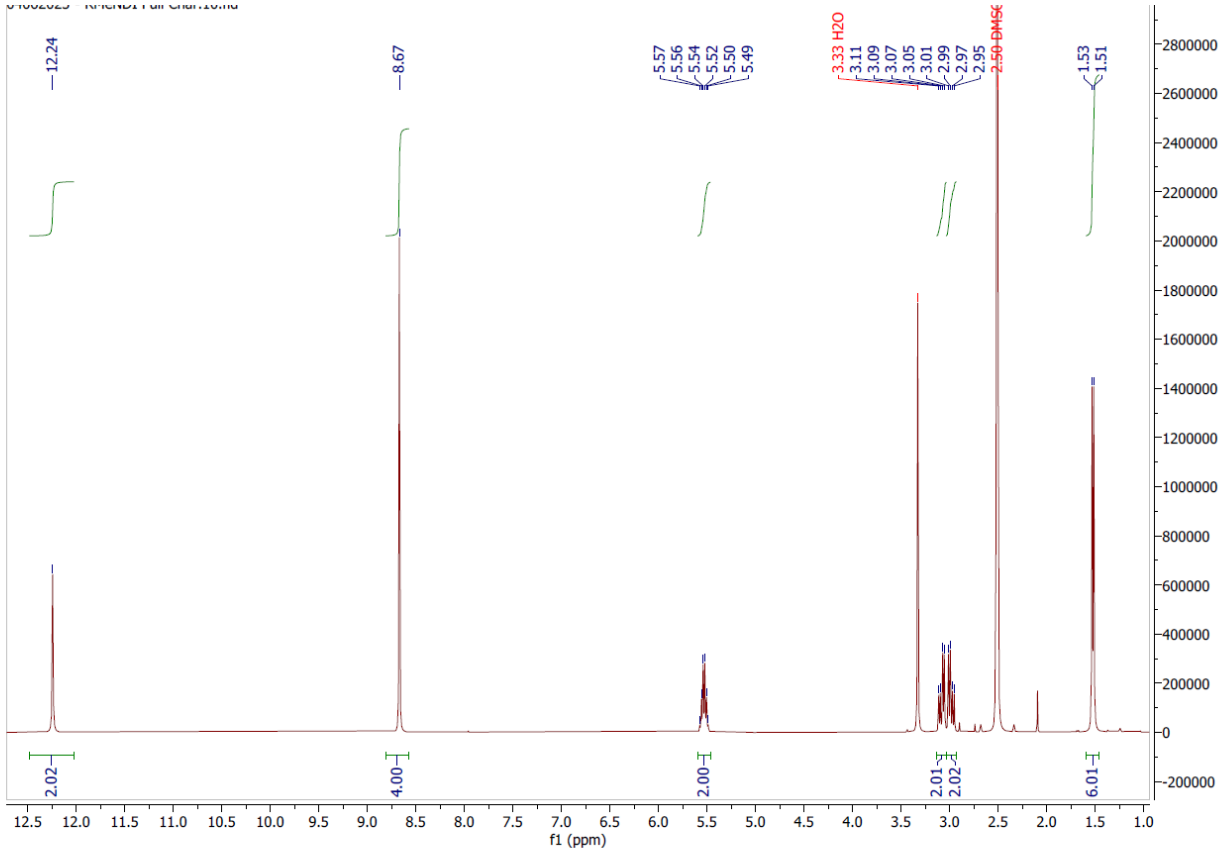


**Figure S6**: $^{1}$H NMR of **(*R*)-3** in *d*[6]-DMSO.

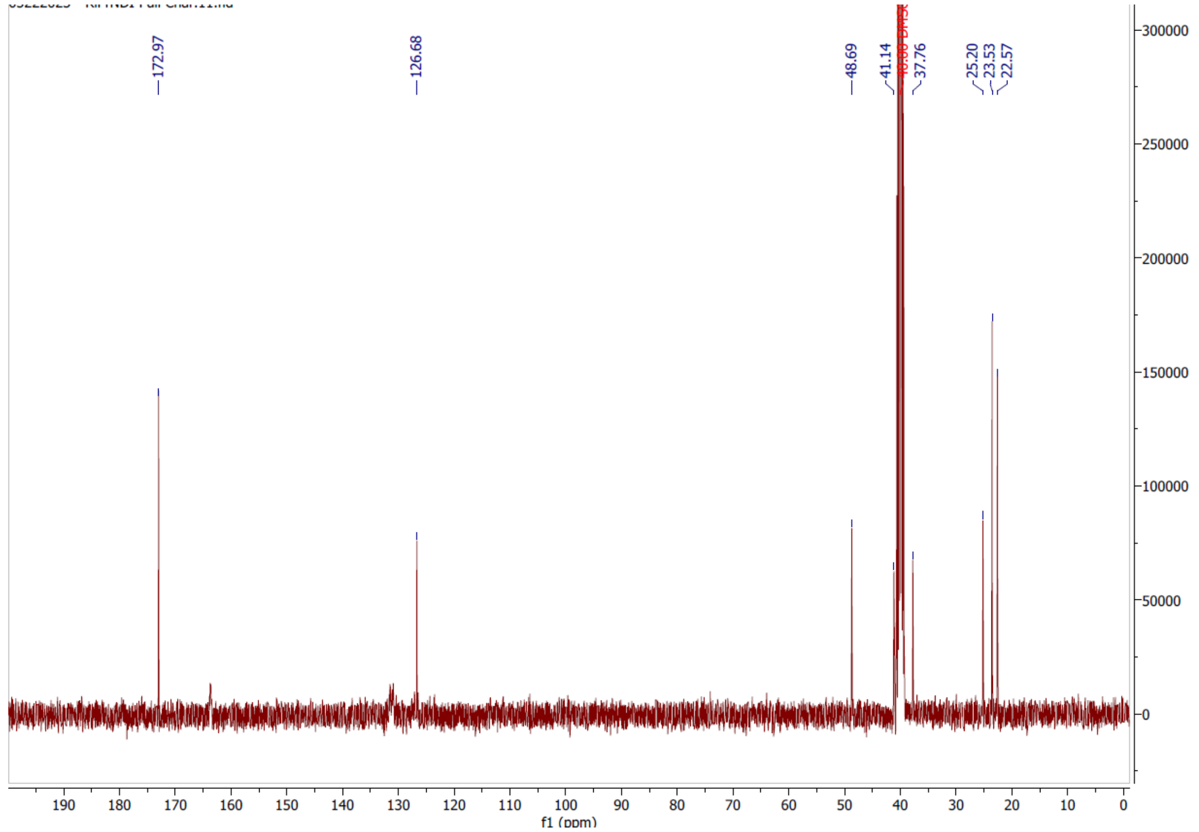


**Figure S7**: $^{13}$C NMR of **(*R*)-3** in *d*[6]-DMSO.

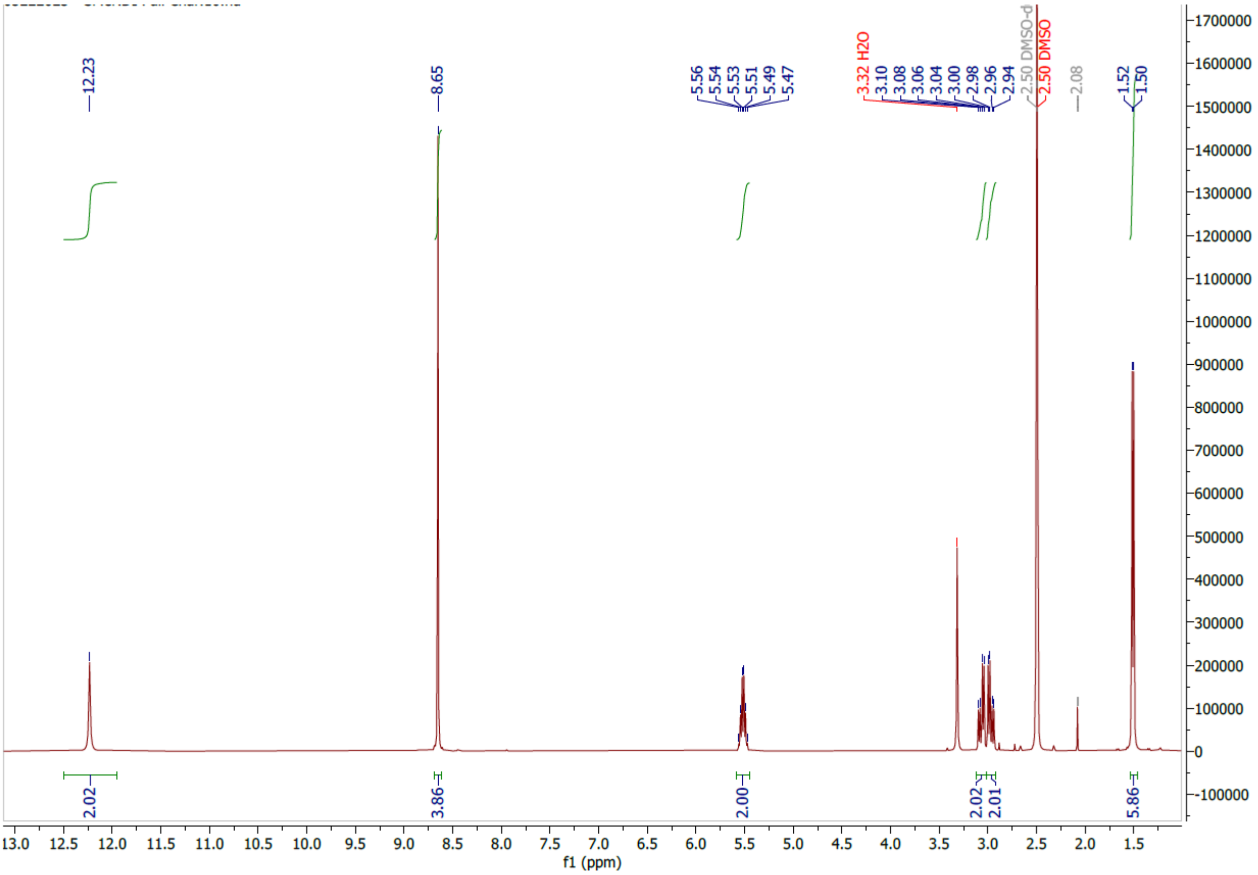


**Figure S8**: $^{1}H$ NMR of **(*S*)-3** in *d*$^{6}$-DMSO.

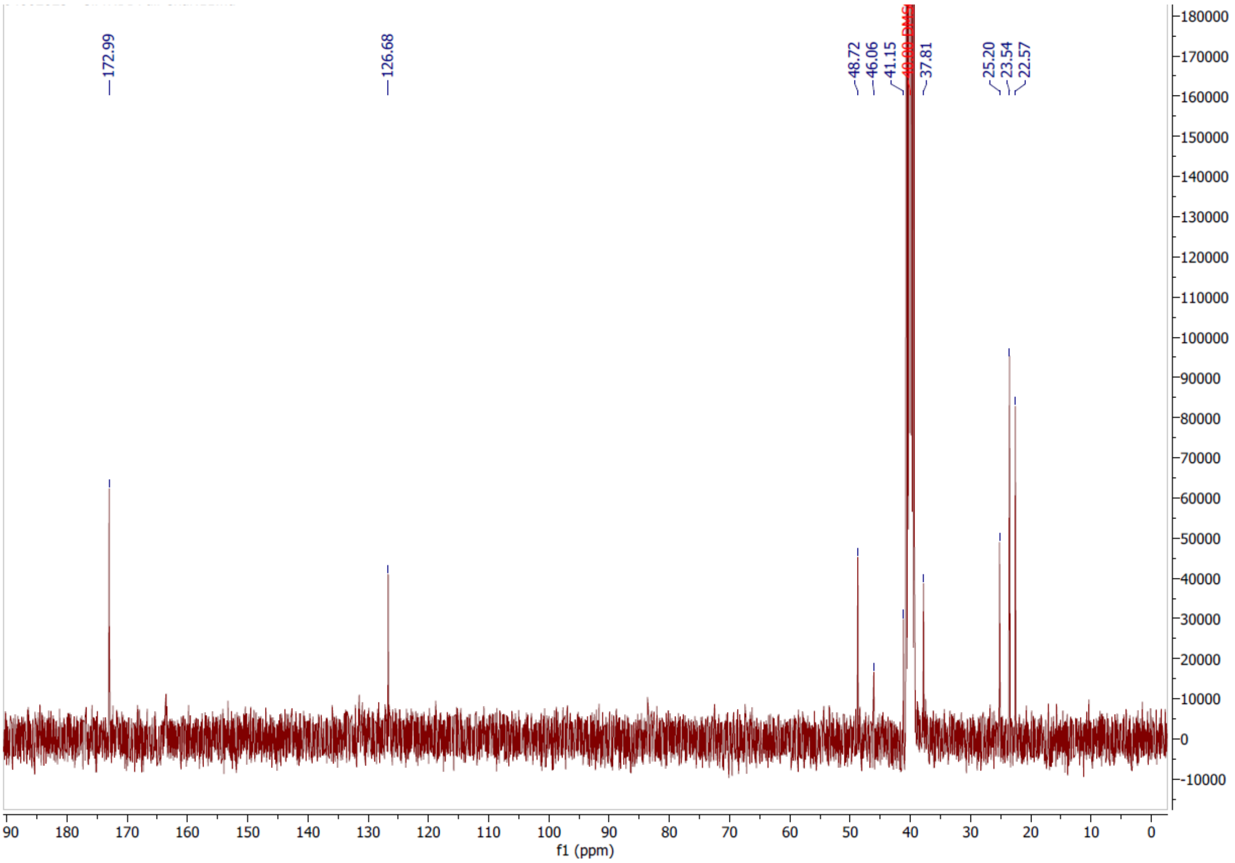


**Figure S9**: $^{13}C$ NMR of **(*S*)-3** in *d*$^{6}$-DMSO.

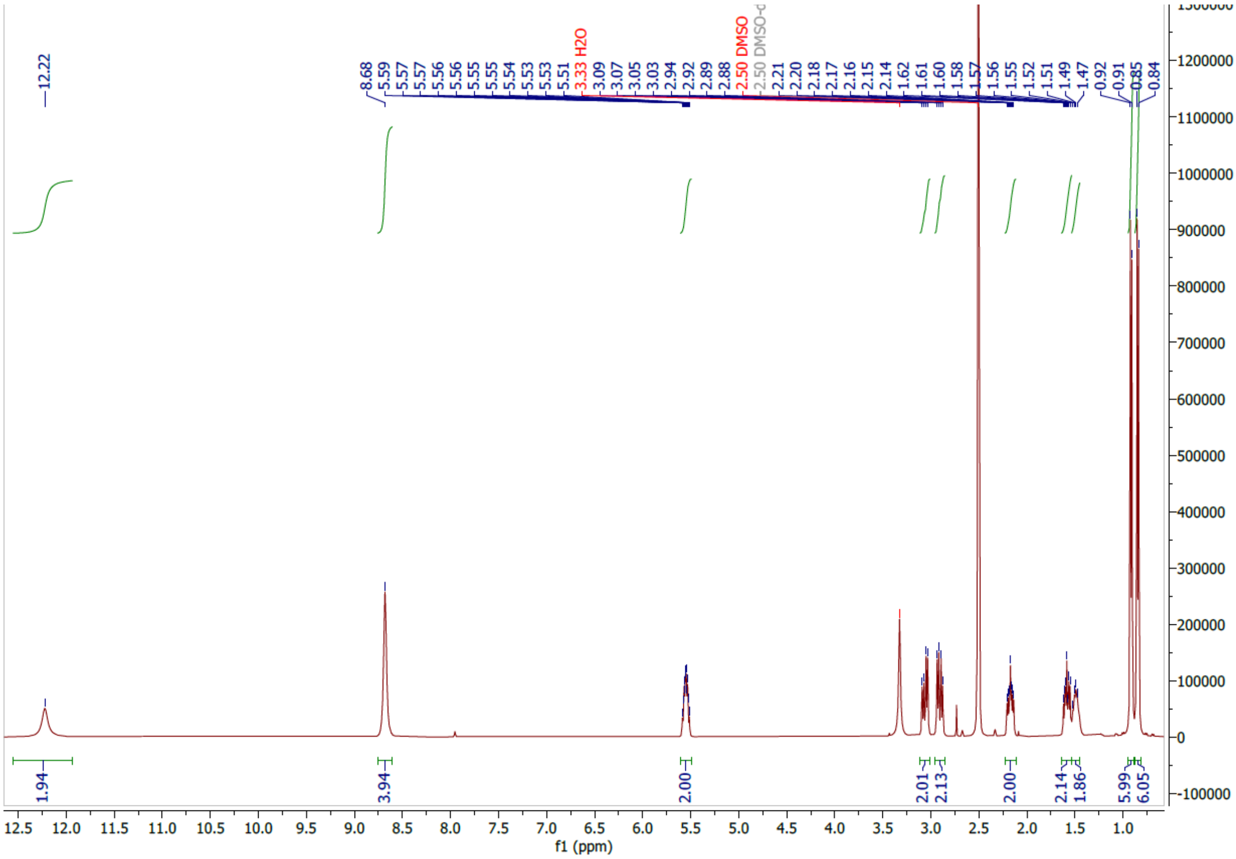


**Figure S10**: $^{1}$H NMR of **(*R*)-4** in $d^{6}$-DMSO.

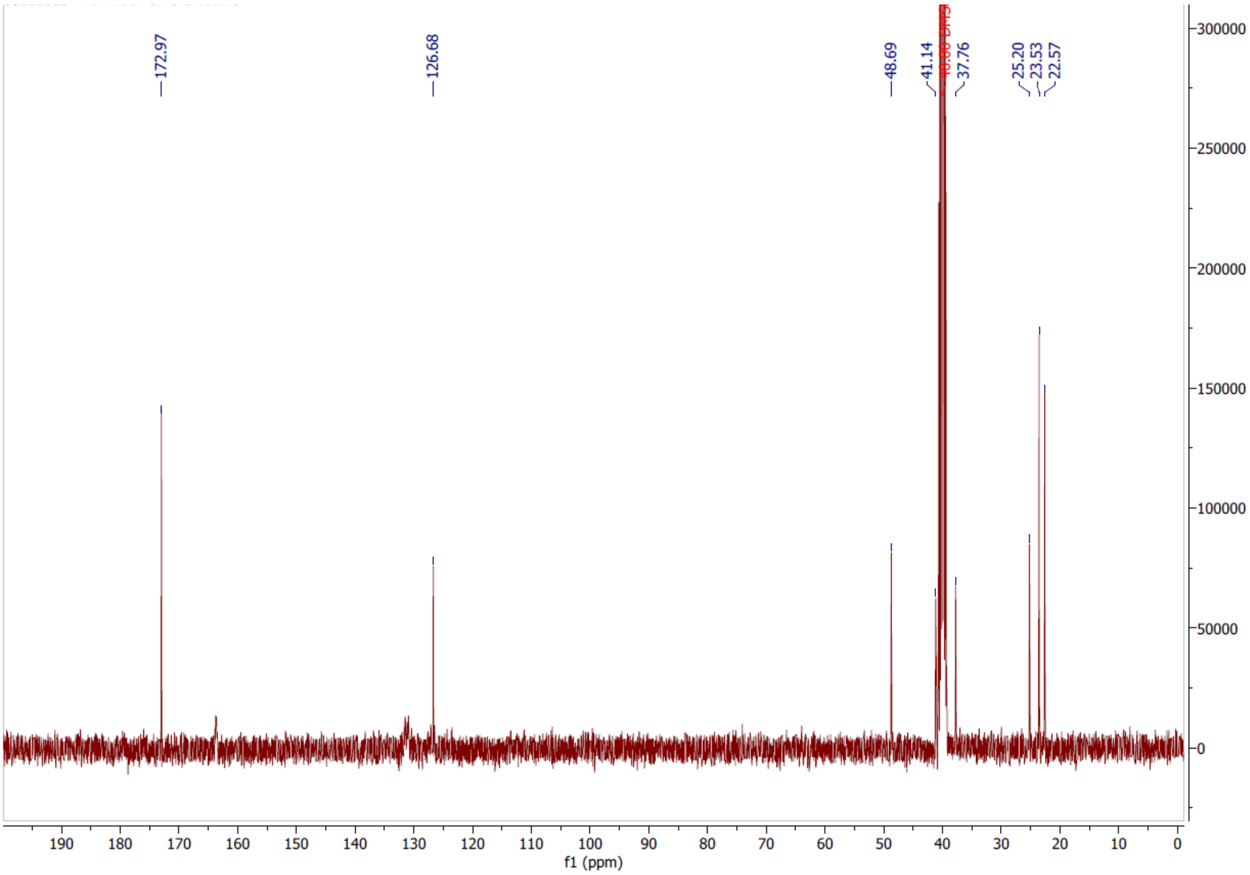


**Figure S11**: $^{13}$C NMR of **(*R*)-4** in $d^{6}$-DMSO.

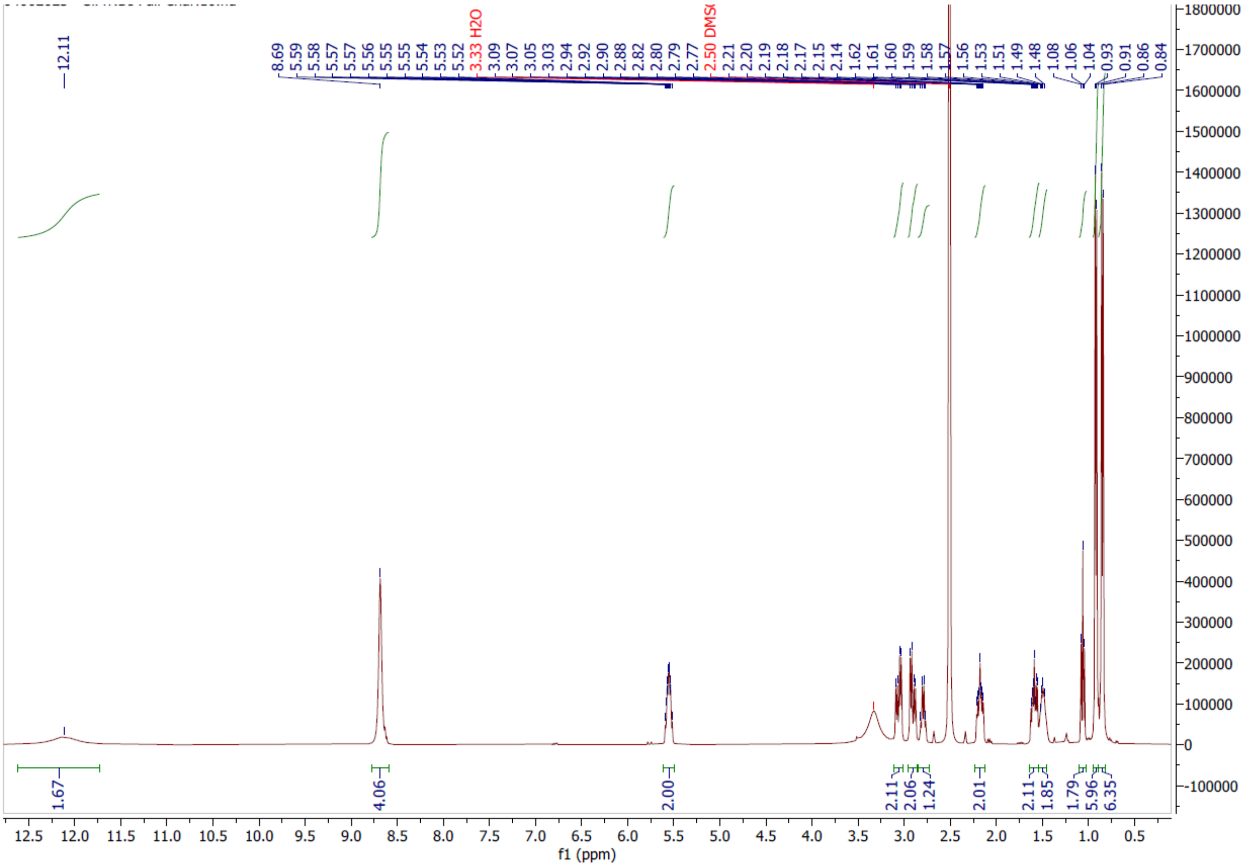


**Figure S12**: $^{1}H$ NMR of **(*S*)-4** in $d^6$-DMSO.

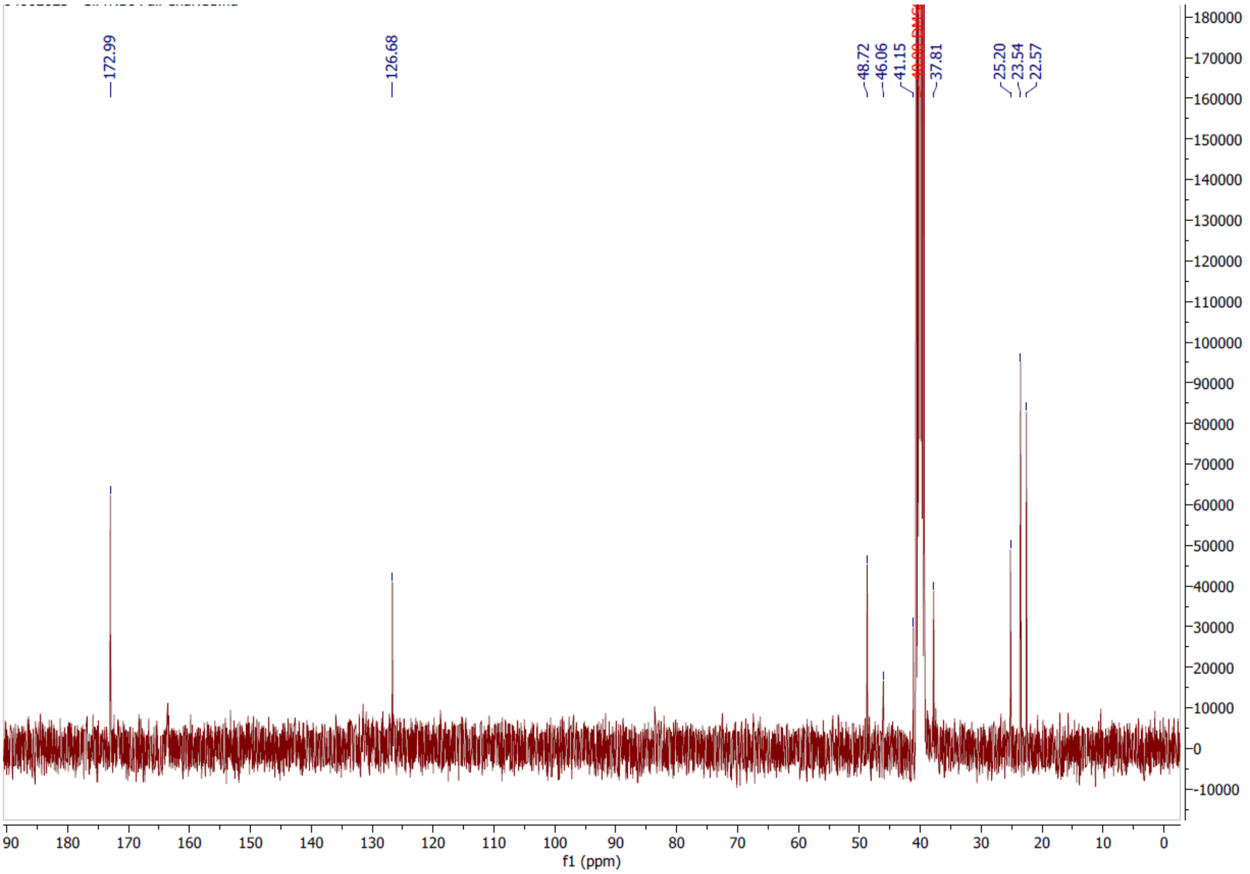


**Figure S13**: $^{13}C$ NMR of **(*S*)-4** in $d^6$-DMSO.

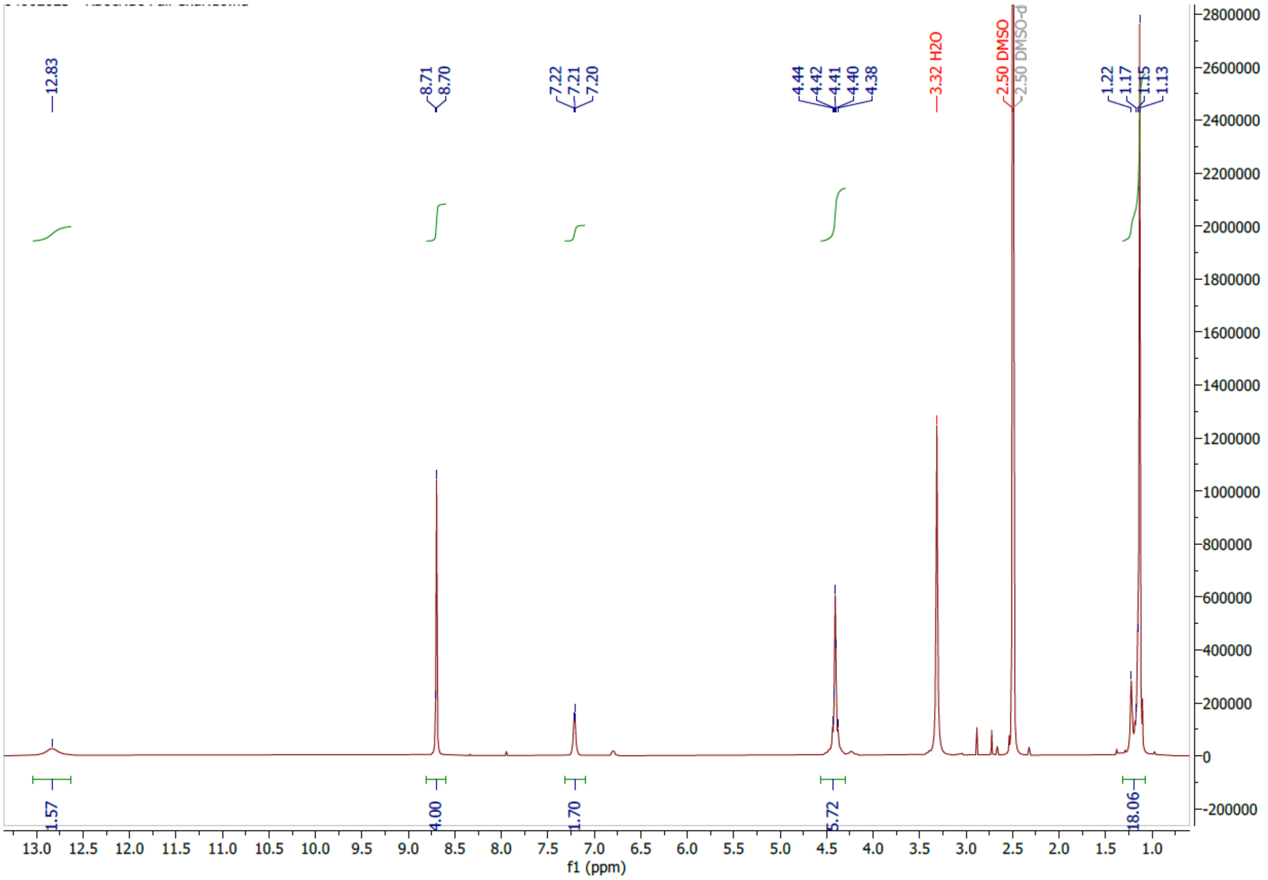


**Figure S14**: $^{1}$H NMR of **(*R*)-5** in *d*[6]-DMSO.

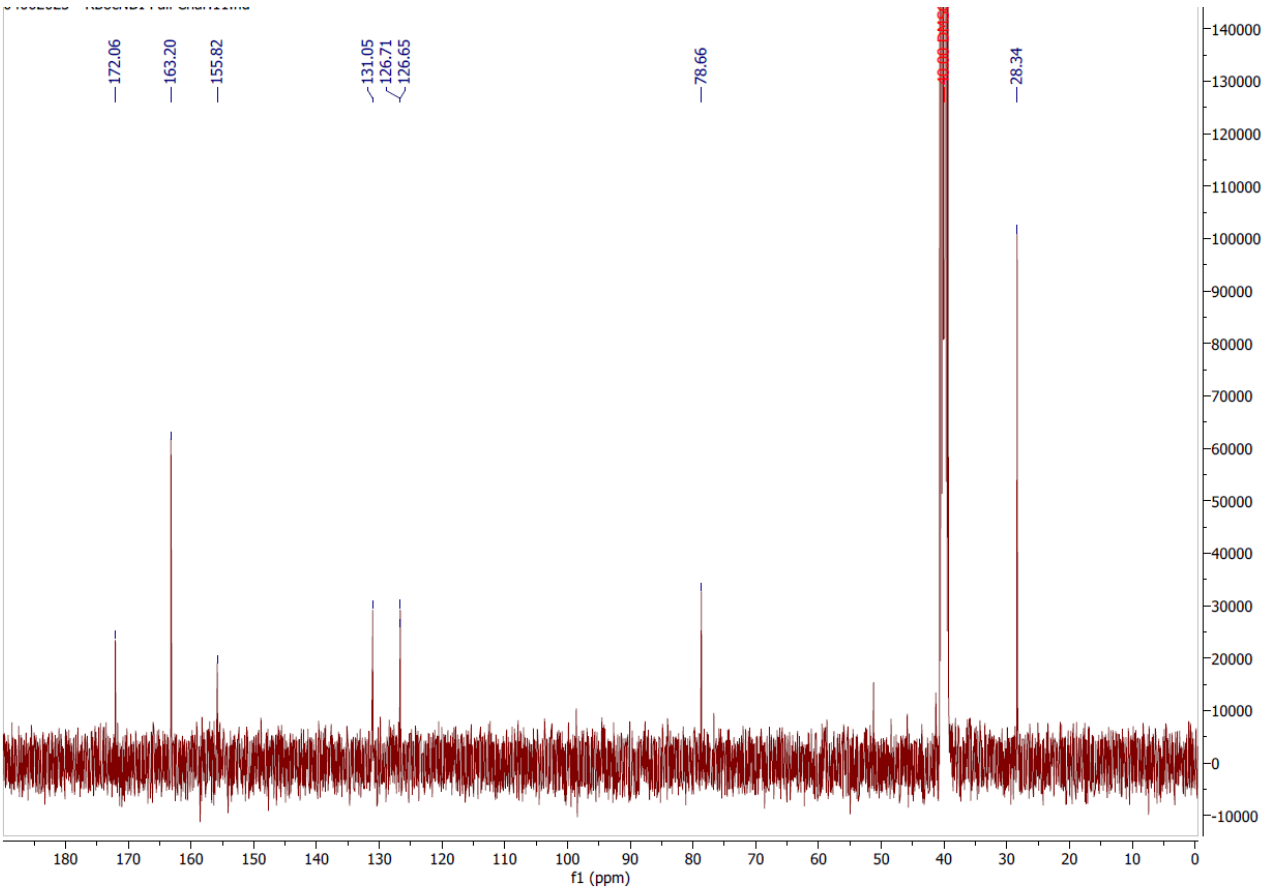


**Figure S15**: $^{13}$C NMR of **(*R*)-5** in *d*[6]-DMSO.

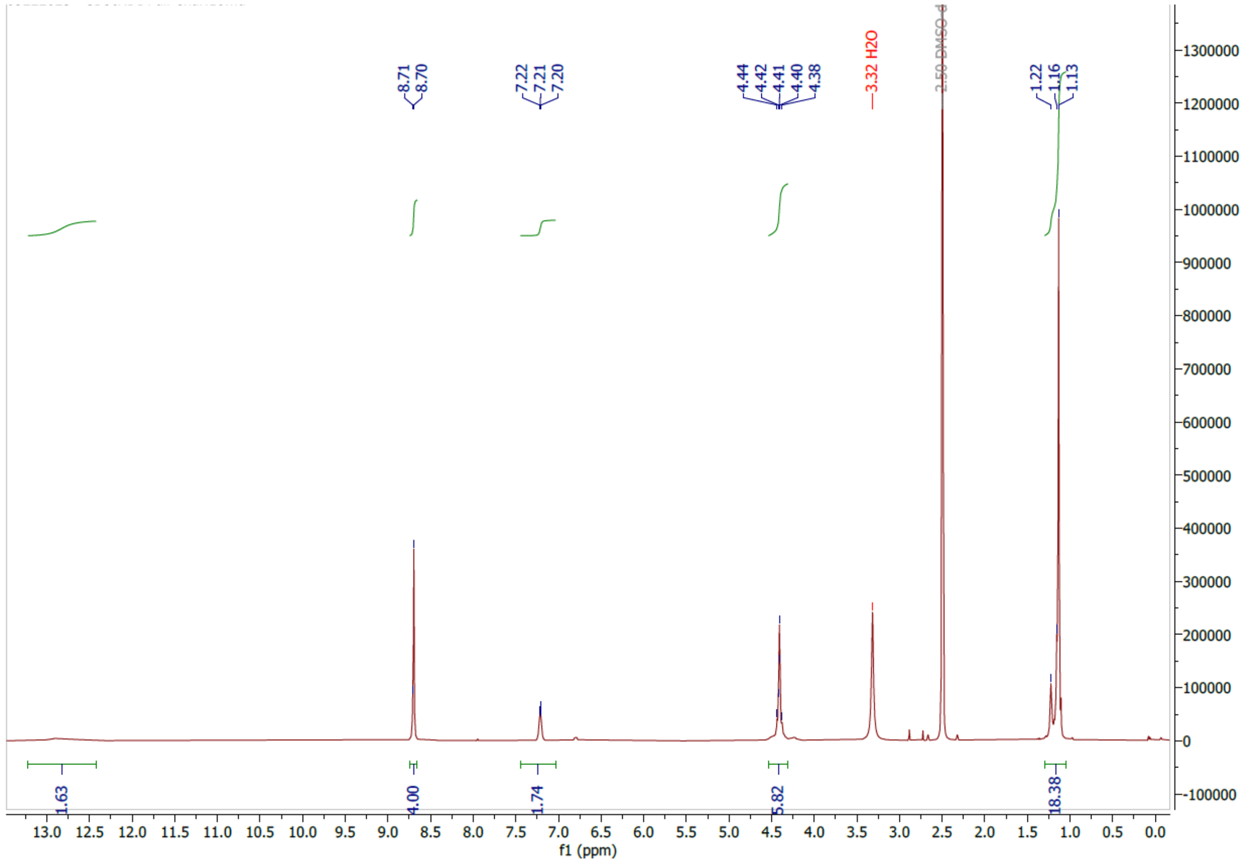


**Figure S16**: $^{1}H$ NMR of **(*S*)-5** in $d^{6}$-DMSO.

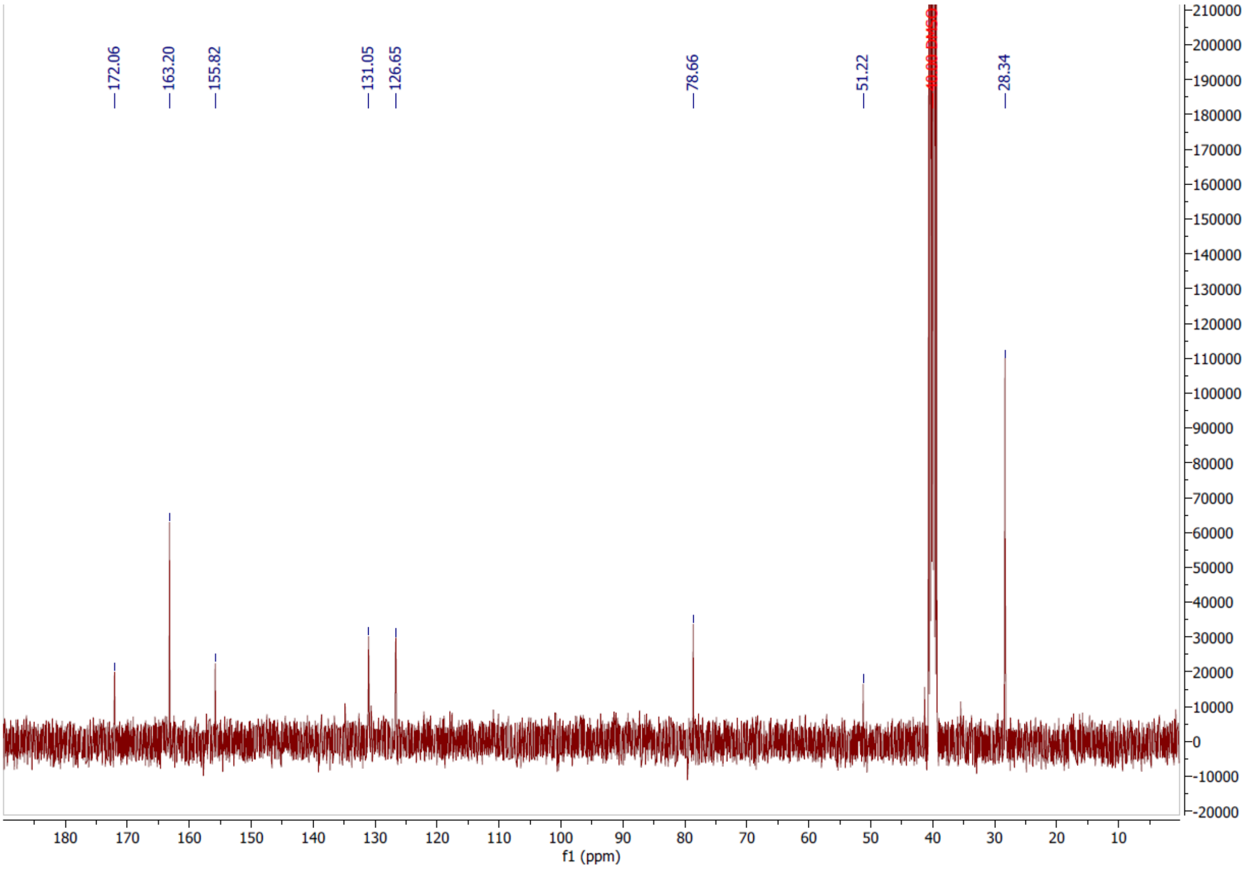


**Figure S17**: $^{13}C$ NMR of **(*S*)-5** in $d^{6}$-DMSO.

## 3. G4 DNA-NDI Variable-Temperature Circular Dichroism (VT-CD) Boltzmann Plots

### 3.1 c-KIT1

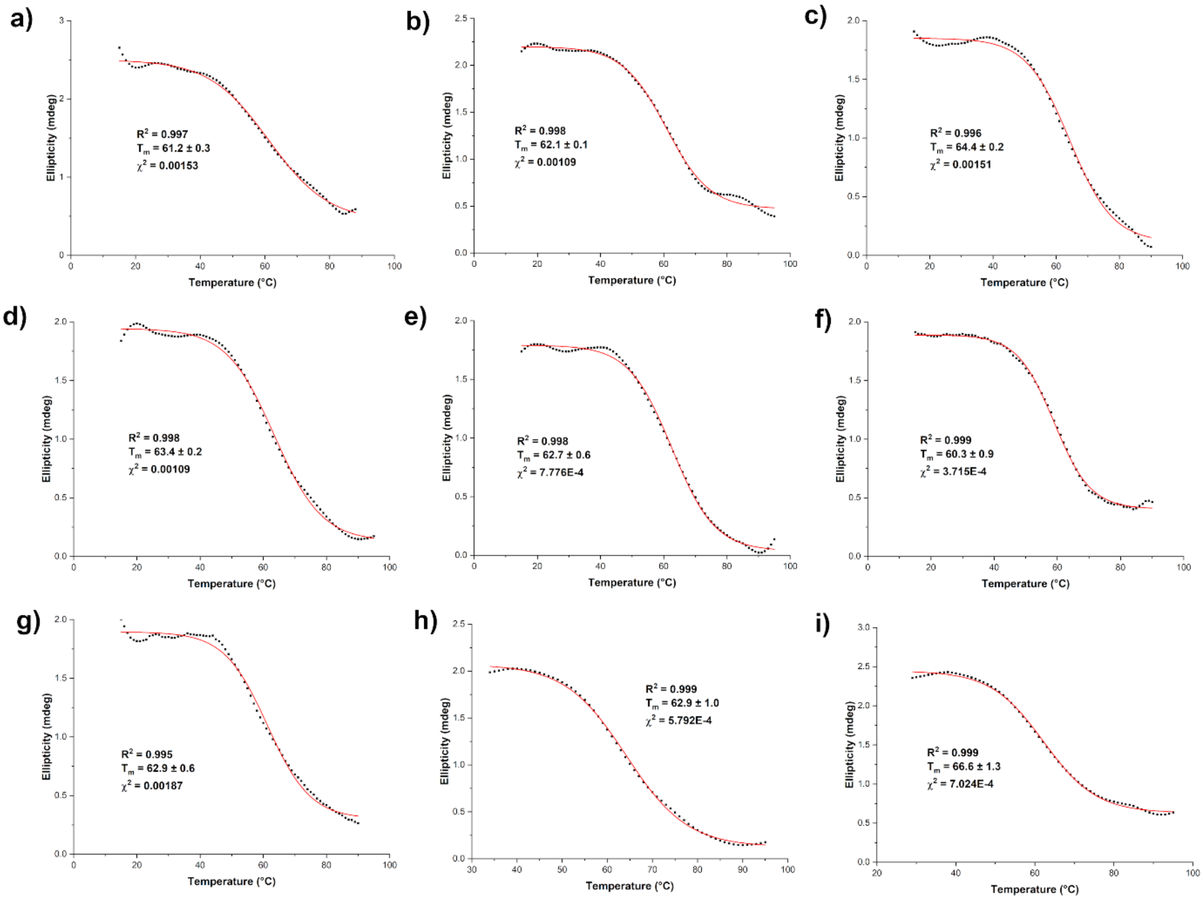


**Figure S18**: Each of the c-KIT1-NDI melting plots (2 μM DNA with 5 equivalents of NDI): **a**) c-KIT1 only; **b**) c-KIT1 + **1**; **c**) c-KIT1 + **(*S*)-2**; **d**) c-KIT1 + **(*R*)-3**; **e**) c-KIT1 + **(*S*)-3**; **f**) c-KIT1 + **(*R*)-4**; **g**) c-KIT1 + **(*S*)-4**; **h**) c-KIT1 + **(*R*)-5**; and **i**) c-KIT1 + **(*S*)-5**. These plots are averaged of at least three separate experimental runs.

### 3.2 h-TELO

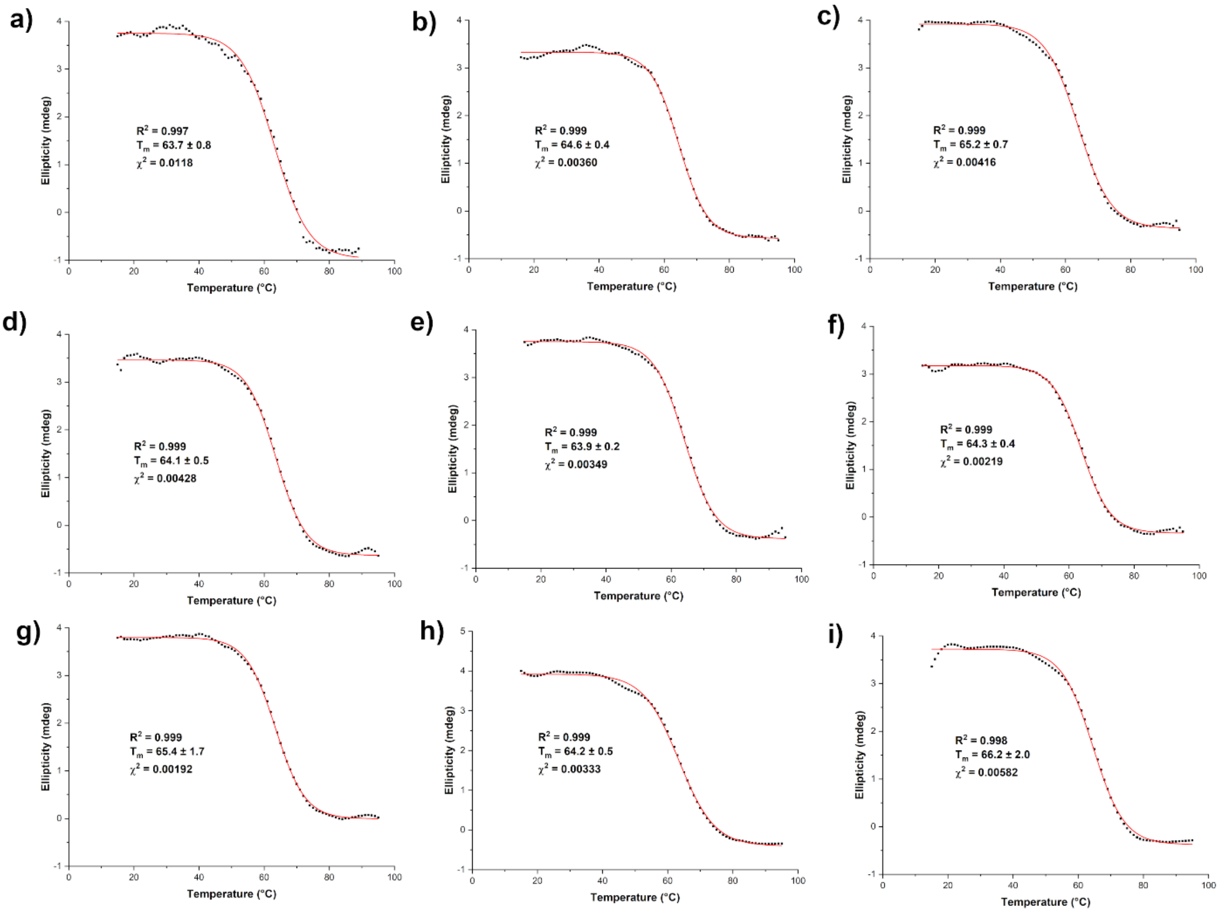


**Figure S19**: Each of the h-TELO-NDI melting plots (5 μM DNA with 5 equivalents of NDI): **a**) h-TELO only; **b**) h-TELO + **1**; **c**) h-TELO + **(*S*)-2**; **d**) h-TELO + **(*R*)-3**; **e**) h-TELO + **(*S*)-3**; **f**) h-TELO + **(*R*)-4**; **g**) h-TELO + **(*S*)-4**; **h**) h-TELO + **(*R*)-5**; and **i**) h-TELO + **(*S*)-5**. These plots are averaged of at least three separate experimental runs.

## 3.3 TBA

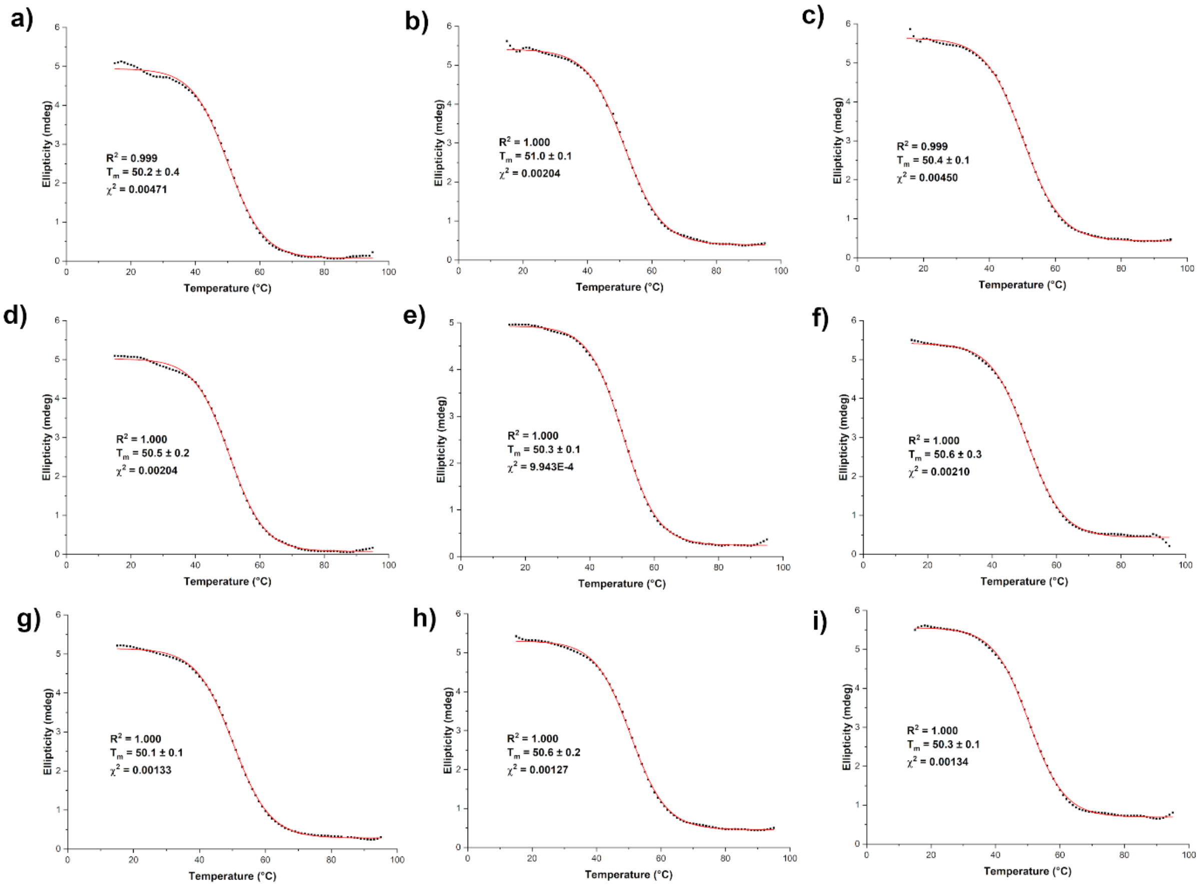


**Figure S20**: Each of the TBA-NDI melting plots (2 μM DNA with 5 equivalents of NDI): **a**) TBA only; **b**) TBA + **1**; **c**) TBA + **(*S*)-2**; **d**) TBA + **(*R*)-3**; **e**) TBA + **(*S*)-3**; **f**) TBA + **(*R*)-4**; **g**) TBA + **(*S*)-4**; **h**) TBA + **(*R*)-5**; and **i**) TBA + **(*S*)-5**. These plots are averaged of at least three separate experimental runs.

### 3.4 dsDNA

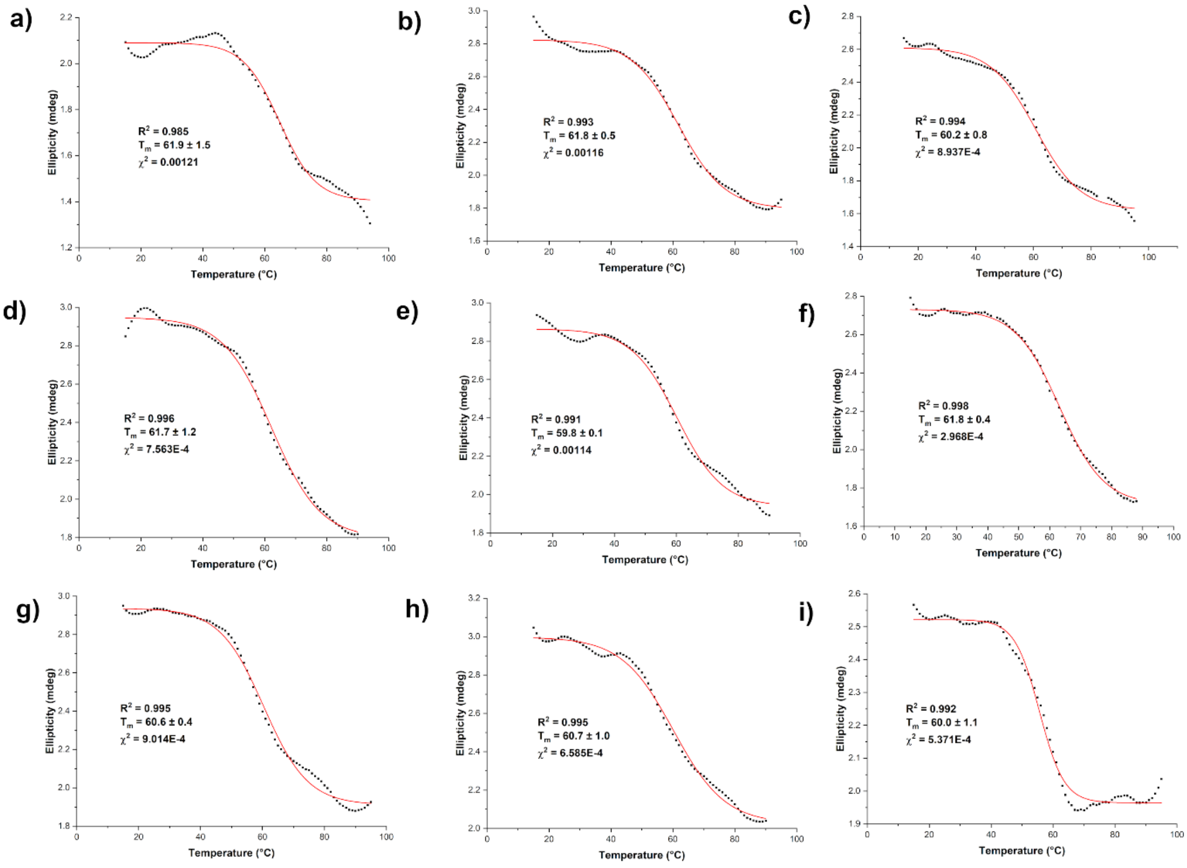


**Figure S21**: Each of the dsDNA-NDI melting plots (2 μM DNA with 5 equivalents of NDI): **a**) dsDNA only; **b**) dsDNA + **1**; **c**) dsDNA + **(*S*)-2**; **d**) dsDNA + **(*R*)-3**; **e**) dsDNA + **(*S*)-3**; **f**) dsDNA + **(*R*)-4**; **g**) dsDNA + **(*S*)-4**; **h**) dsDNA + **(*R*)-5**; and **i**) dsDNA + **(*S*)-5**. These plots are averaged of at least three separate experimental runs.

## 4. Plots of the Average $T_m$ with Standard Errors

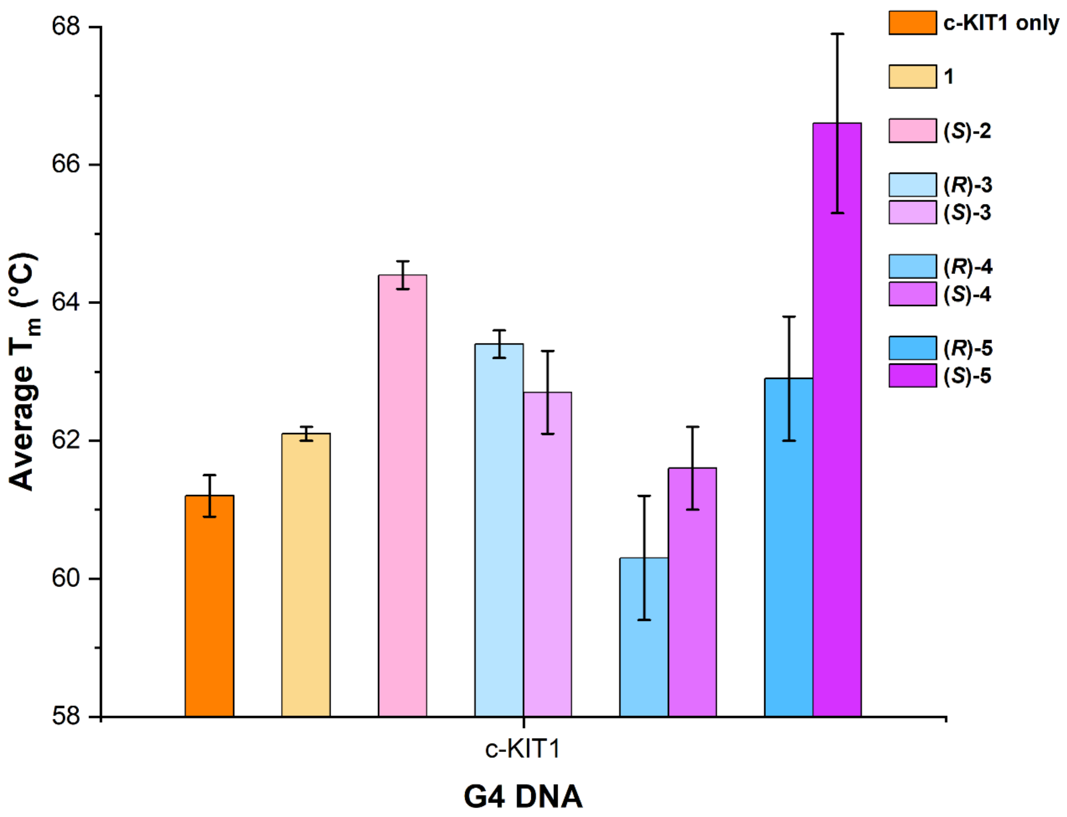


**Figure S22**: Plot of the average $T_m$ of c-KIT1 with each of the NDIs from two or more repeats, showing the standard error.

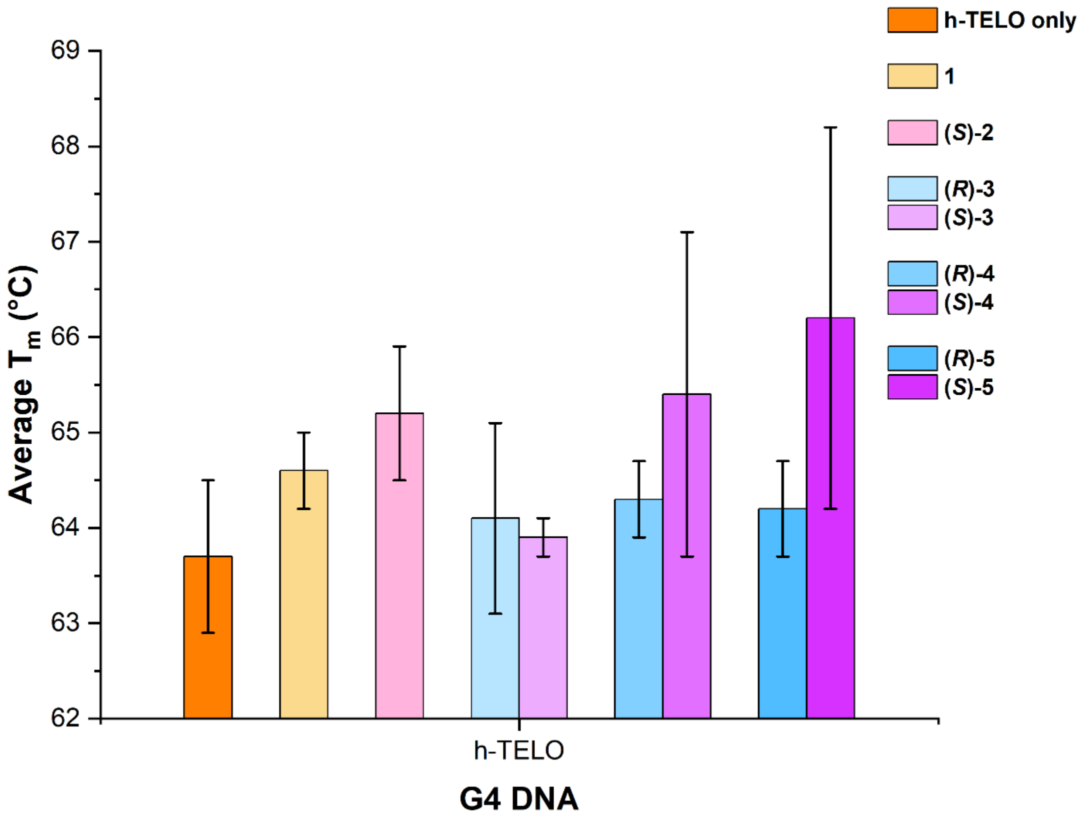


**Figure S23**: Plot of the average $T_m$ of h-TELO with each of the NDIs from two or more repeats, showing the standard error.

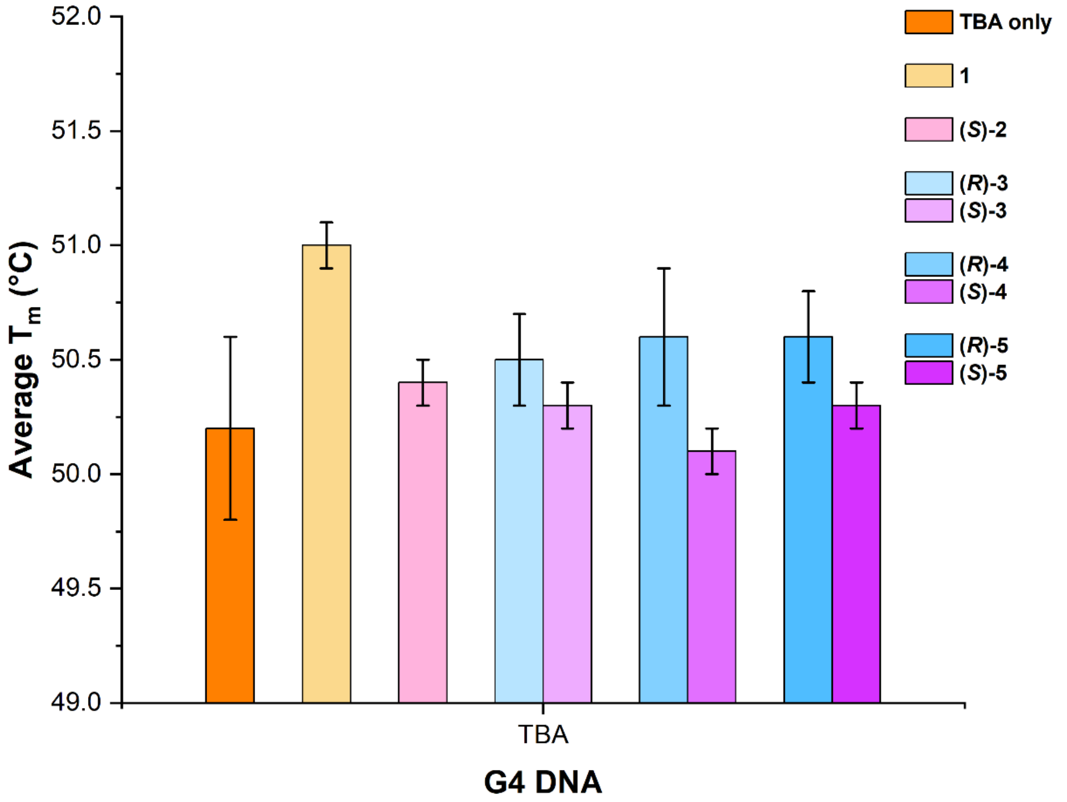


**Figure S24**: Plot of the average $T_m$ of TBA with each of the NDIs from two or more repeats, showing the standard error.

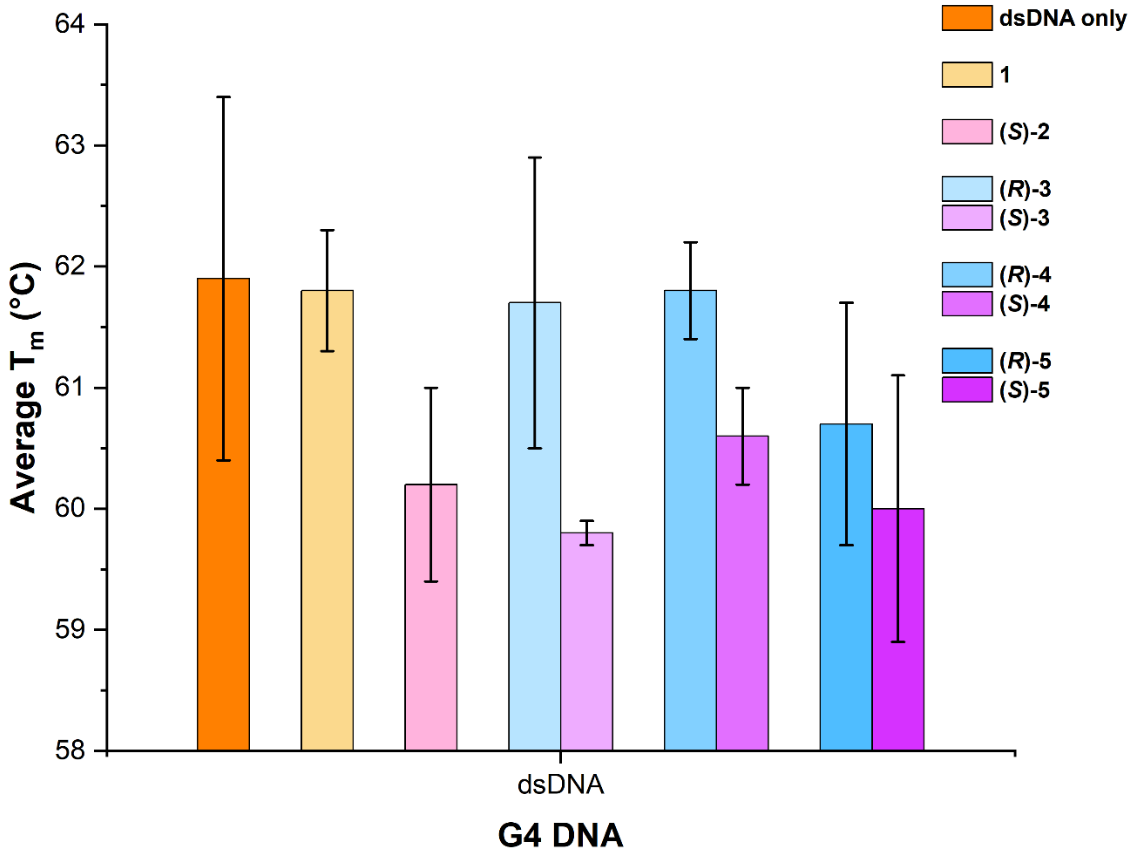


**Figure S25**: Plot of the average $T_m$ of dsDNA with each of the NDIs from two or more repeats, showing the standard error.

## 5. DNA-NDI Titration Fits

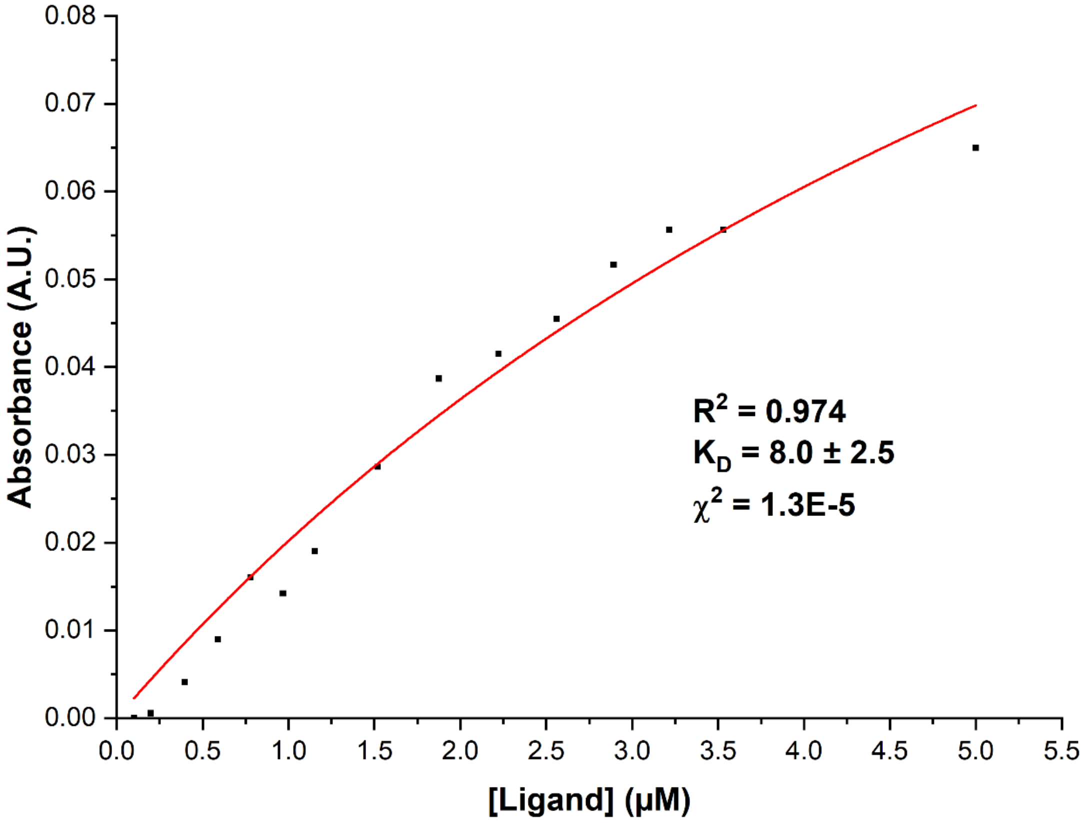


**Figure S26:** Dissociation fit from a titration of 2 μM c-KIT1 with **(*R*)-5** at 238 nm.

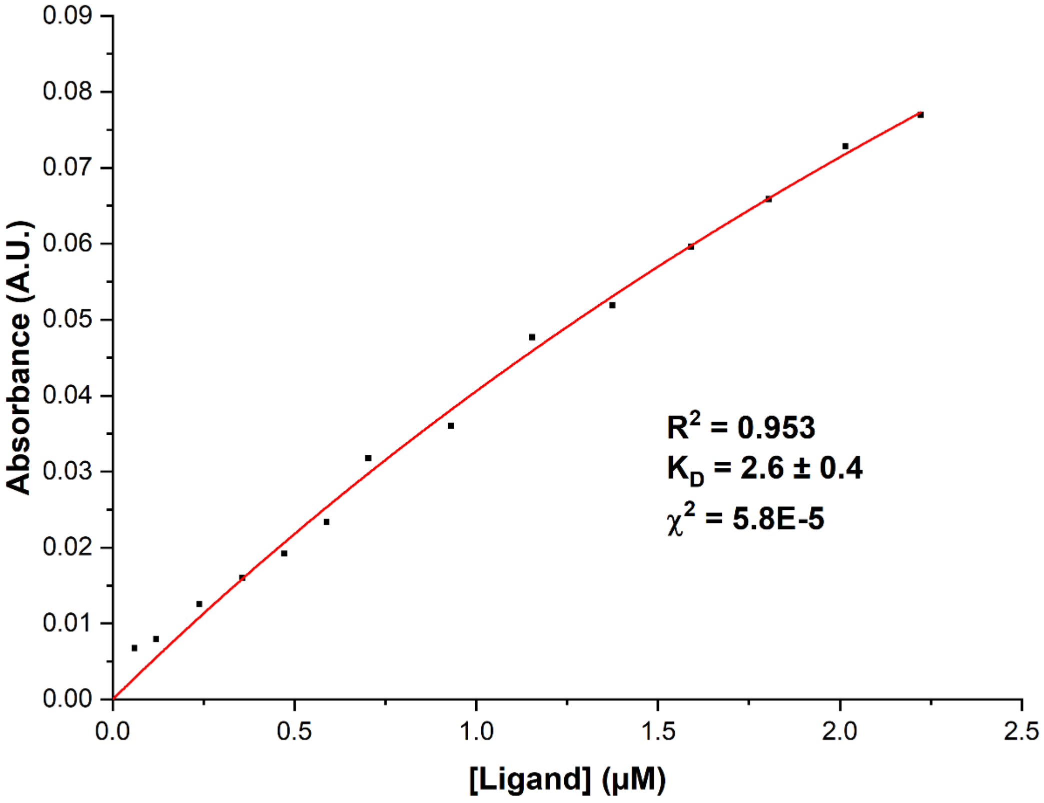


**Figure S27:** Dissociation fit from a titration of 2 μM c-KIT1 with **(*S*)-5** at 238 nm.

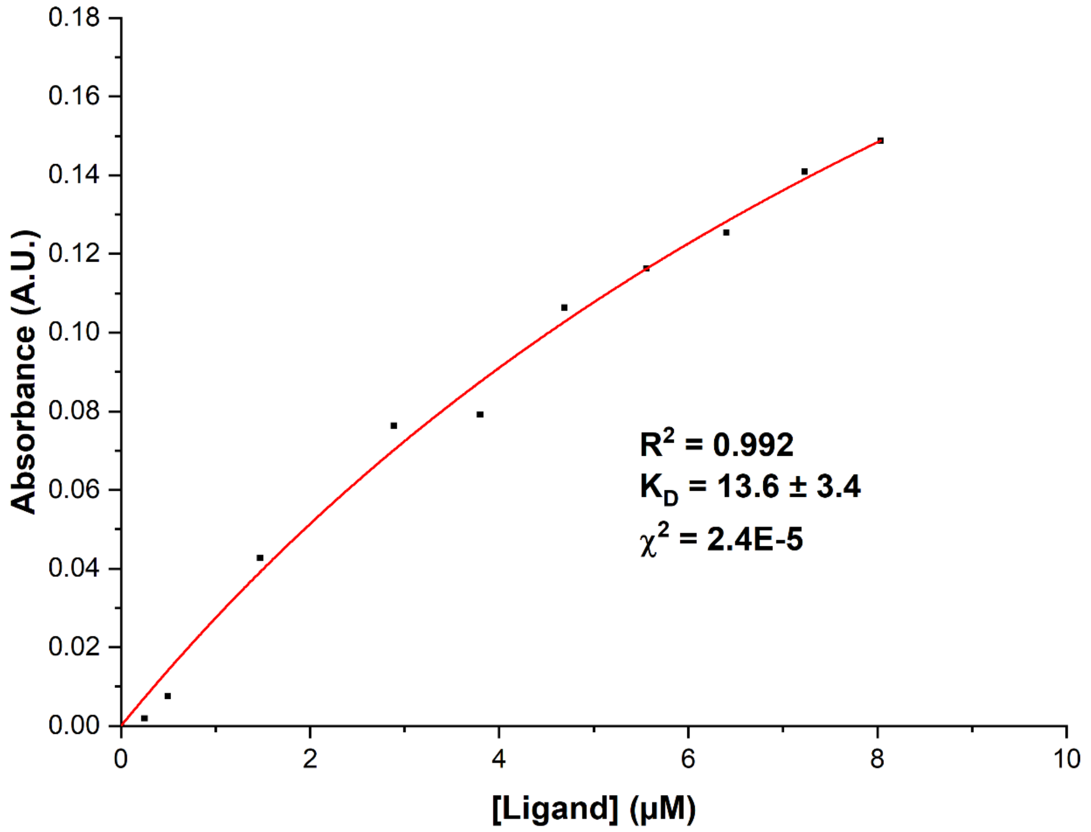


**Figure S28**: Dissociation fit from a titration of 5 μM h-TELO with **(*R*)-4** at 383 nm.

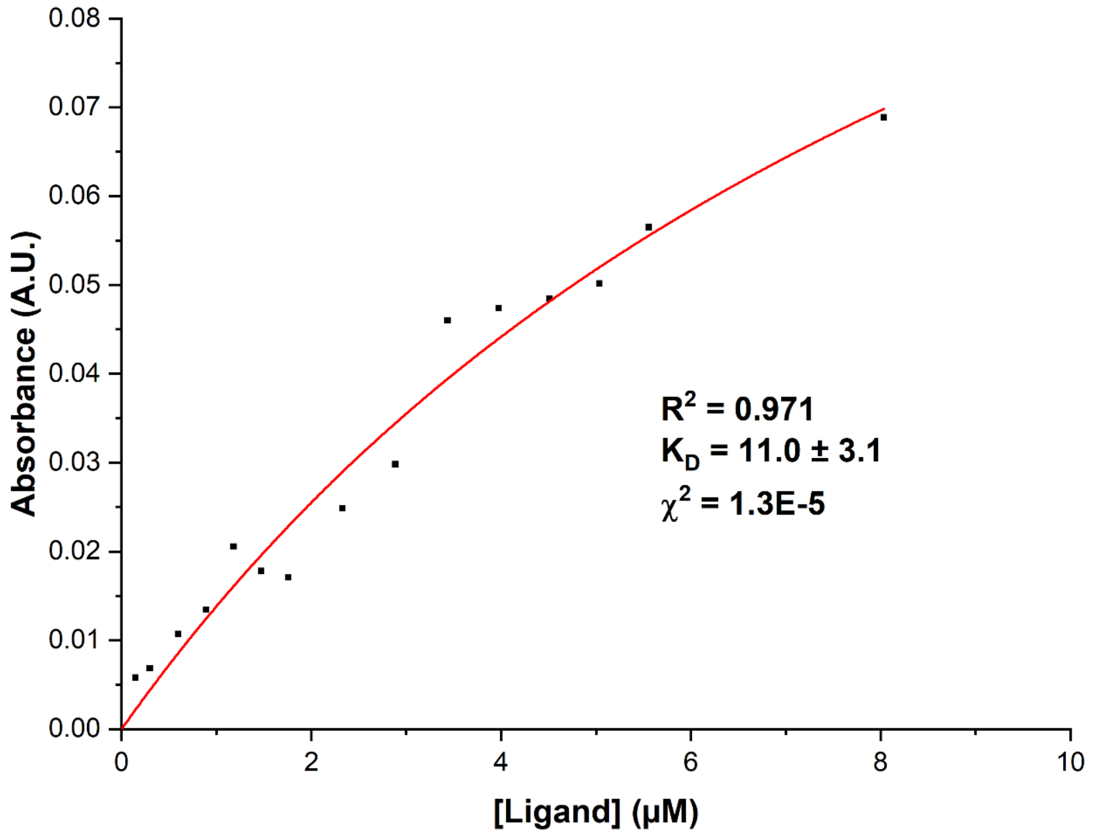


**Figure S29:** Dissociation fit from a titration of 5 μM h-TELO with **(*S*)-4** at 383 nm.

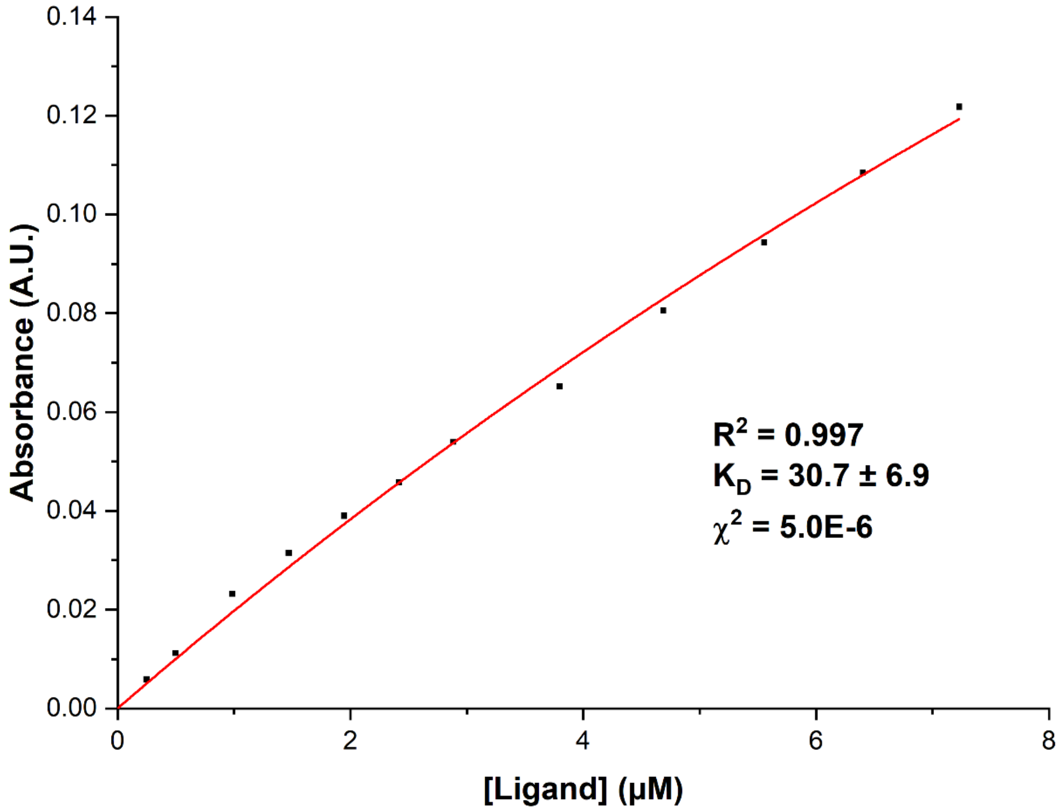


**Figure S30**: Dissociation fit from a titration of 5 μM dsDNA with **(*R*)-5** at 383 nm.

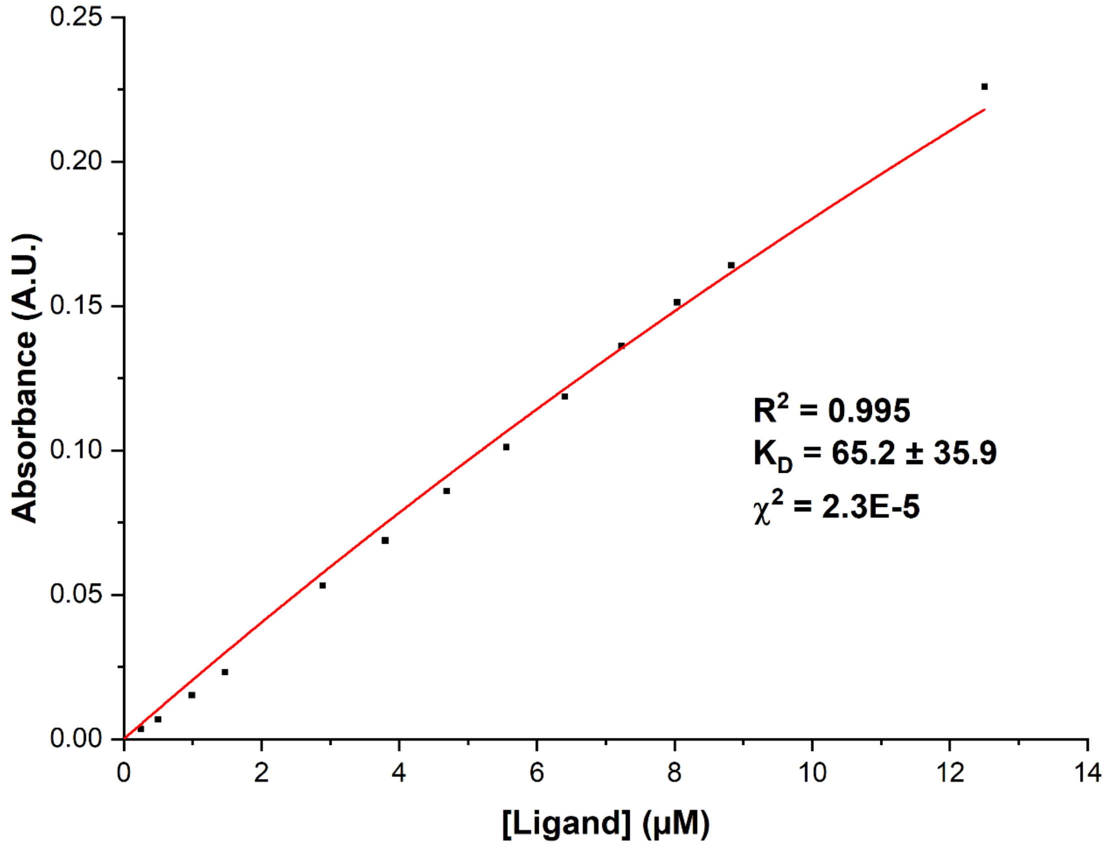


**Figure S31**: Dissociation fit from a titration of 5 μM dsDNA with **(*S*)-5** at 383 nm.

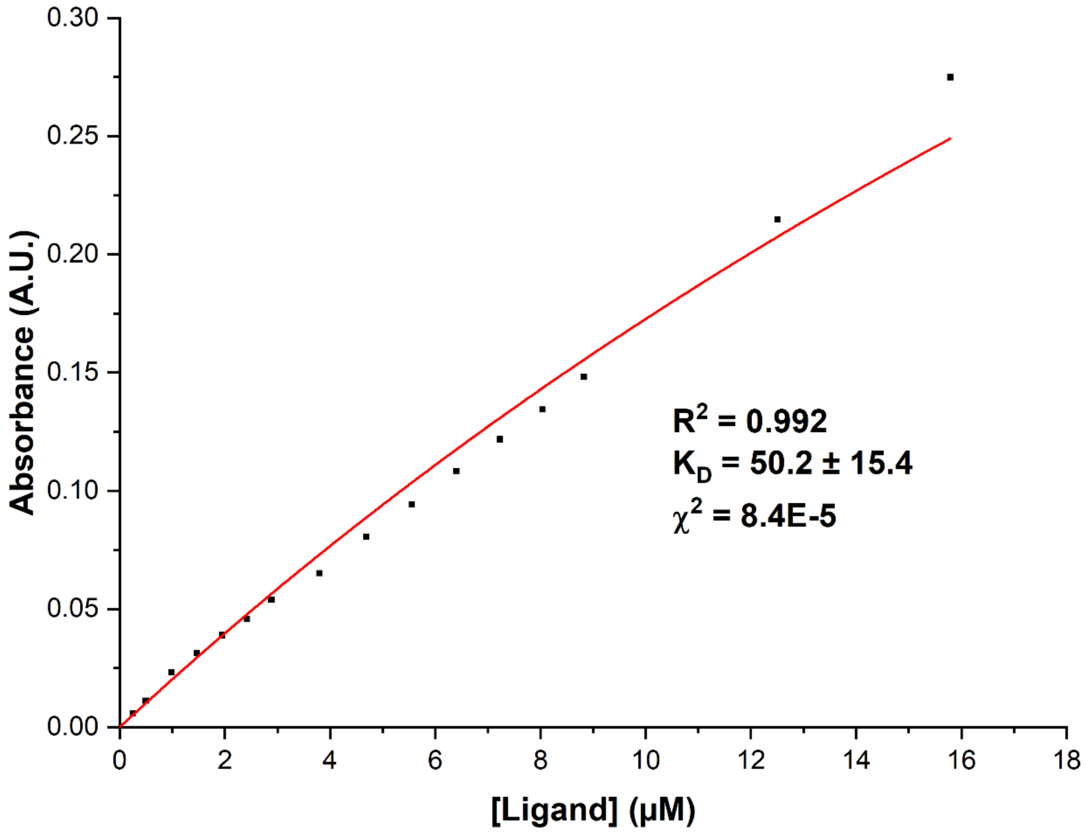


**Figure S32**: Dissociation fit from a titration of 5 μM dsDNA with **(*R*)-4** at 383 nm.

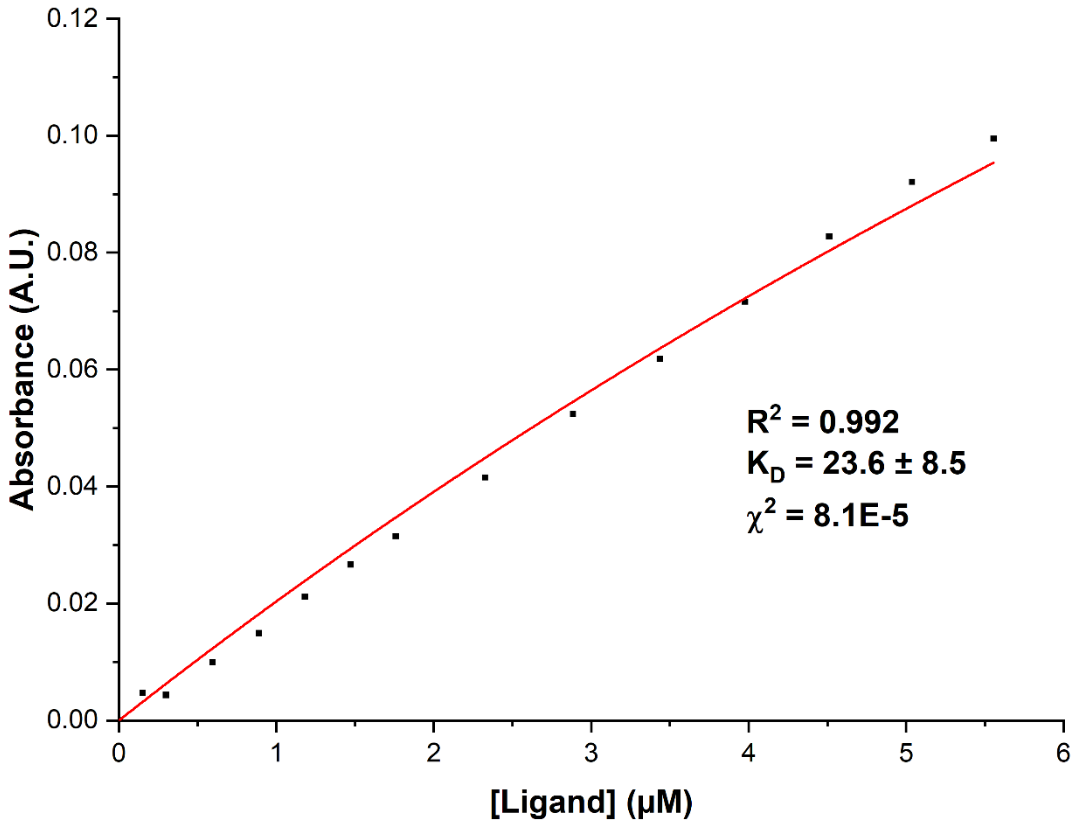


**Figure S33:** Dissociation fit from a titration of 5 μM dsDNA with **(*S*)-4** at 383 nm.

## 6. Mass Spectrometry Data

**Compound isotope peak List**

| m/z | z | Abund | Formula | Ion |
|---|---|---|---|---|
| 623.1666 | 1 | 1664786.6 | C34H26N2O10 | (M+H)+ |
| 624.1701 | 1 | 629368.6 | C34H26N2O10 | (M+H)+ |
| 625.1729 | 1 | 144747.5 | C34H26N2O10 | (M+H)+ |
| 626.1766 | 1 | 26741.8 | C34H26N2O10 | (M+H)+ |
| 627.1790 | 1 | 4494.7 | C34H26N2O10 | (M+H)+ |
| 645.1488 | 1 | 244105.0 | C34H26N2O10 | (M+Na)+ |
| 646.1517 | 1 | 96441.1 | C34H26N2O10 | (M+Na)+ |
| 647.1483 | 1 | 26624.9 | C34H26N2O10 | (M+Na)+ |
| 648.1439 | 1 | 5667.4 | C34H26N2O10 | (M+Na)+ |

**Compound Table**

| Compound Label | RT (min) | Observed mass (m/z) | Neutral observed mass (Da) | Theoretical mass (Da) | Mass error (ppm) | Isotope match score (%) |
|---|---|---|---|---|---|---|
| Cpd 1: C34 H26 N2 O10 | 0.86 | 623.1666 | 622.1593 | 622.1587 | 0.93 | 99.78 |

Mass errors of between -5.00 and 5.00 ppm with isotope match scores above 60% are considered confirmation of molecular formulae

**Figure: Extracted ion chromatogram (EIC) of compound.**

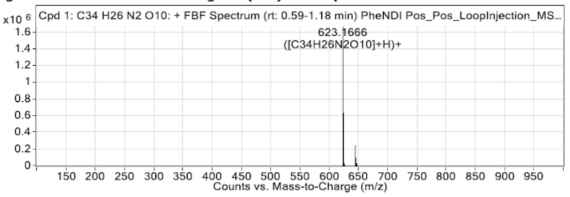


**Figure: Full range view of Compound spectra and potential adducts.**

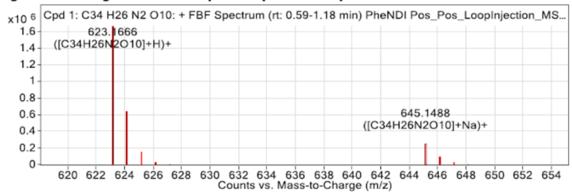


**Figure S34**: High-resolution mass spectrometry (HRMS) data for **1** with the (M+H)+ peak found at 623.1666.

**Compound isotope peak List**

| m/z | z | Abund | Formula | Ion |
|---|---|---|---|---|
| 443.0731 | 1 | 256811.7 | C20H14N2O10 | (M+H)+ |
| 444.0763 | 1 | 61839.5 | C20H14N2O10 | (M+H)+ |
| 445.0784 | 1 | 12113.9 | C20H14N2O10 | (M+H)+ |
| 446.0778 | 1 | 2207.7 | C20H14N2O10 | (M+H)+ |
| 465.0546 | 1 | 49635.7 | C20H14N2O10 | (M+Na)+ |
| 466.0581 | 1 | 12077.3 | C20H14N2O10 | (M+Na)+ |
| 467.0334 | 1 | 9596.7 | C20H14N2O10 | (M+Na)+ |

**Compound Table**

| Compound Label | RT (min) | Observed mass (m/z) | Neutral observed mass (Da) | Theoretical mass (Da) | Mass error (ppm) | Isotope match score (%) |
|---|---|---|---|---|---|---|
| Cpd 1: C20 H14 N2 O10 | 0.80 | 443.0731 | 442.0651 | 442.0648 | 0.57 | 99.54 |

Mass errors of between -5.00 and 5.00 ppm with isotope match scores above 60% are considered confirmation of molecular formulae

**Figure: Extracted ion chromatogram (EIC) of compound.**

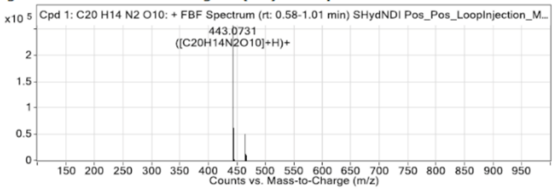


**Figure: Full range view of Compound spectra and potential adducts.**

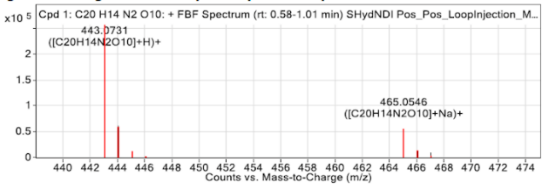


**Figure S35**: HRMS data for **(*S*)-2** with the (M+H)+ peak found at 443.0731.

**Compound isotope peak List**

| m/z | z | Abund | Formula | Ion |
|---|---|---|---|---|
| 439.1139 | 1 | 206310.9 | C22H18N2O8 | (M+H)+ |
| 440.1168 | 1 | 49902.6 | C22H18N2O8 | (M+H)+ |
| 441.1192 | 1 | 8909.8 | C22H18N2O8 | (M+H)+ |
| 442.1216 | 1 | 1176.7 | C22H18N2O8 | (M+H)+ |
| 461.0957 | 1 | 95589.6 | C22H18N2O8 | (M+Na)+ |
| 462.0986 | 1 | 23125.4 | C22H18N2O8 | (M+Na)+ |
| 463.1024 | 1 | 4163.6 | C22H18N2O8 | (M+Na)+ |
| 464.1048 | 1 | 634.2 | C22H18N2O8 | (M+Na)+ |

**Compound Table**

| Compound Label | RT (min) | Observed mass (m/z) | Neutral observed mass (Da) | Theoretical mass (Da) | Mass error (ppm) | Isotope match score (%) |
|---|---|---|---|---|---|---|
| Cpd 1: C22 H18 N2 O8 | 0.69 | 439.1139 | 438.1065 | 438.1063 | 0.51 | 99.54 |

Mass errors of between -5.00 and 5.00 ppm with isotope match scores above 60% are considered confirmation of molecular formulae

**Figure: Extracted ion chromatogram (EIC) of compound.**

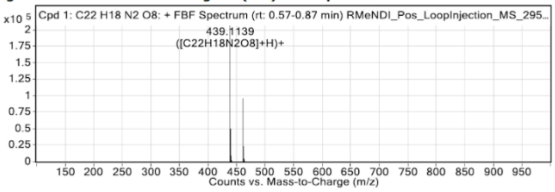


**Figure: Full range view of Compound spectra and potential adducts.**

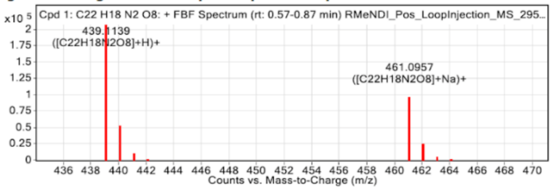


**Figure S36**: HRMS data for **(*R*)-3** with the (M+H)+ peak found at 439.1139.

**Compound isotope peak List**

| m/z | z | Abund | Formula | Ion |
|---|---|---|---|---|
| 439.1144 | 1 | 1078810.0 | C22H18N2O8 | (M+H)+ |
| 440.1180 | 1 | 265422.4 | C22H18N2O8 | (M+H)+ |
| 441.1204 | 1 | 50323.6 | C22H18N2O8 | (M+H)+ |
| 442.1232 | 1 | 7439.9 | C22H18N2O8 | (M+H)+ |
| 461.0966 | 1 | 168207.9 | C22H18N2O8 | (M+Na)+ |
| 462.0996 | 1 | 43723.7 | C22H18N2O8 | (M+Na)+ |
| 463.1014 | 1 | 8354.7 | C22H18N2O8 | (M+Na)+ |

**Compound Table**

| Compound Label | RT (min) | Observed mass (m/z) | Neutral observed mass (Da) | Theoretical mass (Da) | Mass error (ppm) | Isotope match score (%) |
|---|---|---|---|---|---|---|
| Cpd 1: C22 H18 N2 O8 | 0.72 | 439.1144 | 438.1072 | 438.1063 | 2.05 | 99.53 |

Mass errors of between -5.00 and 5.00 ppm with isotope match scores above 60% are considered confirmation of molecular formulae

**Figure: Extracted ion chromatogram (EIC) of compound.**

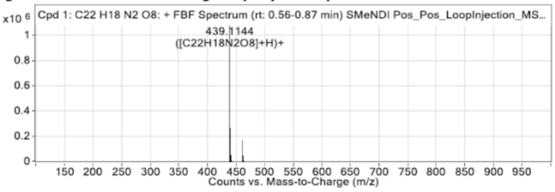


**Figure: Full range view of Compound spectra and potential adducts.**

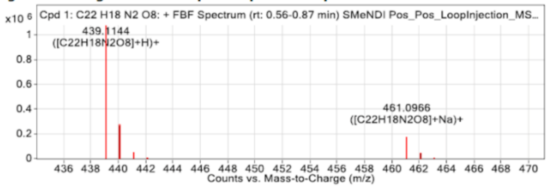


**Figure S37**: HRMS data for **(*S*)-3** with the $(M+H)^+$ peak found at 439.1144.

**Compound isotope peak List**

| m/z | z | Abund | Formula | Ion |
|---|---|---|---|---|
| 523.2081 | 1 | 2653784.5 | C28H30N2O8 | (M+H)+ |
| 524.2115 | 1 | 804378.6 | C28H30N2O8 | (M+H)+ |
| 525.2143 | 1 | 156314.2 | C28H30N2O8 | (M+H)+ |
| 526.2168 | 1 | 25498.0 | C28H30N2O8 | (M+H)+ |
| 527.2194 | 1 | 3220.7 | C28H30N2O8 | (M+H)+ |
| 545.1900 | 1 | 662674.4 | C28H30N2O8 | (M+Na)+ |
| 546.1934 | 1 | 209638.7 | C28H30N2O8 | (M+Na)+ |
| 547.1959 | 1 | 43915.6 | C28H30N2O8 | (M+Na)+ |
| 548.1987 | 1 | 7257.3 | C28H30N2O8 | (M+Na)+ |

**Compound Table**

| Compound Label | RT (min) | Observed mass (m/z) | Neutral observed mass (Da) | Theoretical mass (Da) | Mass error (ppm) | Isotope match score (%) |
|---|---|---|---|---|---|---|
| Cpd 1: C28 H30 N2 O8 | 0.75 | 545.1900 | 522.2008 | 522.2002 | 1.19 | 99.92 |

Mass errors of between -5.00 and 5.00 ppm with isotope match scores above 60% are considered confirmation of molecular formulae

**Figure: Extracted ion chromatogram (EIC) of compound.**

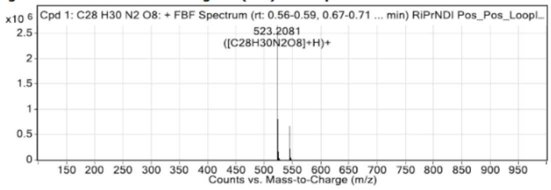


**Figure: Full range view of Compound spectra and potential adducts.**

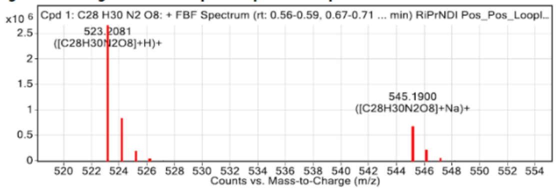


**Figure S38**: HRMS data for **(*R*)-4** with the $(M+H)^+$ peak found at 523.2081.

**Compound isotope peak List**

| m/z | z | Abund | Formula | Ion |
|---|---|---|---|---|
| 523.2077 | 1 | 490884.3 | C28H30N2O8 | (M+H)+ |
| 524.2108 | 1 | 143497.1 | C28H30N2O8 | (M+H)+ |
| 525.2131 | 1 | 27406.5 | C28H30N2O8 | (M+H)+ |
| 526.2154 | 1 | 4084.7 | C28H30N2O8 | (M+H)+ |
| 527.2198 | 1 | 568.0 | C28H30N2O8 | (M+H)+ |
| 545.1895 | 1 | 144514.1 | C28H30N2O8 | (M+Na)+ |
| 546.1924 | 1 | 41381.6 | C28H30N2O8 | (M+Na)+ |
| 547.1948 | 1 | 8551.0 | C28H30N2O8 | (M+Na)+ |
| 548.1969 | 1 | 1331.6 | C28H30N2O8 | (M+Na)+ |
| 549.2176 | 1 | 198.9 | C28H30N2O8 | (M+Na)+ |

**Compound Table**

| Compound Label | RT (min) | Observed mass (m/z) | Neutral observed mass (Da) | Theoretical mass (Da) | Mass error (ppm) | Isotope match score (%) |
|---|---|---|---|---|---|---|
| Cpd 1: C28 H30 N2 O8 | 0.74 | 523.2077 | 522.2003 | 522.2002 | 0.21 | 98.34 |

Mass errors of between -5.00 and 5.00 ppm with isotope match scores above 60% are considered confirmation of molecular formulae

**Figure: Extracted ion chromatogram (EIC) of compound.**

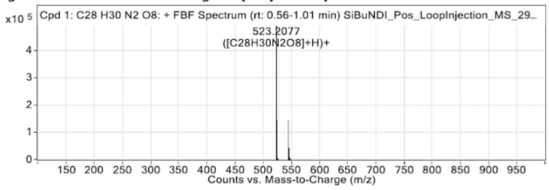


**Figure: Full range view of Compound spectra and potential adducts.**

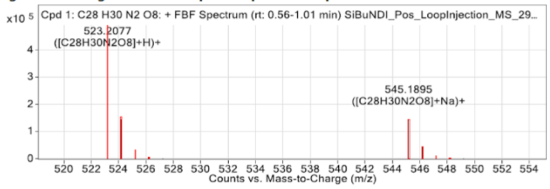


**Figure S39**: HRMS data for **(*S*)-4** with the $(M+H)^+$ peak found at 523.2077.

**Compound Table**

| Compound Label | RT (min) | Observed mass (m/z) | Neutral observed mass (Da) | Theoretical mass (Da) | Mass error (ppm) | Isotope match score (%) |
|---|---|---|---|---|---|---|
| Cpd 1: C30 H32 N4 O12 | 0.62 | 663.1911 | 640.2019 | 640.2017 | 0.34 | 98.87 |

Mass errors of between -5.00 and 5.00 ppm with isotope match scores above 60% are considered confirmation of molecular formulae

**Compound isotope peak List**

| m/z | z | Abund | Formula | Ion |
|---|---|---|---|---|
| 641.2265 | 1 | 575.2 | C30H32N4O12 | (M+H)+ |
| 642.2300 | 1 | 459.5 | C30H32N4O12 | (M+H)+ |
| 663.1911 | 1 | 55177.0 | C30H32N4O12 | (M+Na)+ |
| 664.1936 | 1 | 18582.3 | C30H32N4O12 | (M+Na)+ |
| 665.1956 | 1 | 4338.5 | C30H32N4O12 | (M+Na)+ |
| 666.2025 | 1 | 722.8 | C30H32N4O12 | (M+Na)+ |

**Figure: Extracted ion chromatogram (EIC) of compound.**

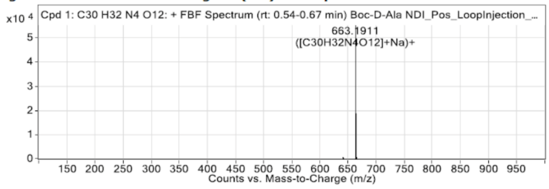


**Figure: Full range view of Compound spectra and potential adducts.**

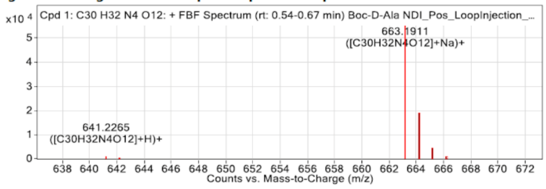


**Figure S40**: HRMS data for ***(R)*-5** with the $(M+Na)^{+}$ peak found at 663.1911.

**Compound Table**

| Compound Label | RT (min) | Observed mass (m/z) | Neutral observed mass (Da) | Theoretical mass (Da) | Mass error (ppm) | Isotope match score (%) |
|---|---|---|---|---|---|---|
| Cpd 1: C30 H32 N4 O12 | 0.85 | 663.1912 | 640.2016 | 640.2017 | -0.12 | 97.55 |

Mass errors of between -5.00 and 5.00 ppm with isotope match scores above 60% are considered confirmation of molecular formulae

**Compound isotope peak List**

| m/z | z | Abund | Formula | Ion |
|---|---|---|---|---|
| 641.1981 | 1 | 1170.5 | C30H32N4O12 | (M+H)+ |
| 642.2027 | 1 | 509.6 | C30H32N4O12 | (M+H)+ |
| 643.1688 | 1 | 526.9 | C30H32N4O12 | (M+H)+ |
| 663.1912 | 1 | 177091.5 | C30H32N4O12 | (M+Na)+ |
| 664.1938 | 1 | 56406.9 | C30H32N4O12 | (M+Na)+ |
| 665.1959 | 1 | 13059.6 | C30H32N4O12 | (M+Na)+ |
| 666.1990 | 1 | 2357.4 | C30H32N4O12 | (M+Na)+ |
| 667.2011 | 1 | 384.9 | C30H32N4O12 | (M+Na)+ |

**Figure: Extracted ion chromatogram (EIC) of compound.**

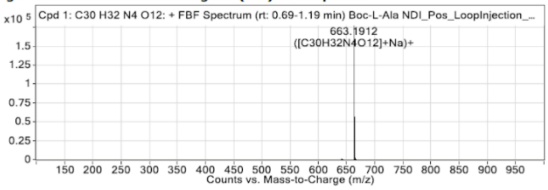


**Figure: Full range view of Compound spectra and potential adducts.**

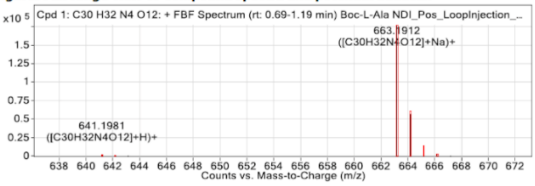


**Figure S41**: HRMS data for ***(S)*-5** with the $(M+Na)^{+}$ peak found at 663.1912.

## 7. HPLC Data

**Table S2**: Reverse-phase HPLC gradient method for all NDIs.

| **Time (mins)** | **$CH_3CN$+0.1HCOOH (%)** | **$H_2O$+0.1HCOOH (%)** | **Flow Rate** |
|---|---|---|---|
| 0 | 15 | 85 | 0.5 |
| 16 | 80 | 20 | 0.5 |
| 18 | 80 | 20 | 1.5 |
| 19 | 15 | 85 | 1.5 |

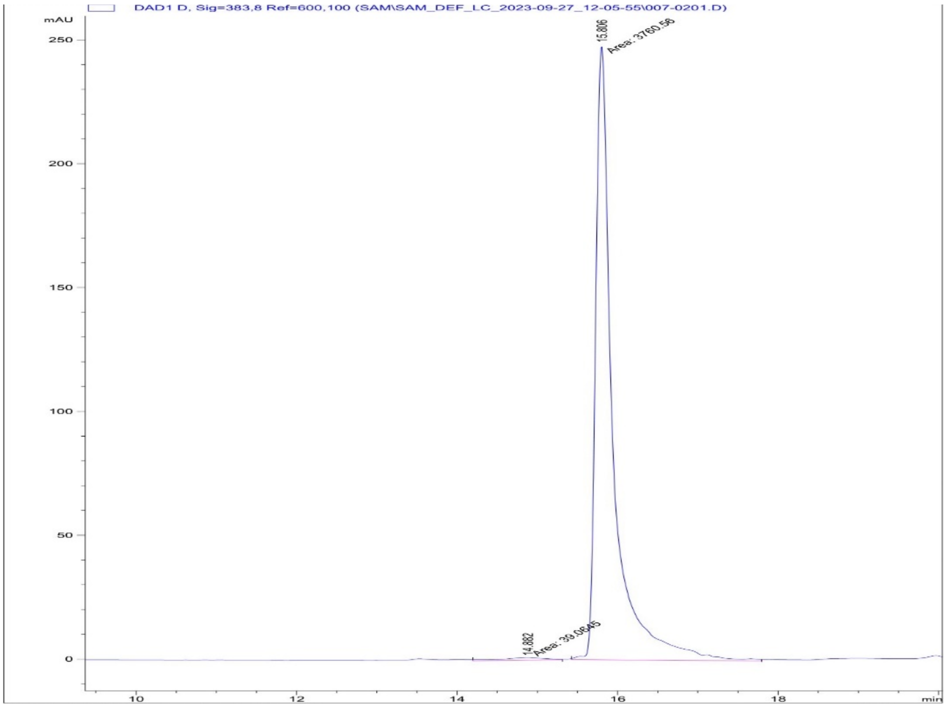


**Figure S42**: Reverse-phase HPLC analysis at 383 nm of **1**.

**Table S3**: The elution areas and times of the reverse-phase HPLC analysis at 383 nm of **1**.

| **Peak Number** | **Time (mins)** | **Area** | **Percentage Area** |
|---|---|---|---|
| 1 | 14.88 | 39.10 | 1.03 |
| 2 | 15.81 | 3760.60 | 98.97 |

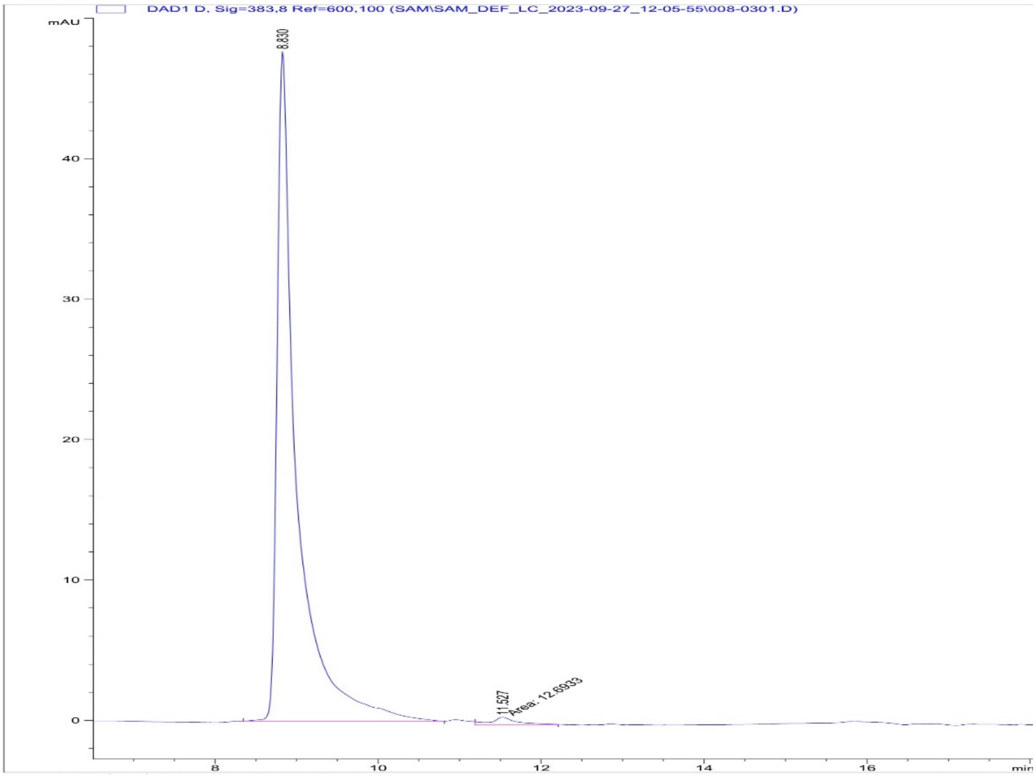


**Figure S43**: Reverse-phase HPLC analysis of **(*S*)-2**.

**Table S4**: The elution areas and times of the reverse-phase HPLC analysis at 383 nm of **(*S*)-2**.

| Peak Number | Time (mins) | Area | Percentage Area |
|---|---|---|---|
| 1 | 8.83 | 794.40 | 98.43 |
| 2 | 11.53 | 12.7 | 1.57 |

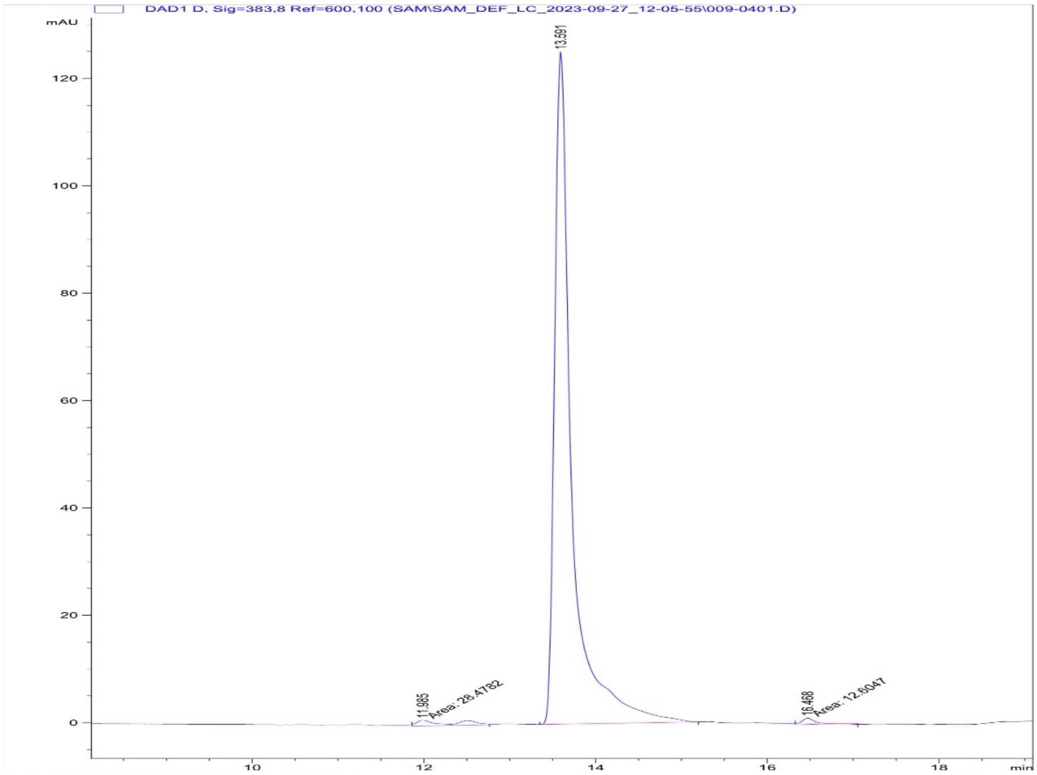


**Figure S44**: Reverse-phase HPLC analysis of **(*R*)-3**.

**Table S5**: The elution areas and times of the reverse-phase HPLC analysis at 383 nm of **(*R*)-3**.

| Peak Number | Time (mins) | Area | Percentage Area |
|---|---|---|---|
| 1 | 11.99 | 28.50 | 1.59 |
| 2 | 13.59 | 1746.40 | 97.70 |
| 3 | 16.47 | 12.60 | 0.71 |

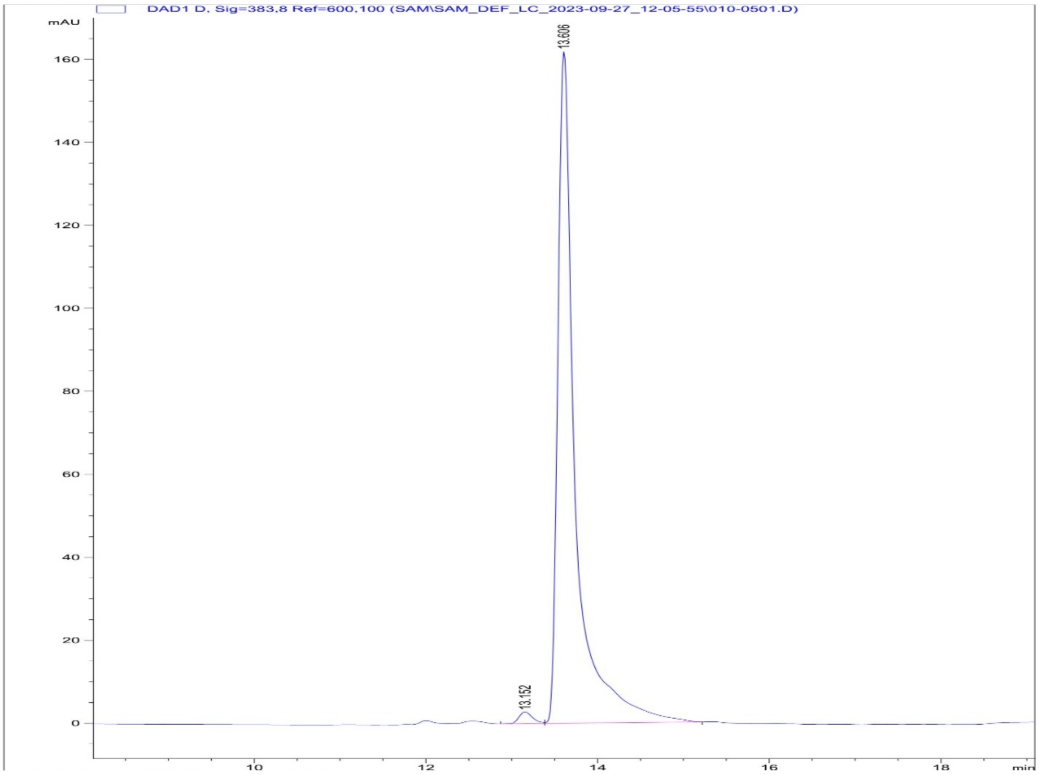


**Figure S45**: Reverse-phase HPLC analysis of **(*S*)-3**.

**Table S6**: The elution areas and times of the reverse-phase HPLC analysis at 383 nm of **(*S*)-3**.

| **Peak Number** | **Time (mins)** | **Area** | **Percentage Area** |
|---|---|---|---|
| 1 | 13.15 | 31.40 | 1.36 |
| 2 | 13.61 | 2286.30 | 98.64 |

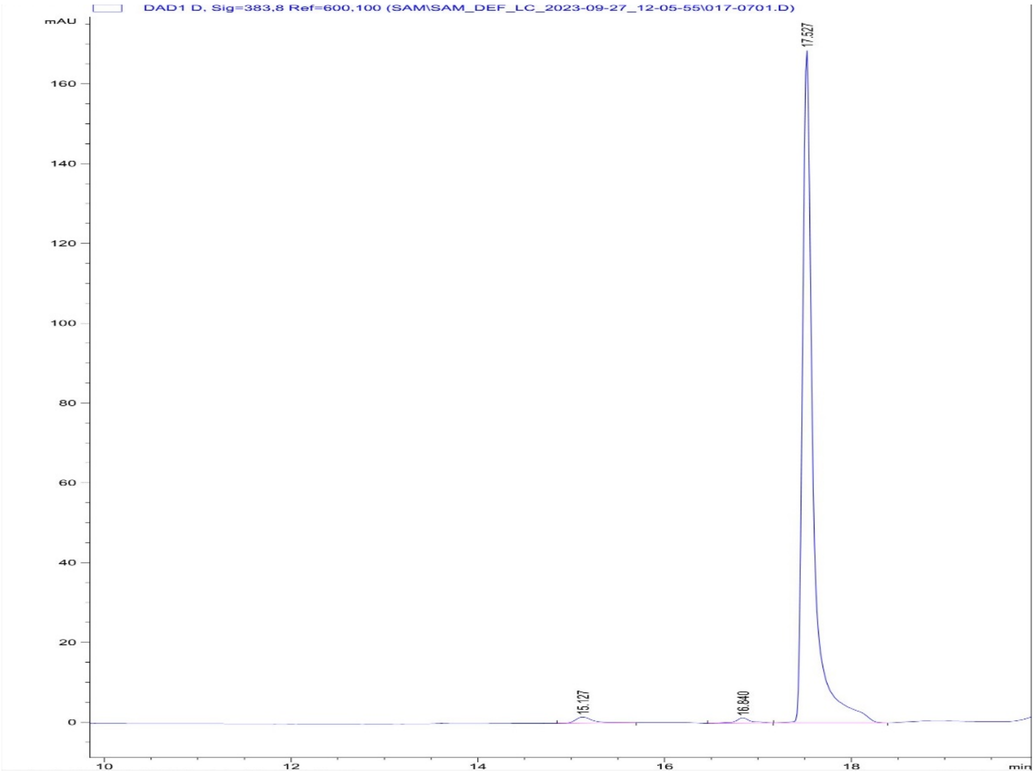


**Figure S46**: Reverse-phase HPLC analysis of **(*R*)-4**.

**Table S7**: The elution areas and times of the reverse-phase HPLC analysis at 383 nm of **(*R*)-4**.

| Peak Number | Time (mins) | Area | Percentage Area |
|---|---|---|---|
| 1 | 15.13 | 19.10 | 1.40 |
| 2 | 16.84 | 14.40 | 1.06 |
| 3 | 17.53 | 1334.80 | 99.01 |

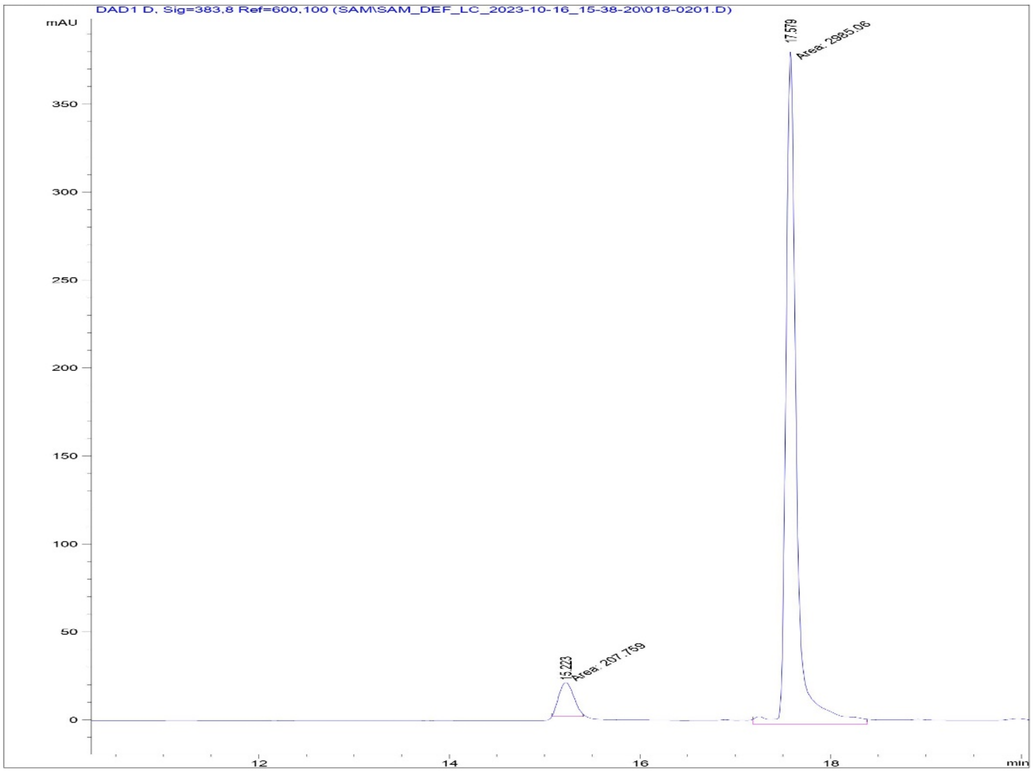


**Figure S47**: Reverse-phase HPLC analysis of **(*S*)-4**.

**Table S8**: The elution areas and times of the reverse-phase HPLC analysis at 383 nm of **(*S*)-4**.

| Peak Number | Time (mins) | Area | Percentage Area |
|---|---|---|---|
| 1 | 15.10 | 207.76 | 6.67 |
| 2 | 17.52 | 2985.06 | 93.32 |

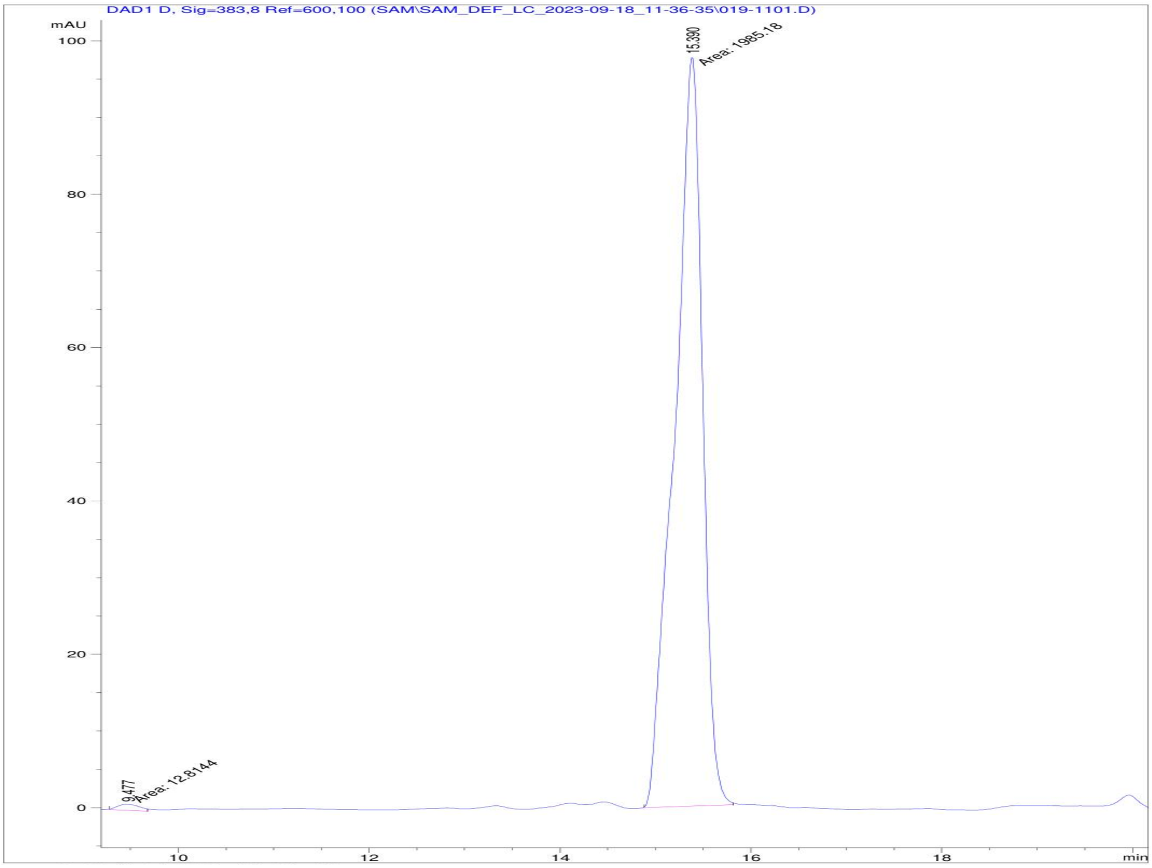


**Figure S48**: Reverse-phase HPLC analysis of ***(R)*-5**.

**Table S9**: The elution areas and times of the reverse-phase HPLC analysis at 383 nm of ***(R)*-5**.

| Peak Number | Time (mins) | Area | Percentage Area |
|---|---|---|---|
| 1 | 13.73 | 12.8 | 0.64 |
| 2 | 15.00 | 1985.2 | 99.36 |

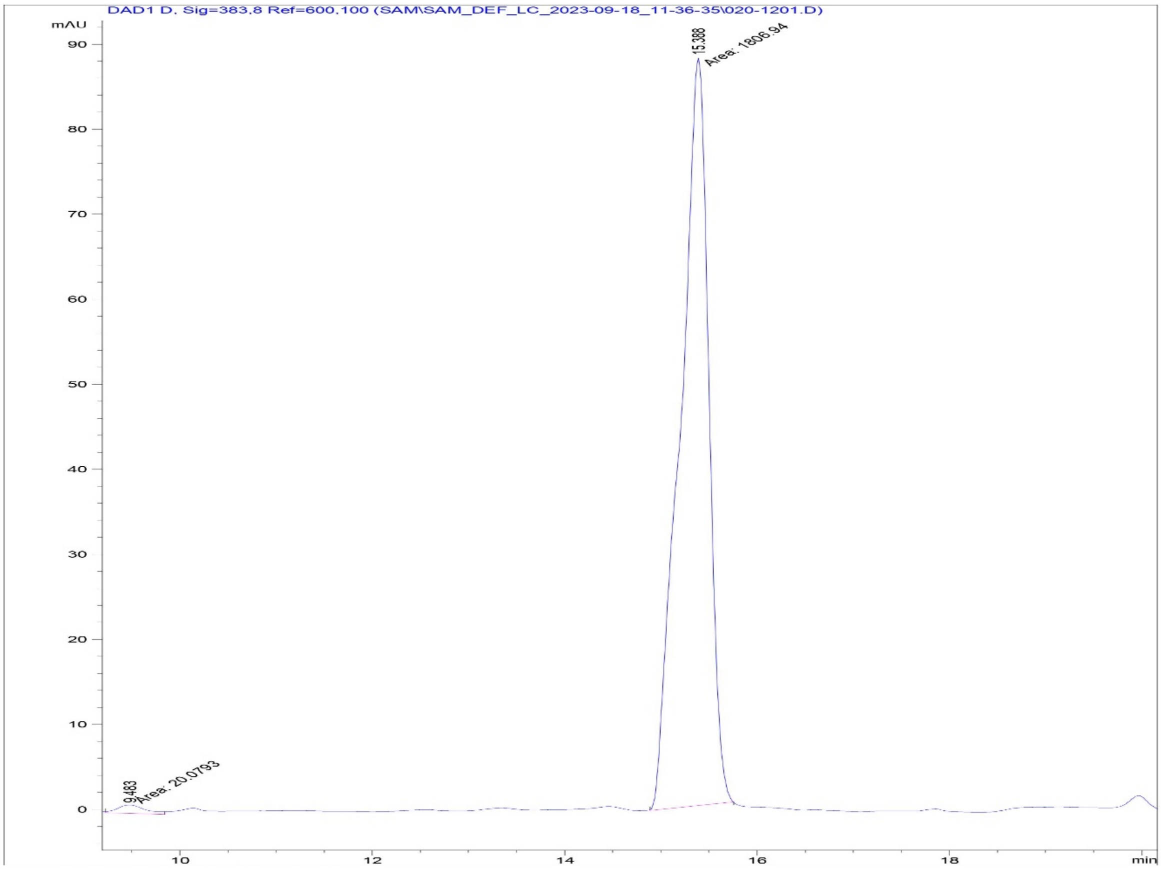


**Figure S49**: Reverse-phase HPLC analysis of **(*S*)-5**.

**Table S10**: The elution areas and times of the reverse-phase HPLC analysis at 383 nm of **(*S*)-5**.

| Peak Number | Time (mins) | Area | Percentage Area |
|---|---|---|---|
| 1 | 14.11 | 20.1 | 1.10 |
| 2 | 14.98 | 1806.9 | 98.90 |
